# Reduced intensity versus myeloablative conditioning for MDS: long-term results of an EBMT phase III study (RICMAC)

**DOI:** 10.1038/s41409-024-02282-7

**Published:** 2024-04-25

**Authors:** Christian Niederwieser, Simona Iacobelli, Georg-Nikolaus Franke, Linda Koster, Marleen van Os, Uwe Platzbecker, Kai Hübel, Christof Scheid, Lutz Peter Müller, Matthias Stelljes, Elena Morozova, Jakob Passweg, Francesco Onida, Peter Dreger, Riccardo Saccardi, Marco Ladetto, Urpu Salmenniemi, Wolfgang Bethge, Xavier Poiré, Guido Kobbe, Donal P. McLornan, Marie Robin, Nicolaus Kröger

**Affiliations:** 1 University Medical Center Hamburg-Eppendorf, Department of Stem Cell Transplantation, Hamburg, Germany; 2 Università Tor Vergata, Rome, Italy; 3 University Hospital Leipzig, Leipzig, Germany; 4 EBMT Leiden Study Unit, Leiden, The Netherlands; 5 University of Cologne, Cologne, Germany; 6 University Hospital Halle (Saale), Department of Internal Medicine IV, Martin-Luther-University Halle-Wittenberg, Halle, Germany; 7 University of Münster, Münster, Germany; 8 RM Gorbacheva Research Institute, Pavlov University, Saint Petersburg, Russian Federation; 9 University Hospital Basel, Basel, Switzerland; 10 Fondazione IRCC Ca’ Granda Ospedale Maggiore Policlinico, University of Milano, Milan, Italy; 11 University of Heidelberg, Heidelberg, Germany; 12 Azienda Ospedaliera Universitaria Careggi, Florence, Italy; 13 Azienda Ospedaliera SS. Antonio e Biagio e Cesare Arrigo, Alessandria, Italy; 14 Helsinki University Hospital, Helsiniki, Finland; 15 University Hospital Tübingen, Department of Hematology, Oncology, Clinical Immunology and Rheumatology Tübingen, Tübingen, Germany; 16 Cliniques Universitaires St-Luc, Brussels, Belgium; 17 University Hospital Düsseldorf, Heinrich Heine University, Medical Faculty, Düsseldorf, Germany; 18 Department of Haematology and Stem Cell Transplantation, University College London Hospital, London, United Kingdom; 19 Hôpital Saint Louis, Assistance Publique Hôpitaux Paris, Université de Paris Cité, Paris, France

**Keywords:** Myelodysplastic syndrome, Stem-cell research

## Abstract

Short-term outcome of myeloablative (MAC) and reduced intensity (RIC) conditioning in the prospective randomized international EBMT RICMAC study in patients with myelodyplastic syndrome (MDS) was comparable but longer follow up is lacking. Patients with MDS aged 18–65 years were randomized to receive MAC (*N* = 64) with busulfan/cyclophosphamide or RIC (*n* = 65) with busulfan/fludarabine followed by stem cell transplantation -(HCT) from HLA matched or mismatched donor. After a median follow-up of 6.2 (0.4–12.5) years, 10-year OS and RFS were 54.0% and 43.9% for RIC and 44.4% and 44.2% for MAC (*p* = 0.15 and *p* = 0.78), respectively. Since the first report, 6 patients died on NRM, 4 after RIC, and 2 after MAC. Similarly, 8 patients relapsed (4 in each arm), increasing the number of relapsed patients to 28. The second HCT was performed in 18 pts, 8 in the MAC, and 10 in the RIC arm. In a multivariate analysis, ECOG status and chemotherapy prior to HCT were independent risk factors for OS and RFS, ECOG and low cytogenetic risk for NRM and chemotherapy prior to HCT for RI. Patients with low cytogenetic risk had better OS [*p* = 0.002], RFS [*p* = 0.02], and NRM (*p* = 0.015) after RIC as compared to MAC.

## Introduction

Myelodysplastic neoplasm (MDS) is a molecularly and morphologically heterogeneous disease presenting mainly in older patients with a median age of 76 years [1, 2]. Following new insights into the biology of the disease, exciting new treatment concepts become available, such as hypomethylating agents, molecular therapies, and growth hormones. While novel therapies have been shown to prolong survival, allogeneic hematopoietic cell transplantation (HCT) remains the treatment with the highest curative potential.

HCT after myeloablative conditioning (MAC) has been considered for decades the golden standard for younger patients and used with increasing frequency. From the early century, the use of HCT after reduced intensity conditioning (RIC) or non-myeloablative (NMA) conditioning opened the potential of curative treatment to elderly patients, the population with the highest incidence. However, the optimal conditioning regimen in younger patients has still to be defined. Several retrospective studies described similar results after HCT with RIC or MAC conditioning, but significant age differences were noted in both groups, and selection biases were assumed. Therefore, a randomized study comparing MAC and RIC [3] using a uniform protocol and GVHD-prophylaxis was performed in patients up to the age of 64 years. The short-term results of up to 24 months were published previously [3]. In the following analysis, we present a nonpreplanned analysis of long-term results with a follow-up period of up to 10 years investigating a possible higher late relapse rate after RIC or differences in the outcome of subgroups of patients with different disease risks. The clinical trial was registered as NCT01203228.

## Patients and methods

In this prospective, multicenter, open-label randomized phase III study, patients were randomly assigned to receive a MAC regimen that consisted of busulfan (16 mg/kg orally or 12.8 mg/kg intravenously total dose) and cyclophosphamide (120 mg/kg total dose) or a RIC regimen consisting of busulfan (8 mg/kg orally or 6.4 mg/kg intravenously total dose) and fludarabine (150 mg/m^2^ total dose) followed by allogeneic HCT from a related or unrelated (matched or 1 mismatch) donor. Detailed characteristics of patients and events (including events after 24 months) are listed in Table 1.

**Table 1:** Patients and outcome characteristics (*n* = 129).

Characteristic	Myeloablative Conditioning (MAC) *n* = 64	Reduced-Intensity Conditioning (RIC) *n* = 65	Total *n* (Δ from 24 months)	*p*
*Age, years*				0.87
Median (range)	50 (19–64)	51 (22–63)		
*Diagnosis according to WHO*				0.57
RAEB-1/RAEB-2/sAML/CMML	38	43	81	
Others (5q,RA,RARS,RCMD;RCMD-RS; unclassifiable)	24	22	46	
Missing	2		2	
*Prior induction chemotherapy*				0.25
No	33	40	74	
Yes	31	25	56	
*Donor*				0.96
Matched related	17	16	33	
Matched unrelated	36	38	74	
Mismatch unrelated/related	11	11	22	
*ATG as GVHD prophylaxis*				0.79
No	31	33	64	
Yes	33	32	65	
*Blasts at transplantation*				***0.03***
Median (range)	4% (0–18)	5% (0–18)		
>5%	18	30	48	
≤5%	46	35	81	
*Gender mismatch*				0.28
Male recipient/female donor	9	14	23	
Others	53	51	104	
Missing	2		2	
*IPSS at diagnosis*				0.74
Low risk/intermediate	30	25	55	
Intermediate II	18	24	42	
High risk	9	7	16	
Missing	7	7	14	
*Cytogenetic risk*				0.65
Low	24	28	52	
Intermediate	17	13	30	
High	17	18	35	
Missing	6	6	12	
*ECOG performance status at diagnosis*				0.69
0	18	21	39	
>0	35	34	69	
Missing	11	10	21	
*Busulfan*				>0.05
Intravenously	47	38	85	
Orally	16	27	43	
Missing	1	0	1	
*Stem cell source*				0.31
Bone marrow	3	6	9	
PBSC	61	59	120	
Missing	1	0	1	
*Outcome* *n (Δ from 24 months evaluation)*				
Alive	33 (−8)	40 (−10)	73 (−18)	
Alive without relapse	32 (−6)	33 (−8)	65 (−14)	
Alive after first relapse	1 (−2)	7 (−2)	8 (−4)	
Death	31 (+8)	25 (+10)	56 (+18)	
Non relapse death	19 (+2)	17 (+4)	36 (+6)	
Relapse	13 (+4)	15 (+4)	28 (+8)	
Death after first relapse	12 (+6)	8 (+6)	20 (+12)	
Second HCT	8	10	18	
Median time to second HCT; months			5.8 (1.2–96)	

*ATG* antilymphocyte globulin, *CMML* chronic myelomonocytic leukemia, *CMV* cytomegalovirus, *ECOG* Eastern Cooperative Oncology Group, *GVHD* graft-versus-host disease, *IPSS* International Prognostic Scoring System, *MDS* myelodysplastic syndrome, *PBSC* peripheral blood stem cell, *RA* refractory anemia, *RAEB* refractory anemia with excess of blasts, *RARS* refractory anemia with ringsideroblasts, *RCMD* refractory anemia with multilineage dysplasia, *sAML* secondary acute myeloid leukemia.

Significant variables are listed in bold.

Inclusion criteria were reported previously [3]. Recruitment started in 2004 and finished in 2012. Major inclusion criteria were cytologically proven MDS and sAML with <20% of blasts at HCT, a matched or one mismatch related or unrelated donor, age 18–60 years (amended to 65 in February 2006) for unrelated donors and age 50–65 years for related donors. Eighty-five percent of chemotherapies before transplantation were administered in advanced MDS (chronic myelomonocytic leukemia, refractory anemia with excess of blasts, and sAML) to reduce the number of blasts, whereas only 15% of chemotherapies were administered to less advanced MDS (refractory anemia, refractory anemia with ringsideroblasts, and refractory anemia with multilineage dysplasia). Other inclusion criteria were adequate hepatic, renal, pulmonary, and cardiac functions. Graft-versus-host disease (GVHD) prophylaxis consisted of cyclosporine and a short course of methotrexate (10 mg/m2 on days +1, +3, +6, and +11) for both arms. In the case of an unrelated donor, antilymphocyte globulin (Fresenius, Graefelfing, Germany) at a cumulative dose of 30 to 60 mg/kg or antithymocyte globulin (Thymoglobulin; Sanofi, Paris, France) at a cumulative dose of 6–10 mg/kg was administered divided on days −3, −2, and −1 according to center policy.

The primary endpoint for the long-term follow-up was overall survival (OS), and secondary endpoints were relapse-free survival (RFS), relapse incidence (RI), non-relapse mortality (NRM), and chronic graft-versus-host disease (GVHD) incidence. Furthermore, OS and RFS were analyzed in MDS subpopulations. Chronic GVHD was scored according to Shulman criteria (limited and extensive) [4]. For Random Assignment Procedure, patient enrollment, center and country distribution, definitions of graft failure, engraftment, and acute and chronic GVHD, see previous publication [5].

The RICMAC Study was conducted in accordance with good clinical practice guidelines and the provisions of the Declaration of Helsinki. Protocol approval was obtained from an independent ethics committee at each study site. All patients provided written informed consent. EBMT sponsored the study.

### Statistical methods

The main analysis and the landmark (conditional) analysis compared patients according to the randomization arm (Intent-To-Treat principle). In a secondary analysis, we excluded cases (*n* = 4) with major violations (Per-Protocol analysis). The landmark analysis included all patients alive relapse-free (and cGVHD-free for that endpoint) at 12 months post-allo. Table 1 describes differences in characteristics at baseline, reporting Fisher’s exact and Mann–Whitney tests *p*-values. OS and RFS were estimated by the Kaplan–Meier method and compared by Log-Rank test in univariable analysis and by Cox regression in multivariable analysis. NRM and relapse were analyzed as mutually competing risks. For Chronic GVHD, relapse or death were considered competing events, and onset was measured from 100 days post-transplant. For endpoints with competing events, we used the proper cumulative incidence estimator, the Gray test for univariable analysis, and Cox regression for the analysis of cause-specific hazards. Stratification factors (donor type, blasts, age), as well as patient and donor gender or gender mismatch, CMV status combination, diagnosis subgroup (sAML vs. other), cytogenetics, International Prognostic Scoring System score, performance status, prior chemotherapy, and use of busulfan (RIC vs. MAC), were considered for inclusion in Cox models with the random assignment arm. Selection was done on the basis of significance, taking into account prior clinical knowledge, the presence of missing values, and aspects related to model validation. Analyses were performed by using SPSS version 25 and R package version 3.3.

## Results

### Overall survival

**Fig. 1:**
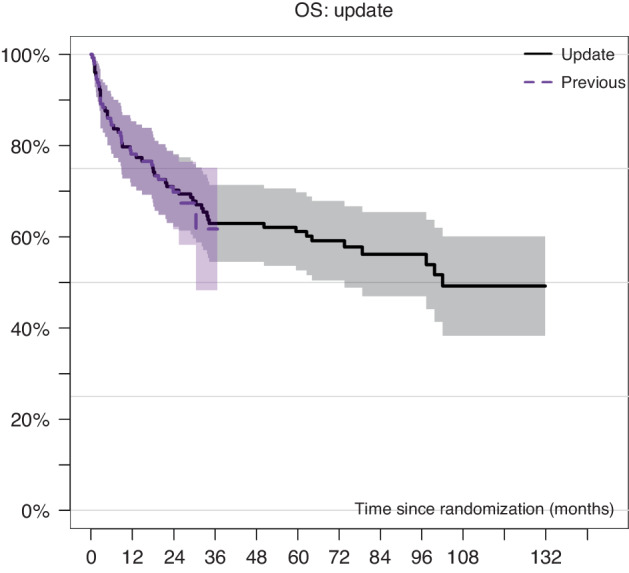
Initial analyses [5] versus updated analyses of overall survival of all patients (*n* = 129) recruited in the RICMAC study.

**Fig. 2:**
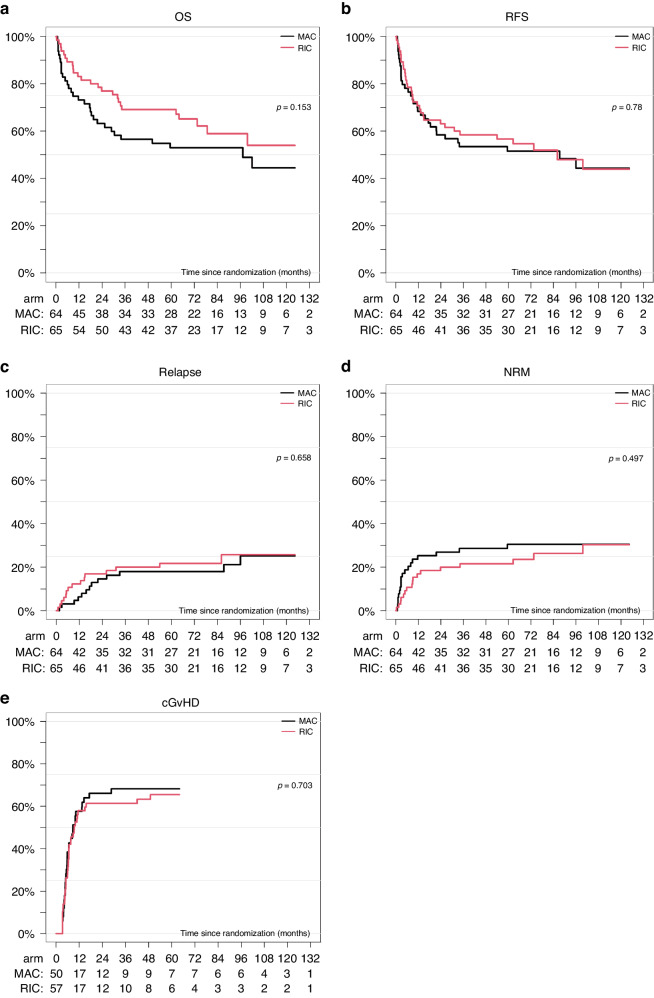
Outcome after MAC vs RIC in MDS/sAML. Overall survival (**a**), relapse-free survival (**b**), relapse incidence (**c**), non-relapse mortality (**d**), and chronic GVHD incidence (**e**) according to treatment arm [myeloalative (MAC) versus reduced intensity conditioning (RIC)] from transplant.

Comparing estimated OS from the initial analysis [5] with the current/updated analysis OS is presented in Fig. 1. The curves are overlapping during the first 24 months and now reach an OS of 50% at 10 years with a follow-up extended from a median follow up of 24 months to 74.9 (4.31–149.6) months (MAC 75.4 vs. RIC 72.2 months; *p* = 0.8). Median OS time was not reached (CI 95%: 73.6–) months for RIC and 97.3 (CI 95%: 25.4–) months for MAC. There was a trend towards improved OS 54.0 (CI 95%: 38.5–69.4)% of RIC in comparison to MAC 44.4 (CI 95%: 29.3–59.5)% at 10 years (Fig. 2a) without reaching statistical significance. The difference did not reach the 5% level of statistical significance (*p* = 0.15), and it was more evident in the first 100 days post-allo (HR = 0.31, *p* = 0.084). A total of 18 patients received a second allogeneic HCT, 8 in the MAC and 10 in the RIC group (Table 1). Interestingly, indications for 2nd HCT were 3 relapses, 4 graft failures, and one poor graft function in the MAC and 8 relapses, one graft failure, and one donor-cell derived MDS in the RIC group. For patients who underwent a second HSCT for relapse, median times to relapse and to transplant were 5.3 (1.0–33.1) and 8.5 (4.6–53.1) months, respectively. In the multivariate analysis, ECOG > 0 and the administration of chemotherapy were associated with OS (HR 2.45 and 2.31, respectively) with *p* = 0.01 each (Table 2). Time from diagnosis to transplant did not impact the outcome in both arms.

**Table 2:** Multivariate Cox regression analysis of risk factors for overall survival (OS), relapse-free survival (RFS), non-relapse mortality (NRM) and relapse incidence (RI).

Variable	OS		RFS		RI		NRM	
	HR (95% CI)	*p*-Value	HR (95% CI)	*p*-Value	HR (95% CI)	*p*-Value	HR (95% CI)	*p*-Value
RIC	0.64 (0.35–1.18)	0.15	0.93 (0.53–1.63)	0.80	1.48 (0.69–3.21)	0.32	0.74 (0.35–1.56)	0.42
Low cytogenetic risk	1.80 (0.80–4.02)	0.15	1.58 (0.74–3.35)	0.23	0.58 (0.21–1.61)	0.3	**4.14 (1.20–14.29)**	**0.02**
High cytogenetic risk	1.81 (0.82–3.99)	0.14	1.93 (0.92–4.04)	0.08	1.66 (0.69–3.98)	0.26	3.21 (0.87–11.87)	0.08
ECOG > 0	**2.45 (1.24–4.84)**	**0.01**	**2.08 (1.12–3.84)**	**0.02**	n.a.		**3.49 (1.33–9.16)**	**0.01**
Chemotherapy pre HCT	**2.31 (1.26–4.24)**	**0.01**	**2.00 (1.14–3.49)**	**0.01**	**3.02 (1.37–6.64)**	**0.006**		
Subgroup analysis								
Low cytogenetic risk: RIC	**0.22 (0.09–0.57)**	**0.002**	**0.37 (0.16–0.86)**	**0.02**			**0.29 (0.1**–**0.79)**	**0.02**
Intermediated cytogenetic risk: RIC	0.9 (0.25–3.23)	0.88	1.34 (0.4–4.44)	0.63			2.49 (0.23–27.52)	0.46
High cytogenetic risk: RIC	1.7 (0.64–4.51)	0.29	2.08 (0.84–5.12)	0.11			2.86 (0.71–11.52)	0.14

*HR* hazard ratio, *CI* confidence interval.

Significant variables are listed in bold.

### Relapse free survival

RFS at 10 years is presented in Fig. 2b and amounts to 44.2 (CI 95%: 29.9–58.6)% for MAC and 43.9 (CI 95%: 29.1–58.8)% for RIC (*p* = 0.78). Median time of RFS totals 87.4 (CI 95%: 18.4–) for MAC and 86.1 (26.2–) months for RIC. Similar to OS, ECOG > 0 and chemotherapy was an independent determinant for RFS (HR 2.08; *p* = 0.02 and HR 2.00; *p* = 0.01, respectively; Table 2).

### RI

Cumulative Incidence of relapse at 10 years was 25.2% (CI 95%: 12.3–38.2) after MAC and 25.7% (CI 95%: 13.5–38.0) (*p* = 0.66) after RIC. Administration of chemotherapy prior to HSCT was associated with a higher RI (HR 3.02; CI 95%: 1.37–6.64; *p* = 0.006; Table 2).

### NRM and chronic GVHD

The non–relapse mortality was 30.5% (CI 95%:19.0-42.0) after MAC and 30.3% (CI 95%:17.2–43.5) after RIC at 10 years (*p* = 0.50). The only significant predictors for NRM in the multivariate analysis were the low cytogenetic risk group [HR: 4.14 (1.20–14.29) *p* = 0.02] and ECOG > 0 [HR 3.49 (1.33–9.16), *p* = 0.01]. A total of 70 patients developed cGVHD (limited *n* = 15, extensive *n* = 49, 6-grade unknown) by a median of 6.0 months (range, 3.3–48.9). Five patients had cGVHD following 2nd HCT because of relapse, 3 extensive, and 2 of unknown grade. Chronic GVHD incidence did not differ between the two groups and was 68.2% (CI 95%: 55.0–81.4) for MAC and 65.5% (CI 95%: 53.0–78.0) for RIC (*p* = 0.70). RIC had evidence for less NRM than MAC in the first 100 days post-HCT, though not reaching significance at the canonical 5% level (HR = 0.30, *p* = 0.075).

### Analysis of landmark 12 months and per protocol

**Fig. 3:**
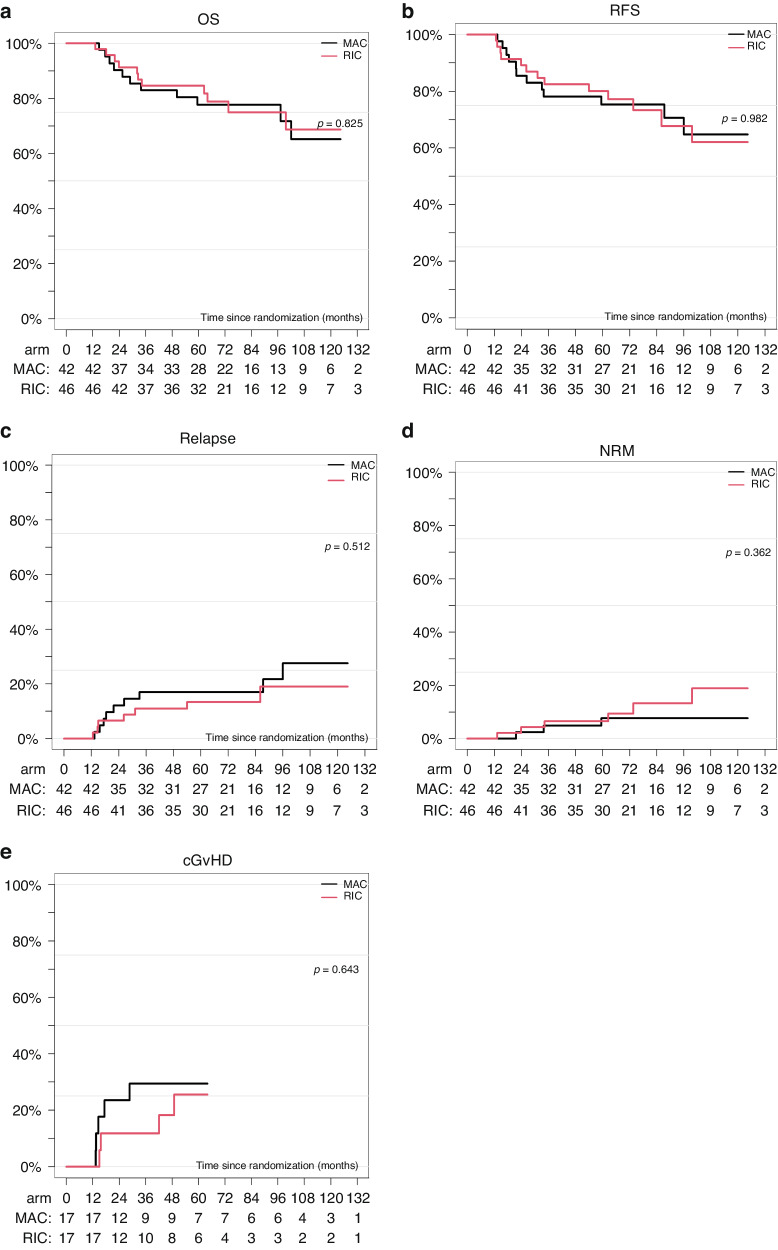
Outcome after MAC vs RIC in MDS/sAM at landmark 12 months. Overall survival (**a**), relapse-free survival (**b**), relapse incidence (**c**), non-relapse mortality (**d**), and chronic GVHD Incidence (**e**) according to treatment arm [myeloalative (MAC) versus reduced intensity conditioning (RIC)] as Landmark Analysis at 12 months.

Analyses of OS, RFS, NRM, RI, and incidence of cGVHD were performed as a landmark analysis at 12 months to show the effect without short-term influences of NRM within one year. The difference in OS described above was not seen in the landmark analysis (Fig. 3a), but was confirmed in the analysis per protocol (only 4 patients less in the per protocol analyses). It should be noted that RFS after RIC and MAC overlapped in all three analyses [final analysis (Fig. 2b), landmark (Fig. 3b), and per protocol (data not shown]. RI (Fig. 3c), NRM (Fig. 3d), and cGvHD (Fig. 3e) were without statistical difference.

### Subgroup analysis

We were interested in looking at particular subgroups of patients that might benefit from one of the two preparative regimens. Among patients with low cytogenetic risk profiles those who received RIC had better outcomes compared to MAC: OS (HR 0.22 (CI 95%: 0.09–0.57); *p* = 0.002), RFS [HR 0.37 (CI 95%: 0.16–0.86); *p* = 0.02] and lower NRM [0.29 (CI 95%: 0.1–0.79); *p* = 0.02] but with no difference in RI (Table 2).

## Discussion

In the present long-term follow-up of the multicenter, multinational, randomized phase III RIC-MAC study, we confirmed our previously published results and highlighted new findings. As of to date, the present analysis is based on the longest follow up in a randomized study for HCT in MDS. First of all, HCT leads to a long-term OS of 49% (95% CI: 38.3–60.1) at 10 years, irrespective of the preparative regimen and with similar results following RIC (FluBu2) and MAC (BuCy). Overall RI was around 25.4% (16.5–34.3) and NRM 30.4% (21.6–39.1) at 10 years without statistically significant differences between the two arms. HCT after RIC and MAC display comparable long-term outcomes in patients up to 65 years with MDS. RIC showed a trend for better OS as compared to MAC early after HCT but RFS was overlapping. In general, RIC protocols have been shown to have less morbidity compared to MAC, possibly due to a lower infection rate [6] and faster engraftment with a lower likelihood of bleeding complications [7] and a higher probability of pregnancy [8, 9], but also to less stomatitis and to a trend to overall lower Bearmann grade >1 toxicity in the randomized RICMAC [3] study. Chronic GVHD did not differ between the two arms. Risk factors in a multivariate analysis were ECOG > 0 (OS, RFS, NRM, and RI) and chemotherapy prior to HCT (OS, RFS, and RI). Low-risk cytogenetics was an independent risk factor for OS, RFS, and NRM. Similarly, previous chemotherapy negatively influenced RI, RFS, and OS. This finding is not easily explainable and might represent a selection of high-risk patients needing chemotherapy before HCT or the selection of resistant MDS clones during pre-transplant therapy [10].

RI in our study was lower than published in other studies with 37% for MDS and 51% for AML patients with RIC [11]. NRM was higher than described and could be explained by the earlier recruitment period, where no high-resolution typing was available. There is no data on OS at 10 years available for comparison, but OS compares favorably to 70.2% (95% CI: 51.8–85.7%) for MAC vs. 58.3% (95% CI: 39.2–76.2%) for RIC at 4 years.

Randomized comparisons between MAC and RIC of the BMT–CTN trial were published previously [11, 12]. Here, at 4 years, the NRM was 25.1% for MAC, compared with 9.9% for RIC (*p* < 0.001), but patients who received RIC had a significantly higher risk of relapse (60.7% vs. 19.8%; [HR], 4.06; 95% CI: 2.59–6.35; *p* < 0.001). OS was superior for patients who received MAC compared to those who received RIC (HR, 1.54; 95% CI: 1.07–2.2; *p* = 0.03), but OS in only MDS patients was 70.2% (95% CI: 51.8–85.7%) for MAC versus 58.3% (95% CI: 39.2–76.2%) for RIC and not statistically significantly different (*p* = 0.366). There are several differences between this analysis and our clinical trial. Scott et al. analyzed patients with AML (*n* = 218) and MDS (*n* = 54) in contrast to 125 patients with MDS (including 12 patients with AML) in our study. Furthermore, different conditioning regimens were allowed (RIC: FLU + BU2 or Melphalan; MAC: BU4/CY or FLU+Bu4 or Cy+TBI 12 or 14.2 Gy), and a variety of GVHD regimens and ATG were allowed as decided by each center. In our trial, uniform conditioning of BU4/CY for MAC and FLU/BU2 for RIC and the same GVHD prophylaxis was used. Median follow-up was 75 vs. 51 months. In agreement with the Scott study, OS (primary endpoint in both studies) and RFS of MDS patients were not different in the two studies.

A metaanalysis looking at 31 clinical trials on MDS patients, identified only 2 prospective randomized trials of RIC versus MAC reporting OS, RFS, NRM, relapse, and GVHD [13]. Combined analysis of MDS patients revealed no difference in OS, RFS, and RI.

Our results also compares favorably to the OS and RFS described in another randomized study using busulfan based RIC versus treosulfan [14, 15]. The estimated OS at 36 months were 52.4 (42.2–61.6) for RIC and 62.5 (48.4–73.7) for treosulfan.

The lack of an IPSS-R score, which was not available at the study start may be considered a limitation of the study. The study shows that in younger patients with a median age of 51 years, the OS and RFS did not differ significantly between RIC and MAC. The results presented here refer only to the comparison of FluBu2 as RIC versus BuCy as MAC and not for other RIC (e.g., FluMel) or MAC regimens.

Overall, we report that low risk cytogenetic patients have the highest benefit with RIC for OS and RFS because of lower NRM. Therefore RIC may be considered as equivalent to MAC in younger patients with MDS, but preferentially cytogenetic low risk patients should be treated with RIC.

### Supplementary information

Study Protocol

## Data Availability

Data can be requested by e-mail to the corresponding author.
