# Supplementary material for: Reduced intensity versus myeloablative conditioning for MDS: long-term results of an EBMT phase III study (RICMAC)
Source: Bone Marrow Transplant. 2024 Apr 25;59(8):1084–91. doi: 10.1038/s41409-024-02282-7 (PMC11296945; doi:10.1038/s41409-024-02282-7)
Supplement: Supplementary file 1 — Study Protocol [file 41409_2024_2282_MOESM1_ESM.pdf]

This document contains the last version of protocol:

Dose-reduced versus standard conditioning followed by allogeneic stem cell transplantation in patients with MDS or sAML: A randomised phase III study (RICMAC)

Version	Date	Changes
1	10-03-2004	Original protocol
2	15-02-2006	Amendment 1: Age inclusion criteria extended
3	07-08-2006	Amendment 2: Bringing up to EU standards
4	15-08-2007	Amendment 3: Age inclusion criteria extended in Germany
5	18-01-2008	Amendment 3: Age inclusion criteria extended EBMT wide

Changes to this protocol will be official and valid only after the protocol has been amended in writing, approved by the relevant authorities, and signed by the persons named below.

On behalf of the EBMT (sponsor)

Coordinating Investigator

Prof. Dr. N. Kröger

Working Party Chairperson

Prof. Dr. T. M. de Witte

INVESTIGATOR'S AGREEMENT

My signature, below, confirms that I have read this protocol and that I understand my responsibilities under the protocol and that I will conduct my functions and responsibilities in this clinical trial in accordance with this version of the protocol, national regulations and the principles of Good Clinical Practice.

Principal Investigator at Site ____________________________________

Prof / Dr: ____________________________________

Name Hospital: ____________________________________

Date: …... / …... / …………..

Please fax a copy of this page to:

Chronic Leukaemia Working Party Data Office: **Fax: +49 180 500 290 623**

The Chronic Leukaemia Working Party

MDS-Subcommittee

Dose-reduced versus standard conditioning followed by allogeneic stem cell transplantation in patients with MDS or sAML: A randomised phase III study

**Sponsor**	European Group for Blood and Marrow Transplantation (EBMT)
**Sponsor ID of the Study**	42205525
**EudraCT number**	2005-002011-24
**Coordinating Investigators**	***Nicolaus Kröger***<br>Bone Marrow Transplantation<br>University Hospital Hamburg-Eppendorf<br>Martinistraße 52, 20246 Hamburg / Germany<br>Phone +49-40-42803-4850/-4851<br>Fax +49-40-42803-3795<br>e-mail: nkroeger@uke.uni-hamburg.de<br>***Theo de Witte, University Hospital Nijmegen, The Netherlands***<br>(Chairman "CLWP")
**Scientific Committee**	Theo de Witte, University Hospital Nijmegen, The Netherlands<br>Axel R. Zander, BMT, University Hospital Hamburg-Eppendorf, Germany<br>Arnon Nagler, The Chaim Sheba Medical Center, Tel Hashomer, Israel<br>Ghulam J. Mufti, King's College Hospital, London, United Kingdom<br>Rodrigo Martino, Hospital de la Santa Creu I Sant Pau, Barcelona, Spain<br>Philippe Guardiola, CHRU, Angers, France<br>Hartmut Biersack, University Hospital Essen, Germany<br>Christoph Schmid, Klinikum Augsburg, Germany<br>Kathrin Haifa Al-Ali, University Hospital Leipzig, Germany<br>Dietger Niederwieser, University Hospital Leipzig, Germany<br>Alessandro Rambaldi, Ospedale Bergamo, Italy<br>Bernd Hertenstein, Klinikum Bremen - Mitte, Germany<br>Hermann Einsele, Medizinische und Poliklinik II Würzburg, Germany<br>Paolo Corradini, Istituto Nazionale Tumori, Milano, Italy<br>Francesco Onida, IRCCS Ospedale Maggiore of Milan, Italy<br>Alois Gratwohl, University Hospital Basel, Switzerland
**Statistician**	Ronald Brand, Dept. of Medical Statistics, LUMC, Leiden, The Netherlands
**Data Manager**	Marleen van Os, CLWP Data Management Office, Leiden, The Netherlands

CONTENTS PAGE

1 BACKGROUND 8

2 AIMS OF STUDY 10

3 STUDY ENDPOINTS 10
3.1 Primary endpoint 10
3.2 Secondary Endpoints 10

4 STUDY DESIGN 11

5 STUDY DURATION 11
5.1 Start of study 11
5.2 Accrual of the patients 11

6 STATISTICAL CONSIDERATIONS 11

7 PATIENTS' SELECTION 13
7.1 Inclusion criteria 13
7.2 Exclusion criteria 14

8 DONOR SELECTION 15

9 SERIAL MEASUREMENTS AND STUDY CALENDAR 15

10 REGISTRATION 15

11 STRATIFICATION 18
11.1 Plan for interim analyses 19

12 RANDOMISATION 19

13 GRAFT SOURCE 19

14 TREATMENT PLAN 20
14.1 Randomisation 20
14.1.1 Arm A (standard conditioning) 20
14.1.1.1 Busulfan 20
14.1.1.2 Cyclophosphamide 20
14.1.1.3 Mesna 21
14.1.2 Arm B (reduced conditioning) 21
14.1.2.1 Busulfan 21
14.1.2.2 Fludarabine 21
14.1.2.3 Mesna 21
14.2 Flow sheet 22
14.3 GvHD-Prophylaxis 23
14.3.1 … in case of *related* donor 23
14.3.2 … in case of *unrelated* donor 23
14.4 Growth factors (optional) 26
14.5 Infection prophylaxis 26

**14.6**	**Blood products**	**26**
**14.7**	**Supportive care**	**27**
**14.8**	**Chimerism studies**	**27**
**15**	**ELIGIBILITY CRITERIA FOR DONOR LYMPHOCYTE INFUSION (DLI)**	**28**
**15.1**	**Mixed chimerism or if T-cell chimerism decreases**	**28**
**15.2**	**Progression or symptomatic relapse after complete haematological remission**	**28**
**16**	**END OF STUDY**	**29**
**16.1**	**... for the patient**	**29**
**16.2**	**... for the whole study**	**29**
**17**	**GVHD-SCORE**	**29**
**17.1**	**Acute GvHD**	**29**
**17.2**	**Chronic GvHD**	**29**
**18**	**EVALUATION OF TOXICITY**	**30**
**19**	**REPORTING OF ADVERSE EVENT (AE) / SERIOUS ADVERSE EVENT (SAE)**	**30**
**19.1**	**Definitions:**	**30**
19.1.1	Adverse Event (AE)	30
19.1.2	Serious Adverse Event (SAE)	31
19.1.3	Adverse Drug Reaction (ADR)	31
19.1.4	Serious Adverse Drug Reaction (SADR)	31
19.1.5	Suspected Unexpected Serious Adverse (Drug) Reaction (SUSAR)	31
**19.2**	**Recording, Reporting and Reviewing**	**32**
19.2.1	Reporting of Adverse Events (AE)	32
19.2.2	Reporting of Serious Adverse Events (SAE)	32
**19.3**	**Assessment of causality**	**33**
**20**	**EVALUATION AFTER TRANSPLANTATION**	**34**
**20.1**	**Criteria to define complete remission**	**34**
**20.2**	**Criteria to define relapse incidence**	**35**
**20.3**	**Criteria to define Event-free survival**	**35**
**20.4**	**Criteria to define overall survival**	**35**
**21**	**ENGRAFTMENT FAILURE**	**35**
**22**	**DATA RECORDING, MANAGEMENT AND MONITORING - FORMS AND PROCEDURES FOR COLLECTING DATA**	**37**
**22.1**	**Patient Enrolment**	**37**
**22.2**	**Regulatory and ethical obligations**	**37**
22.2.1	Independent Ethics Committee / Institutional Review Board	38
**22.3**	**Documentation requirements pre start of trial**	**39**
**22.4**	**Data monitoring committee**	**40**

22.5 Monitoring 40
22.5.1 Initiation visits 40
22.5.2 Close-out visits 40
22.6 Schedule to complete report forms 40
22.6.1 After fulfilling the inclusion criteria 40
22.6.2 During and after treatment 41
22.7 Record retention requirements 41

23 ETHICAL CONSIDERATIONS 41
23.1 Patients' protection 41
23.2 Patient Confidentiality / Subject identification 42
23.2.1 Informed consent 42
23.3 Insurance 43

24 PUBLICATION POLICY 43

25 ADMINISTRATIVE AND LEGAL OBLIGATIONS 44
25.1 Trial Documentation and Storage 44

26 LITERATURE 45

27 APPENDICES 47
27.1 Serial Measurements and study calendar 48
27.2 Risk-Score acc. to the International MDS Workshop (IPSS) 49
27.3 FAB Classification of myelodysplastic syndromes (acc. to Bennett) 50
27.3.1 Refractory anaemia (RA) 50
27.3.2 Refractory anaemia (RA) with ring sideroblasts (RARS) 50
27.3.3 Refractory anaemia (RA)with excess of blasts (RAEB) 50
27.3.4 RAEB in transformation (RAEB-t) 51
27.3.5 Chronic myelomonocytic leukaemia (CMMoL) 51
27.4 Myelodysplastic Syndrome Classification 2000 (WHO) 52
27.5 Organ-related toxicity of conditioning acc. to Bearman et al. 53
27.6 Acute GvHD-Staging (Glucksberg et al., 1974) 55
27.7 Acute GvHD-Grading (Glucksberg et al., 1974) 56
27.8 Chronic GvHD-Grading (acc. to Shulman) 57
27.9 VOD-Criteria and -Grading (McDonald et al.) 58
27.10 ECOG-Performance Status Scale 59
27.11 Drug information 60
27.11.1 Busulfan 60
27.11.2 Busilvex 62
27.11.3 Cyclophosphamide 70
27.11.4 Cyclosporine 74
27.11.5 Fludarabine 80
27.11.6 Mesna 82
27.12 Patient's Information / Written Consent (German Version)
Patienteninformation und –einverständniserklärung 83
27.12.1 Patienteninformation 84

27.12.1.1 Obliegenheiten des Versicherten (Auszug aus den Allgemeinen Versicherungsbedingungen für klinische Prüfungen von Arzneimitteln (AVB)) 93
27.12.1.1.1 § 14 II (1) – (6): Obliegenheiten des Versicherten 93
27.12.1.2 Datenschutzbestimmungen gemäß § 40 (1) 2. AMG 95
27.12.2 Patienteneinverständniserklärung 97
27.13 Patient's written informed consent (English version) 98
27.13.1 Information for the patient 98
27.13.2 Patient's written informed consent 111
27.14 Participating Centers 113
27.15 Country coordinators 114
27.16 Documentation Sheets 115

1 BACKGROUND

Myelodysplastic syndromes (MDS) are a heterogeneous group of clonal haematological disorders, which are characterised by abnormal cellular maturation resulting in cytopenias and a variable risk of progression to acute leukaemia [1]. Patients have traditionally been classified into groups according to the 1982 French-American-British Consensus (FAB) classification (see Appendix 26.3 on page 50) or on the percentage of bone marrow blasts [2, 3]. Recently, a new classification proposal by the WHO has been introduced (see Appendix 26.4 on page 52). A further prognostic tool has been proposed by the International Prognostic Scoring System (IPSS) [4] (see Appendix 26.2 on page 49). The median survival for patients with MDS ranged from several months to several years, and prognosis depends on several factors:

- The marrow blast percentage,
- the karyotype of the clone,
- and the numbers of cytopenias at diagnosis.

For low-risk patients with only few blasts and a normal karyotype and only one or no cytopenia a median survival of five to six years has been described. For those patients with higher risk features the estimated overall survival ranged between four and fourteen months. While advances are being made in the biology of MDS which will hopefully allow for more effective therapies in the future, currently allogeneic transplantation has been shown the most effective treatment. With induction chemotherapy using acute myeloid leukaemia-protocols a proportion of patients achieved complete remissions, but these have been durable only in 15 - 20 percent of the cases [5]. For patients without an HLA-matched allogeneic donor autologous stem cell transplantation may be used as consolidation therapy after successful conventional induction chemotherapy. The survival rates after allogeneic stem cell transplantation resulted in different studies between 13 percent and 75 percent. The difference in survival is due to different patient populations having different prognosis such as de novo-MDS, treatment-induced MDS (T-MDS), AML arising from MDS, treatment-induced AML and de novo-AML. These categories of patients can be quite different in their biologies, natural history and response to treatment. A larger series has been reported from the European Group for Blood and Marrow Transplantation (EBMT) [6]. They report a disease-free survival at three years of 36 percent and a non-relapse mortality of 37 percent in the 885 patients who had an HLA-identical sibling donor. Disease-free survival and relapse rates were 55 percent

and 13 percent, respectively, in patients with RA/RARS, while corresponding figures for more advance disease were 28 percent and 43 percent. The Seattle experience on 251 MDS patients reported a 40 percent disease-free survival at six years with an 18 percent relapse rate. In that group age as well as the IPSS were highly predictive for disease-free survival. So patients younger than 20 years of age had a disease-free survival of 60 percent compared to only 20 percent for those patients older than 50 years of age which was mainly due to the high treatment-related mortality in the older age group. However, more than two-third of the patients with MDS are older than 60 years of age. There is particular interest in the use of stem cell transplantation in older patients. However, due to the high treatment-related mortality induced by conventional standard conditioning regimens, new therapies to lower the treatment-related mortality are urgently needed. A trial from the Seattle group reported an overall survival of 46 percent and a disease-free survival of 42 percent at three years with 50 patients being 55 - 60 years of age after transplantation with targeted dose busulfan [7, 8]. Recently, in order to exploit the graft-versus-leukaemia or the graft-versus-MDS effect, reduced intensity allogeneic stem cell transplantation has been developed for patients with haematological malignancies. Substances commonly used in the so-called non-myeloablative or reduced intensity conditioning regimens are fludarabine, cytosine, low-dose total body irradiation or busulfan. Parker et al. using fludarabine (150 mg/m² body surface), busulfan (8 mg/kg body weight), Campath-1A (100 mg) reported in 23 patients a treatment-related mortality of 9 percent, an overall survival at two years of 48 percent, and a disease-free survival at two years of 39 percent [9]. Martino et al. reported on 20 patients with MDS who underwent allogeneic peripheral stem cell transplantation from a HLA-identical sibling after dose reduced conditioning consisting of busulfan and fludarabine. He reported a probability of transplant related mortality at one year of only 5 percent and a progression free survival at one year of 66 percent [10]. The German Cooperative Transplant Study Group reported on 37 patients with myelodysplastic syndromes or secondary acute leukaemia that underwent stem cell transplantation from related and unrelated donors after a dose reduced conditioning regimen consisting of fludarabine and busulfan (8 mg/kg body weight). They reported a treatment-related mortality of 27 percent which was higher after unrelated than after related transplantation (45 percent vs. 12 percent; p=0.03) [11].

2 AIMS OF STUDY

The present study will be a multicenter, prospective phase III-study comparing dose-reduced versus standard conditioning followed by allogeneic stem cell transplantation from related or unrelated donors in patients with MDS or secondary AML.

3 STUDY ENDPOINTS

3.1 PRIMARY ENDPOINT

The hypothesis is that a dose-reduced conditioning will reduce the non-relapse mortality from 40 % to 20 % at one year after allogeneic stem cell transplantation.

3.2 SECONDARY ENDPOINTS

- Comparison of haematopoietic recovery by day +30 post transplant between two arms.
- Comparison of toxicity of both regimens according to the Bearman-Score [12] (Appendix 26.5 on page 53).
- Incidence of acute graft-versus-host disease by day +100 post-transplant acc. to the Glucksberg scale [13] (Appendix 26.6 on page 55 and Appendix 26.7 on page 56)
- Incidence of chronic graft-versus-host disease ("limited" or "extensive") by day +365 post-transplant as per to the criteria according to Shulman [14] (Appendix 26.8 on page 57).
- Comparison of overall survival post-transplant at two years.
- Comparison of event-free survival post-transplant at two years.
- Cumulative incidence of relapse post-transplant at two years between both groups.
- Comparison of VOD between the two arms (staging and grading according to the McDonald-scale [15] (Appendix 26.9 on page 58).
- Comparison of incidence of bacterial, viral, fungal and protozoal infection at day 100, at one year and at two years after transplantation.

4 STUDY DESIGN

Prospective randomised phase III multi-center study.

5 STUDY DURATION

5.1 START OF STUDY

05/2004.

5.2 ACCRUAL OF THE PATIENTS

3 years.

6 STATISTICAL CONSIDERATIONS

The study is designed to be able to detect a difference in one-year Treatment-Related Mortality of 40 percent versus 20 percent with a power of 90 percent using a Proportional Hazards model with the usual alpha of 5 percent. The power computation is based on the following assumptions: Accrual for three years in a uniform way (50 percent accrued after 1.5 years); analysis one year after the last patient has been included; a lost-to-follow-up percentage of 10 percent in each arm; two-sided significance test for the Hazard Ratio. The total number of patients needed is depicted in Graph 1.

Hence we need a total of 160 patients to achieve this goal. If the analysis were to be done immediately after the last patient entered the trial (instead of after one year) one would need to have entered 200 patients to achieve the same power for the same difference.

The data will be analyzed with a Cox Proportional Hazards model. A test for non-proportionality will be performed. In case of serious departure from the proportional hazards assumption (e.g. when we find crossing survival curves), the analysis will restrict to the one-year estimate only instead of an overall log-rank test. To enhance power of the comparison, we will adjust in the final COX model for the major stratification variables or any risk factors known to be associated with TRM and available to reduce the error. Possible interactions of these major risk factors with the randomisation variable ("dose-reduced yes or no") will be taken into account and included in the final analysis if their significance level is below 0.10. In case of such interactions being significant, an adjusted Hazard Ratio will still be computed provided

the Hazard Ratio's in the strata induced by the significant interactions are on the same side of the null-hypothesis (HR=1.0), so if all strata show either a favourable or unfavourable effect. In case all strata show significant but opposite effects, we will refrain from publishing an overall effect of the randomisation factor but state separate effects for each of the strata.

Graph 1 Number of patients needed

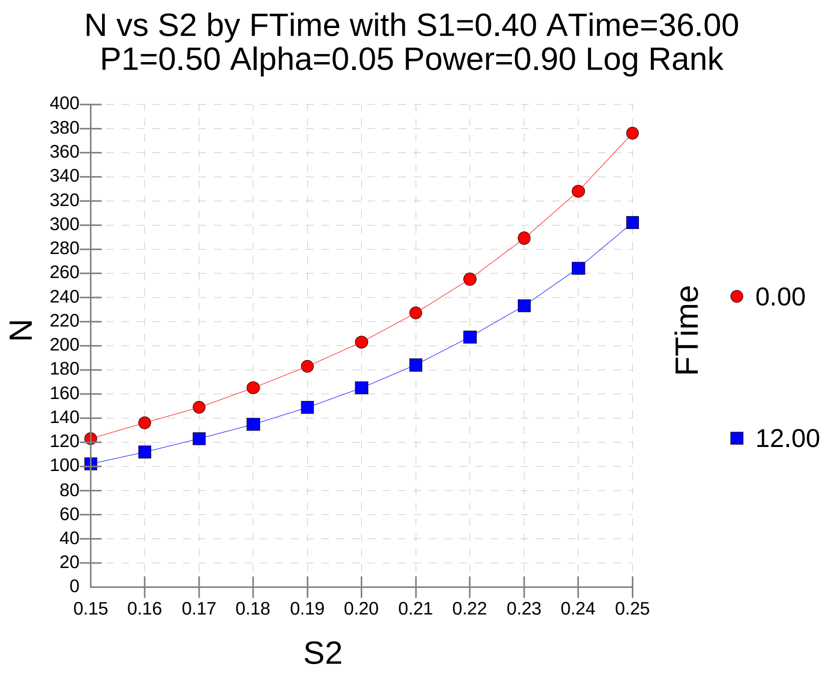

7 PATIENTS' SELECTION

7.1 INCLUSION CRITERIA

- Disease: Cytologically proven
  - primary or therapy-related myelodysplastic syndrome (MDS), either as
    - refractory anaemia (RA) according FAB or RA with or without dysplasia according WHO,
    - refractory anaemia with ringsideroblasts (RARS) according FAB or RARS with or without dysplasia according WHO,
    - refractory anaemia with excess of blasts (RAEB) according FAB or RAEB I or RAEB II according WHO,
    - refractory anaemia with excess of blast in transformation (RAEB-T) according FAB,
    - CMML (dysplastic type) according WHO,
  - or secondary acute myeloid leukaemia (sAML).
- Blast count < 20 percent in bone marrow with or without chemotherapy at time of transplantation.
- Patient eligible for standard and dose-reduced conditioning as per local guideline.
- **Patient age 18 – 60 years** if donor is a HLA-matched unrelated donor (HLA-A, HLA-B, HLA-DRB1 and HLA-DQB1) (one mismatch allowed):
- **Patient age 18 – 65 years** if donor is a HLA-matched related donor ((HLA-A, HLA-B, HLA-DRB1 and HLA-DQB1) (one antigen-mismatch allowed):
- No major organ dysfunction.
- Written informed consent of the patient.

7.2 EXCLUSION CRITERIA

- Blasts > 20 % in bone marrow at time of transplantation
- No written informed consent.
- Central nervous involvement.
- Severe irreversible renal, hepatic, pulmonary or cardiac disease, such as
  - Total bilirubin, SGPT or SGOT > 2 times upper the normal level.
  - Left ventricular ejection fraction < 30 %.
  - Creatinine clearance < 30 ml/min.
  - DLCO < 35 % and/or receiving supplementary continuous oxygen.
- Positive serology for HIV.
- Pregnant or lactating women.
- Patients with a life-expectancy of less than six months because of another debilitating disease.
- Serious psychiatric or psychological disorders.
- Invasive fungal infection at time of registration.

8 DONOR SELECTION

- Donors should be matched for HLA-A and -B (serologically) and -DRB1 and DQB1 (by resolution typing) (one mismatch is allowed).
- Peripheral blood stem cells are the preferred stem cell graft; however, bone marrow cells are also acceptable.
- Peripheral blood stem cells should be mobilised with G-CSF according to the standard national protocol.
- Harvesting of peripheral blood stem cells is made by leukapheresis, usually starting on day +4 or +5 after initiation of G-CSF treatment.
- The bone marrow cells are harvested according to centre's policy.
- Donors should be asked before harvest whether they are willing to donate lymphocytes later by aphaeresis.

9 SERIAL MEASUREMENTS AND STUDY CALENDAR

See Appendix 26.1 on page 48.

10 REGISTRATION

On the day of registration the patient and the donor must meet all inclusion criteria and no exclusion criteria should be present (see sections 7 and 8 on page 13ff).

Signed written consents will be obtained from the patient using forms approved by the main / National Ethical Committee of the participating institution.

Before patients and donors can be entered in the study, and depending on the country, the study has to be approved in writing by some of or all the following authorities and committees:

- National Ethics Committee.
- (National) Competent Authority.
- Site specific Ethics Committee (i. e. local committees).
- Institutional review board.

Proof of appropriate authorization must be sent to either Ms Anja van Biezen or Ms Marleen van Os at the Randomisation and Data Management Office of the Chronic Leukaemia Working Party in Leiden, Netherlands. Without proof of appropriate authorization no patient should be entered into the study.

Each patient will then be registered and randomised at the Randomisation and Data Management Office of the CLWP (address given below), and a unique patient number (UPN) will be assigned to the patient. Patient's informed consents must be written signed and obtained at randomisation.

New countries joining the study will have to send the patient's information sheets to the main / National Ethics Committee for approval.

The participating center should announce the GvHD-prophylaxis strategy for all their included unrelated stem cell transplantations.

Each centre should register its patients using the Registration Form for this study available on the EBMT/CLWP/Clinical trial website

http://www.ebmt.org/5WorkingParties/CLWP/clwpct.html and fax it to:

CLWP Data Office
LUMC Postzone S-5-P
PO Box 9600
2300 RC Leiden
The Netherlands
Phone: +31-71-526-9722
Fax: +49-180-500-290-623
E-Mail: clwpebmt@lumc.nl

The eligibility is confirmed and before starting treatment, the office should be contacted with the following details:

- Protocol name
- Institution name
- Caller's name
- Responsible physician
- Patient's initials
- Patient's hospital number
- Patient's date of birth
- Patient's sex
- Eligibility criteria

The randomisation will take place at the CLWP Data Management Office which will send a copy of the randomisation confirmation by facsimile and by secure internet access to the site, notifying of the treatment allocations and confirming the patient's details which should be checked at the site for accuracy.

11 STRATIFICATION

The randomisation scheme will be a variable-block size balanced randomisation, with stratification for at least the participating center and for related versus unrelated.

When a center expresses the intention to contribute patients to this trial, a descriptive analysis of the EBMT registry data base takes place to count the number of patients of the same type as eligible in this study contributed to the data base by that center during a period comparable to the accrual time available to that center in the current study. Based on this number of patient contributed in the past, a stratification scheme is designed for this particular center. The reason for this center-dependent approach is the necessity to avoid too fine a stratification while maintaining an optimal balance since over-stratification may lead to data loss or (severe) bias.

The following stratification factors are available in order of importance:

Stratification factor	applicable if ….
related versus unrelated	always
blast count less than 5% versus 5% or more in bone marrow prior to transplantation	only if in *both* the “related” and “unrelated” strata, 4 or more patients are expected to participate
age < 45 years versus ≥ 45 years	only if in all 4 strata resulting from the 2 factors above, we expect at least 4 patients

No further stratification will be done to avoid over-stratification (in case of too fine strata). A randomisation list will be prepared for the center taking into account the expected numbers from the registry analysis and the above stratification algorithm. The list is available to the data manager performing the randomisation.

Other important risk factors will be adjusted for in the analysis if necessary to enhance the power of the comparison in the COX model. The stratification by center serves only the balancing of the randomisation group but will not be a factor in the final COX model; all other risk factors will be included in the model if either error-reduction is achieved by the inclusion or the interaction with the randomisation factor is significant at the 10 percent level.

IPSS, low / intermediate risk	vs.	IPSS, high risk
Unrelated donor	vs.	Related donor
Prior induction chemotherapy	vs.	No prior induction chemotherapy
Age at time of transplantation		(continuous)

11.1 PLAN FOR INTERIM ANALYSES

One interim analysis is planned, primarily to evaluate severe adverse effects, which could warn against the continuation of the trial. The interim analysis is planned after 120 patients have entered the study, including those patients who were randomized and included but subsequently excluded.. Before that, the failure rate and serious adverse event rate will be closely monitored in order to analyze unexpected trends. For this purpose, every six months, the Data Monitoring Committee will receive from the Leiden EBMT Data Monitoring and Randomisation Office a summary of the current state of the protocol with a list of all adverse events that occurred.

12 RANDOMISATION

Administration of randomisation is separated from the clinical investigators.

The randomisation will be arranged by the study office of the CLWP in Leiden (see section 10ff).

13 GRAFT SOURCE

Peripheral blood stem cells are preferred (at least ≥ 3 x 10^6 $CD34^+$-cells per kilogram recipient's body weight (bone marrow is allowed).

14 TREATMENT PLAN

14.1 RANDOMISATION

The patient will be randomised between a dose reduced conditioning (Arm B) and a standard conditioning (Arm A).

14.1.1 ARM A (STANDARD CONDITIONING)

The standard conditioning (Arm A) consisting of busulfan and cyclophosphamide. The interval between the last dose of Busilvex® or busulfan and cyclophosphamide should be 24 hours.

14.1.1.1 BUSULFAN

Busulfan will be given at a total dose of 16 mg/kg BW orally or 12.8 mg/kg intravenously and it will be administered from day -9 to day -6 at a dose of 4 mg/kg BW (orally) or 3.2 mg/kg BW (intravenously) per day. The preferred application of busulfan, however, will be Busilvex® intravenously. Busilvex® should be administered intravenously via a central venous catheter. The dose of Busilvex® will be 0.8 mg/kg of ideal body weight or actual body weight, whichever is lower, administered as a two hour infusion every six hours for four days for a total of 16 doses, starting on day -9 to day -6. For obesive or severely obesive patients Busilvex® should be administered based on adjusted ideal body weight.

Ideal body weight (IBW) is calculated as follows: (Height in cm, and weight in kilogram): IBW (kg; men) =50 + [0.91 x (height in cm - 152)]; IBW (kg, women) = 45 + [0.91 x (height in cm - 152)]. Adjusted ideal body weight (AIBW) should be calculated as follows: AIBW = IBW + [0.25 x (actual weight - IBW)].

Antiepileptic drugs should be used to prevent seizure with either Busulfan or Busilvex®.

14.1.1.2 CYCLOPHOSPHAMIDE

Cyclophosphamide at a dose of 120 mg/kg body weight will be given intravenously via a central venous catheter on day -4 and -3 at 60 mg/kg BW per day for a total dose of 120 mg/kg BW.

14.1.1.3 MESNA

Mesna to prevent haemorrhagic cystitis will be used according to the local practice.

14.1.2 ARM B (REDUCED CONDITIONING)

14.1.2.1 BUSULFAN

Busulfan at a total dose of 8 mg/kg BW (orally) or 6.4 mg/kg BW (intravenously) will be administered from day -7 to day -6 at a dose of 4 mg/kg BW (orally) or 3.2 mg/kg BW (intravenously) per day. The preferred application of Busulfan will be Busilvex® intravenously. Busilvex® should be administered intravenously via a central venous catheter, and the dose of Busilvex® is 0.8 mg/kg of ideal body weight or actual body weight, whichever is lower, administered as a two hour infusion every six hours from day -7 to day -6.

Ideal body weight (IBW) is calculated as follows: (Height in cm, and weight in kilogram): IBW (kg; men) =50 + [0.91 x (height in cm - 152)]; IBW (kg, women) = 45 + [0.91 x (height in cm - 152)]. Adjusted ideal body weight (AIBW) should be calculated as follows: AIBW = IBW + [0.25 x (actual weight - IBW)].

Antiepileptic drugs should be used to prevent seizure with either Busulfan or Busilvex®.

14.1.2.2 FLUDARABINE

Fludarabine at a dose of 30 mg/m² will be given intravenously as a short-infusion from day -7 to -3.

14.1.2.3 MESNA

Mesna to prevent haemorrhagic cystitis will be used according to the local practice.

14.2 FLOW SHEET

Randomisation

ARM A

Busilvex®, 12.8 mg/kg IBW i. v.:
day -9: 0.8 mg/kg IBW x 4 (every 6 hours)
day -8: 0.8 mg/kg IBW x 4 (every 6 hours)
day -7: 0.8 mg/kg IBW x 4 (every 6 hours)
day -6: 0.8 mg/kg IBW x 4 (every 6 hours)

or (if i. v.-application is not available):

Busulfan, 16.0 mg/kg BW p. o.:
day -9: 4.0 mg/kg BW
day -8: 4.0 mg/kg BW
day -7: 4.0 mg/kg BW
day -6: 4.0 mg/kg BW

plus:

Cyclophosphamide, 120 mg/kg BW i. v.:
day -4: 60 mg/kg BW
day -3: 60 mg/kg BW

ARM B

Busilvex®, 6.4 mg/kg IBW i. v.:
day -7: 0.8 mg/kg IBW x 4 (every 6 hours)
day -6: 0.8 mg/kg IBW x 4 (every 6 hours)

or (if i. v.-application is not available):

Busulfan, 8.0 mg/kg BW p. o.:
day -7: 4.0 mg/kg BW
day -6: 4.0 mg/kg BW

plus:

Fludarabine, 5 x 30 mg/m^2 BS i. v.:
day -7: 30 mg/m^2 BS
day -6: 30 mg/m^2 BS
day -5: 30 mg/m^2 BS
day -4: 30 mg/m^2 BS
day -3: 30 mg/m^2 BS

14.3 GvHD-Prophylaxis

The GvHD prophylaxis in all related transplantations consists of cyclosporine A and methotrexate as outlined below:

14.3.1 … in case of *related* donor

<u>Cyclosporine A</u>

Cyclosporine A will be started at a dose of 3 mg/kg BW per day as continuous infusion, starting on day -1, according to the local standard policy, subsequently adjusted to serum level which should be between 200 µg/l and 300 µg/l. Cyclosporine A will be switched orally, 3 mg/kg b.i.d. when tolerating oral medications, in the absence of GvHD.

In the standard conditioning arm (Arm A) and in the reduced conditioning arm (Arm B), cyclosporine A tapering should be started between day 100 and 120 in the absence of acute GvHD each two weeks and should be further tapered to be discontinued at day 180, provided that there is no evidence of GvHD.

Further dose reduction or withdrawal due to toxicity should be performed according to the local policy.

<u>Methotrexate</u>

Methotrexate will be given on day 1, day 3 and day 6 at a dose of 10 mg/m² intravenously. In case of severe mucositis and high bilirubin level the dose of methotrexate can be adjusted according to the local policy.

Standard conditioning arm / (Arm A)<br>Dose-reduced conditioning arm (Arm B)	Cyclosporine A	from day -1 until day +180<br>adjusted to serum level (200–300 µg/l)
MTX	10 mg/m²	day +1, day +3, +6

14.3.2 … in case of *unrelated* donor

After unrelated stem cell transplantation several GvHD prophylaxes are allowed, however, each center has to decide for **<u>only one strategy for all their patients</u>** included!

CYCLOSPORINE A

All patients will be given cyclosporine A in both arms from day -1 until day 180 as outlined below. Tapering should be started at day +100 to +120 in the absence of acute GvHD and should be further tapered each two weeks to be discontinued on day +180 subject there is no evidence of GvHD.

FOR ALL PATIENTS:

Standard conditioning arm	Cyclosporine A	from day -1 until day +180<br>adjusted to serum level (200–300 µg/l)
Dose-reduced conditioning arm	Cyclosporine A	from day -1 until day +180<br>adjusted to serum level (200–300 µg/l)

PLUS FURTHER GVHD PROPHYLACTIC TREATMENTS:

Beside this basic GvHD prophylaxis further GvHD prophylactic treatments are allowed:

EITHER:

Strategy 1: ***Cyclosporine A plus Campath***

Campath will be given at a total dose of 100 mg given as 20 mg each at day -8 to -4.

Standard conditioning arm / (Arm A)<br>Dose-reduced conditioning arm (Arm B)	Cyclosporine A	from day -1 until day +180<br>adjusted to serum level (200–300 µg/l)
Campath	100 mg	given as 20 mg each day -8 to -4

OR:

Strategy 2: ***Cyclosporine A plus Anti-Thymocyte-Globulin (Rabbit, Fresenius) plus Methotrexate***

A second possibility is to include anti-thymocyte-globulin (Rabbit, Fresenius) at a dose between 30 and 60 mg/kg BW according to the local policy (given divided in day -3 to -1 or full dose at day -1).

Beside ATG (rabbit, Fresenius), methotrexate given at a dose of 10 mg/m² should be given on day +1, +3, +6, +11.

Standard conditioning arm / (Arm A)<br>Dose-reduced conditioning arm (Arm B)	Cyclosporine A	from day -1 until day +180<br>adjusted to serum level (200–300 µg/l)
Anti-Thymocyte-Globulin (rabbit, Fresenius)	30-60 mg/kg BW	given as:<br>10 - 20 mg/kg BW each on day -3 to -1<br>or as full dose on day -1
MTX	10 mg/m²	day +1, +3, +6, +11

OR:

Strategy 3) ***Cyclosporine A plus Anti-Thymocyte-Globulin (rabbit, Thymoglobulin, Genzyme) plus Methotrexate***

Anti-thymocyte-globulin (rabbit, Thymoglobulin, Genzyme) at a dose between 6 and 10 mg/kg BW according to local practice given as 1.5 or 2.5 mg/kg BW injections each day on day -4 to day -1.

Additionally methotrexate at a dose of 10 mg/m² should be given on day +1, +3, +6 and +11 as outlined below.

Standard conditioning arm / (Arm A)<br>Dose-reduced conditioning arm (Arm B)	Cyclosporine A	from day -1 until day +180<br>adjusted to serum level (200–300 µg/l)
Anti-Thymocyte-Globulin (rabbit, Thymoglobulin, IMTIX SangStat)	6-10 mg/kg BW	given as 1,5 - 2,5 mg/kg BW each day -4 to -1
MTX	10 mg/m²	day +1, +3, +6, +11

OR:

Strategy 4) ***Cyclosporine A plus Methotrexate***

A last (forth) GvHD-prophylaxis can be methotrexate alone at a dose of 15 mg/m² at day +1 and 10 mg/m² at day +3, +6 and +11.

Standard conditioning arm / (Arm A)<br>Dose-reduced conditioning arm (Arm B)	Cyclosporine A	from day -1 until day +180<br>adjusted to serum level (200–300 µg/l)
MTX alone	15 mg/m²<br>10 mg/m²	day +1<br>day +3, +6, +11

14.4 GROWTH FACTORS (OPTIONAL)

Growth factors G-CSF	5 µg/kg BW	from day +1 or +5 (according to the local policy) until sustained leukocyte engraftment

14.5 INFECTION PROPHYLAXIS

- Fluconazole or Itraconazole from conditioning until engraftment according to the local policy
- Ofloxacin or Ciprofloxacin until engraftment according to the local policy
- Acyclovir according to the local policy
- Pre-emptive CMV-treatment with Gancyclovir in case of positive pp65-test
- Cotrimoxazole twice daily on weekends or alternatively monthly inhalation with Pentamidine up to one year after allogeneic transplant

14.6 BLOOD PRODUCTS

- For at least one year, all blood products have to be irradiated.
- CMV negative patients should be transfused with CMV-negative blood products only or transfused with adequate fitted system.

14.7 SUPPORTIVE CARE

- Antiemetic medication will be given according to the local practice procedures or institutional guidelines.
- Fluid administration and management will be done according to the local policy.
- Physical examination, vital signs, full blood count, biochemistry, cyclosporine dosage should be evaluated before starting the transplantation procedures and thereafter on a daily or weekly basis until leukocyte count reached > 1.0 x 10^9/l. Subsequent monitoring will bed done according to the local policy.
- Antiepileptic drugs (phenytoin or clonazepam) should be used to prevent seizure according the local practice in both treatments arms

14.8 CHIMERISM STUDIES

Chimerism studies by bone marrow and blood samples should be performed prior to transplantation, following engraftment at day 30, day 100, after stop of immunotherapy and at one year after transplantation. Chimerism tests should be performed either with variable number of tandem repeat regions (VNTR), in sex-mismatch-patients with fluorescence-in-situ-hybridisation (FISH) and – if possible – with sorted peripheral blood $CD3^+$ T-cells.

The definitions of mixed chimerism are:

⇒ **Mixed chimerism**: > *5 percent and* < *95 percent donor* $CD3^+$ *cells in peripheral blood T-cells.*

⇒ **Full donor chimerism**: > *95 percent donor* $CD3^+$ *cells in peripheral blood T-cells.*

15 ELIGIBILITY CRITERIA FOR DONOR LYMPHOCYTE INFUSION (DLI)

The eligibility criteria for donor lymphocyte infusion are as follows:

15.1 MIXED CHIMERISM OR IF T-CELL CHIMERISM DECREASES

- Mixed chimerism and withdrawal of all immunosuppressive therapy for at least three weeks.
- Decrease in T-cell or granulocyte chimerism at withdrawal of all immunosuppressive therapy for at least three weeks.

The initial dose of DLI is 5 x 10^5 $CD3^+$ cells per kilogram recipient's body weight in unrelated setting and 1 x 10^6 $CD3^+$ cells per kilogram recipient's body weight in related setting.

If complete chimerism is achieved within two months, no further infusions of DLI will be given.

If no complete chimerism occurs within two months, the next dose should be 1 x 10^6 $CD3^+$ cells per kilogram recipient's body weight in unrelated setting and 5 x 10^6 $CD3^+$ cells per kilogram recipient's body weight in related setting.

If no complete chimerism to the second DLI occurs, the third infusion after three months should contain 5 x 10^6 $CD3^+$ cells per kilogram recipient's body weight in unrelated setting and 1 x 10^7 $CD3^+$ cells per kilogram recipient's body weight in related setting.

If no response is seen after this DLI-scheme, further treatment is up to the participating centre.

15.2 PROGRESSION OR SYMPTOMATIC RELAPSE AFTER COMPLETE HAEMATOLOGICAL REMISSION

Progression or symptomatic relapse after complete haematological remission at any time after transplantation but after at least three weeks after withdrawal of immunosuppressive therapy.

The dose should be as mentioned above.

16 END OF STUDY

16.1 ... FOR THE PATIENT

- Patients may withdraw from the study at their request at any time.
- The investigator may withdraw patients if the toxicity is considered excessive or if there is evidence of disease progression while under treatment.
- the end of study for the individual patient is also 2 years from transplantation (last follow-up).

16.2 ... FOR THE WHOLE STUDY

- The whole study can be discontinued by the coordinating investigators in case of toxicity considered excessive. The study will be discontinued if in an interim analysis of the first 120 patients the Data Monitoring Committee decides the discontinuation.
- The end of study as a whole is 2 years post transplantation of the last randomized patient.

17 GVHD-SCORE

17.1 ACUTE GVHD

The scoring of acute GvHD will be performed according to the international criteria according to Glucksberg [13] (see Appendix 26.6 on page 55 and Appendix 26.7 on page 56).

GvHD grade II-IV should be treated according to the standard practice procedures or the institutional guidelines of the participating institutions.

17.2 CHRONIC GVHD

The scoring of chronic GvHD ("limited" or "extensive") will be performed according to the criteria according to Shulman [14] (see Appendix 26.8 on page 57).

Chronic GvHD should be treated according to the standard practice procedures or the institutional guidelines of the participating institutions.

18 EVALUATION OF TOXICITY

The evaluation of toxicity will be performed according to the Bearman-score (Appendix 26.5 on page 53).

19 REPORTING OF ADVERSE EVENT (AE) / SERIOUS ADVERSE EVENT (SAE)

In order to comply with the standards for Good Clinical Practice (GCP) it is important that investigators are aware of the different definitions related to adverse events and how to record, report and review each of these specific occurrences.

19.1 DEFINITIONS:

For the purpose of this protocol adverse events are classified in to the following categories:

19.1.1 ADVERSE EVENT (AE)

This is defined as any untoward medical occurrence in the patient/subject administered a medicinal product that does not necessarily have a causal relationship with this treatment. An AE is therefore described as any unfavourable and unintended sign (including abnormal laboratory results), symptom or disease temporally (timely) associated with the use of a medicinal product, whether or not related to the product.

19.1.2 SERIOUS ADVERSE EVENT (SAE)

A serious adverse event is defined as any untoward medical occurrence in the patient/subject administered a pharmaceutical product, which does not necessarily have a causal relationship with this treatment, and that at any dose:

a) Results in death

b) Is life threatening

c) Results in persistent or significant or disability/incapacity

d) Requires in-patient hospitalisation or prolongs existing hospitalisation.

e) Results in a congenital abnormality or birth defect.

f) Any other medical event considered serious by the treating physician.

19.1.3 ADVERSE DRUG REACTION (ADR)

This is defined as any noxious and unintended (harmful or unwanted) response to a medicinal product normally used in man for prophylaxis, diagnosis or therapy of diseases, or for modification of physiological function, and is suspected to be related to the drug.

19.1.4 SERIOUS ADVERSE DRUG REACTION (SADR)

This is defined as an adverse drug reaction that is serious (see SAE criteria above).

19.1.5 SUSPECTED UNEXPECTED SERIOUS ADVERSE (DRUG) REACTION (SUSAR)

The definition of a SUSAR is a serious adverse (drug) reaction, the nature or severity of which is not consistent with the applicable product information (e.g. as listed in the Drug information section).

A serious event or drug reaction is not defined as a SUSAR when:

a) it is serious but expected;

b) it does not fit the definition of a SAE or SADR, whether expected or not.

19.2 Recording, Reporting and Reviewing

It is the investigators responsibility to maintain an accurate and up to date record of all adverse events/occurrences in patients participating in the clinical trial. This record, including details of nature, onset, duration, severity, outcome and any relationship to investigational product, should be made on the relevant documentation as specified below. Medical terminology should always be used to describe any event. Investigators should avoid vague terms such as “sick”.

For details on assessing causality see section 19.3 below.

It is the investigators responsibility to review all events occurring at their site and as such the investigator must ensure that the patients are not compromised. Any appropriate action must be taken to protect the patients whilst ensuring validity of the results.

19.2.1 Reporting of Adverse Events (AE)

Adverse events (AEs and ADRs) will be reported on CRFs and sent to the Randomisation and Data Management Office of the CLWP (Leiden).

19.2.2 Reporting of Serious Adverse Events (SAE)

The local centers investigators must fax all SAE's to the Chronic Leukemia Working Party data office in Leiden **within 24 hours** of becoming aware of the event(s).
Faxnumber: +49 180 500 290 623 or +49 711 4900 8723. These SAE reports will be transmitted to the Coordinating Investigator. He will determine if the SAE is considered as a Serious Unexpected Suspected Adverse Reaction (SUSAR) and will provide the CLWP data office with the necessary documents.

Any SUSAR, from any source, which are considered by the Coordinating Investigator to be reportable to investigators, Health Authorities and Ethics Committees will be sent by the Coordinating Investigator to the CLWP data office within 12 calendar days or 5 calendar days for fatal or life-threatening reports. The CLWP data office will have the responsibility for

reporting such events to co-investigators and their Ethics Committees and to all applicable Health Authorities.

For both serious and non-serious adverse events, the investigator must determine both the intensity of the event and the relationship of the event to study drug administration. This will be reported on the CRF's

19.3 ASSESSMENT OF CAUSALITY

According to the new Clinical Trial Regulations 2004 the relationship of adverse events to the medicinal products being studied should be determined according to the classification below.

Not Related: Where a temporal (timely) relationship of the onset of the event, relative to the administration of the drug is not reasonable (e.g. cut finger). Or where another cause can explain the occurrence of the event by itself (e. g. headache associated with migraine).

Unlikely: Where a temporal (timely) relationship of the onset of the event, relative to the administration of the drug is unlikely but cannot be ruled out (e. g. mouth ulcer following admin of oral drug).

Possibly Related: Where a temporal (timely) relationship of the onset of the event, relative to the administration of the product is reasonable, but the event could have been due to an equally likely cause (e.g. headache).

Probably Related: Where a temporal (timely) relationship of the onset of the event, relative to the administration of the drug is reasonable and the event is more likely to be explained by the medicinal product than by another cause (e.g. nausea and vomiting).

Definitely Related: Where a temporal (timely) relationship of the onset of the event, relative to the administration of the drug is reasonable and there is no other cause to explain the event, or a re-challenge is positive (e.g. bone marrow depression following administration of cytotoxic chemotherapy).

Out of these five categories, “**possibly**”, “**probably**” and “**definitely**” related to a medicinal product qualify as **adverse reactions**. “**Unlikely**” and “**not related**” do not qualify as a reasonable causal relationship.

20 EVALUATION AFTER TRANSPLANTATION

All patients will be evaluated at day 100, six months, one year and two years after the date of transplantation.

At these visits the following evaluations must be performed:

Physical examination, vital signs, ECOG performance status (Appendix 26.10 on page 59), GvHD assessment (Appendices 26.6 to 26.8), toxicity, full blood count, biochemistry, bone marrow aspirate, cytogenetics (if abnormal at any time before transplantation), chimerism, and repeat of any investigations found to be abnormal at the previous assessment and attributed to the transplantation procedure.

Chimerism, bone marrow aspirate and cytogenetics are optional at six and 12 months after transplantation

Additional assessments may be necessary and will be performed at the discretion of the investigator. Additional follow-up assessments will be made as necessary, to establish normalisation or other explanation of any abnormal physical signs, symptoms or laboratory tests which are thought by the investigator to be attributable to the procedure or the disease.

20.1 CRITERIA TO DEFINE COMPLETE REMISSION

The criteria to define complete remission will be based on those proposed by Cheson et al.:

Bone marrow showing less than 5 % myeloblasts with normal maturation of all cell lines and no evidence for dysplasia (with erythroid precursors constitute less than 50 % of bone marrow nucleated cells, the percentage of blasts is based on all nucleated cells; when there are 50 % or more erythroid cells, the percentage blasts should be based on the non-erythroid cells), as well as peripheral blood showing no signs of dysplasia and no blasts, haemoglobin ≥ 11 g/dl (untransfused, patient not on erythropoietin), neutrophils ≥ 1,5 x 10^9/l (not on a myeloid growth

factor), platelets ≥ 100 x 10^9/l (not on a thrombopoietic agent), unless one of these cytopenias is related to a transplantation-related complication or its treatment.

20.2 CRITERIA TO DEFINE RELAPSE INCIDENCE

Relapse will be defined according to cytological criteria and/or cytogenetic analysis as the recurrence of any haematologic abnormalities, i.e., marrow evaluation showing more than 5 % myeloblasts, and / or abnormal cell line maturation with evidence of dysplasia (blast percentage is based on all nucleated cells; when there are 50 % or more erythroid cells, the percentage blasts should be based on the non-erythroid cells) and/or peripheral blood evaluation showing presence of abnormal peripheral blood blasts, signs of dysplasia, and unless the abnormal cell count is related to the transplantation procedure and/or to treatments used for transplantation-related complications: haemoglobin < 11 g/dl (untransfused, patient not on erythropoietin), neutrophils < 1,5 x 10^9/l (not on a myeloid growth factor), platelets < 10 x 10^9/l (not on a thrombopoietic agent). Cytogenetic relapse will be diagnosed if a pre-existing cytogenetic abnormality is detected (requires 20 analyzable metaphases using conventional cytogenetic techniques). Assessments will be performed at day +100, and by one and two years after transplantation.

The endpoint used for sample size estimation is the relapse incidence two years after transplantation.

20.3 CRITERIA TO DEFINE EVENT-FREE SURVIVAL

For assessment of the event-free survival, events will be either relapse or death, patients being censored in the other case (alive without relapse). The event-free survival will be assessed by two years after transplantation.

20.4 CRITERIA TO DEFINE OVERALL SURVIVAL

The overall survival will be assessed by two years after transplantation.

21 ENGRAFTMENT FAILURE

Engraftment failure will be considered if a stable leukocyte count > 0.5 x 10^9/l has not been reached on day 28 after graft infusion. If neutrophile recovery has not occurred by day 28, a bone marrow aspirate will be performed to confirm engraftment failure and to evaluate the type of chimerism present at that time. Further treatments or second transplantation will be considered according to local standard practices of each transplant center.

22 DATA RECORDING, MANAGEMENT AND MONITORING - FORMS AND PROCEDURES FOR COLLECTING DATA

22.1 PATIENT ENROLMENT

Before patients may be entered into the clinical trial, ethics committee approval of the protocol and informed consent forms must be obtained. The ethics committee approval letter along with the EBMT-Centre Trial Contract, Investigator-, Additional staff-, General requirements- and Laboratory normal ranges declarations should be sent to the data office before the first patient can be enrolled.

Once the patient and donor have signed the consent forms and the patient is ready to be enrolled in the study, the centre should fax the completed Patient Registration Form to:

CLWP Data Office
LUMC Postzone S-5-P
PO Box 9600
2300 RC Leiden
The Netherlands
Phone: +31-71-526-9722
Fax: +49-180-500-290-623
E-Mail: clwpebmt@lumc.nl

Patients may be enrolled Monday to Friday between 9am and 5pm. Faxes received after 5pm will be processed first thing the following week-day morning.

The data office will return a Registration Confirmation Form to the centre, assigning a patient study number. The centre confirms receipt and can commence the treatment.

22.2 REGULATORY AND ETHICAL OBLIGATIONS

The investigator will ensure that this study is conducted in full conformity with ICH GCP guidelines, and with the regulations and guidelines of the country in which the research is conducted.

22.2.1 INDEPENDENT ETHICS COMMITTEE / INSTITUTIONAL REVIEW BOARD

A copy of the protocol, proposed informed consent forms and other written patient information must be submitted to the IEC for written approval to local law. A copy of the written approval of the protocol and informed consent forms must be received by the data office before commencement of recruitment of patients into the trial protocol.

The investigator must, where necessary, submit and obtain approval from the IEC for all subsequent protocol amendments and changes to the informed consent document.

If required, the investigator will be responsible for obtaining annual IEC approval/renewal throughout the duration of the clinical trial.

22.3 DOCUMENTATION REQUIREMENTS PRE START OF TRIAL

The investigator is responsible for forwarding the following documents to the EBMT Data Office before study initiation can occur:

- Signed and dated protocol signature page.
- Copy of the IEC/IRB approval of the protocol and consent forms.
- Up-to-date curricula vitae of principal investigator and all co/sub investigators.
- Signed EBMT-Centre Trial Contract.
- Signed Principal Investigator-, additional staff-, general requirements- and laboratory normal ranges declaration.
- If locally required: copy of approval letter from Head of Department or Research & Development Department to participate in the study.

The CLWP Data Office will check insurance status. If not yet active, the CLWP will contact the centre as soon as the insurance is active, after which the centre may begin patient accrual to the study. I.e. no patient may be entered onto the study until all necessary approvals have been received by the CLWP Data office Leiden and the insurance policy has been activated.

Specific forms adapted from the EBMT MEDA/B form will be used to report details of treatment. The case report forms, as well as the donor and recipient information forms and informed consent forms, and the protocol are available from the EBMT-Chronic Leukaemia Working Party website in the Clinical Trial section:

http://www.ebmt.org/5WorkingParties/CLWP/clwpct.html

Each participating center will have to download and print these documents. Participating centers should return the forms to the Randomisation and Data Management Office of the CLWP (Leiden) as soon as they are completed. Case report forms must be completed in English using a black ball-point pen, and must be legible. Errors should be crossed by a single line but not obliterated, the correction inserted, and the change initialled and dated by the investigator or designee. Do not erase or write over errors. Do not use correction fluid or tape.

22.4 DATA MONITORING COMMITTEE

A data monitoring committee will regularly check study data to evaluate patient safety and will be informed of the results of the interim analysis regarding severe adverse events. The data monitoring committee will consist of a statistician (Simona Iacobelli, Universita La Sapienza, Rome, Italy) and Prof. Gösta Gahrton (Huddinge Hospital, Karolinska Institute, Stockholm, Sweden).

22.5 MONITORING

22.5.1 INITIATION VISITS

Initiation will be done via a phoneconference guided by a power point presentation. Sites will be informed on administrative procedures, documentation requirements, roles and responsibilities, pharmacovigilance and data collection. The contents of the protocol will be reviewed.

22.5.2 CLOSE-OUT VISITS

- Selected sites of the participating centers (20 – 30 %) will be visited by chance.
- The final visit to the site ensures that all study documentation and records are complete (including drug accountability and destruction records; subject identification list; data queries) and arrangements are in place for retention/archiving of these on or off-site, (including Investigator File and copies of CRFs), for the required timeframe.
- Confirmation that patient medical records are labelled to ensure retention by Medical Records
- Certification that all CRFs, data queries and unused drug/study supplies have been retrieved.

22.6 SCHEDULE TO COMPLETE REPORT FORMS

Case report forms must be completed according the following schedule:

22.6.1 AFTER FULFILLING THE INCLUSION CRITERIA

The patient must be registered by fax on the **Registration Form**.

22.6.2 DURING AND AFTER TREATMENT

The data should be registered on special CRF's on day +100 as well as 6, 12, 24 and 36 months after transplantation.

22.7 RECORD RETENTION REQUIREMENTS

The investigator must arrange for the retention of the patient identification codes for at least 15 years after the completion or discontinuation of the trial. Patient files and other source data pertaining to the conduct of the study must be kept for at least 15 years.

23 ETHICAL CONSIDERATIONS

23.1 PATIENTS' PROTECTION

The responsible investigator will ensure that this study is conducted in conformity with either the declaration of Helsinki (Version of Edinburgh, October 7, 2000) (see Appendix **Error! Reference source not found.** on page **Error! Bookmark not defined.**) or the laws and regulations of the country, whichever provides the greatest protection of the patient. The protocol has been written and the study will be conducted according to the ICH Harmonised Tripartite Guideline for Good Clinical Practice, issued by the European Union. The responsible local Ethic Committee approval must be obtained before starting the trial.

A copy of the informed consent form (with patient information sheet) must be submitted to the Ethics Committee together with the protocol for written approval. Written approval of the protocol and informed consent by the responsible Ethics Committee must be obtained prior to recruitment of patients to the study by each participating investigator. The investigator must inform the Ethics Committee of subsequent protocol amendments, which must be approved by the Committee.

23.2 Patient Confidentiality / Subject identification

The investigator must ensure that the confidentiality of records that could identify the patient should be protected, respecting the privacy and confidentiality rules in accordance with the applicable local regulatory requirements. On the case report forms or other study related documents, patients should be identified by their initials, date of birth, UPN and the patient study number only. Full patient names should never be used in any correspondence or communication with the Trials office or the study committee.

Direct access to review the patient's original medical records for verification of trial-related procedures and data will be required. Direct access includes examining, analysing, verifying, and reproducing any records and reports that are important to the evaluation of the study. The investigator is obliged to inform the patient that his/her trial-related records will be reviewed without violating the confidentiality of the patient.

23.2.1 Informed consent

An initial generic informed consent form for the patient is provided in Appendix 26.12 on page 83 (German language) and Appendix 26.13 on page 98 (English language) for the investigator to prepare the informed consent document to be used at his or her site. The written informed consent document should be prepared in the language(s) of the potential patient population.

Before a patient's participation in this prospective data collection, the investigator is responsible for obtaining written informed consent from the patient, or legally acceptable representative, after adequate explanation of the aims and methods. A legally acceptable representative is an individual or other body authorized under applicable law to consent, on behalf of a prospective patient, to the patient's participation in the clinical trial.

The acquisition of informed consent should be documented in the patient's medical records, as required by ICH GCP, and the informed consent form should be signed and personally dated by the patient, or a legally acceptable representative, and by the person who conducted the informed consent discussion. The original signed informed consent form should be retained in accordance with institutional policy, and a copy of the signed consent form should be provided to the patient or legally acceptable representative.

The patient information sheets and consent forms are enclosed (Appendix 26.12 (= German version) / Appendix 26.13 (= English version).

23.3 INSURANCE

Insurance coverage for participating patients is provided by the EBMT through Gerling Insurance Company. The policy documents can be requested when the local investigator is otherwise ready to submit the study to their Ethics Committee and Institutional Review Board for approval.

For information or to receive the relevant insurance documentation, contact:

CLWP Data Office
LUMC Postzone S-5-P
PO Box 9600
2300 RC Leiden
The Netherlands
Phone: +31-71-526-9722
Fax: +49-180-500-290-623
E-Mail: clwpebmt@lumc.nl

The insurance company (Germany) can only guarantee full insurance coverage – and payment in case of any claim – if the participating center fulfils all the legal requirements to run the study. Without approval from the participating center's national and/or local ethics committee (according to national law) the insurance policy is not valid.

24 PUBLICATION POLICY

The publication policy will be in accordance with the Authorship guidelines for EBMT publications (see EBMT Manual). Authorship will be based on the extent of participation in the study. Publications will list the following co-authors: coordinating investigator(s); author writing the manuscript (if not the coordinating investigator); members of the Chronic Leukemia Working Party who significantly contributed to conception, design, conduct of the trial and analysis of data, representatives of centres reporting at least 10% of enrolled patients or representatives of the 6-10 centres that enrolled most patients; statistician(s), involved in

design, analysis or interpretation of data, Working Party chairman, other persons who significantly contributed to the trial. A draft manuscript will be submitted to the Data Center and all co-authors (and the financial supporter, where applicable) for review. After revision by the Data Center, the other co-authors (and the financial supporter), the manuscript will be sent to a peer reviewed scientific journal. Interim publications or presentations of the study may include demographic data, overall results and prognostic factor analyses, but no comparisons between randomized treatment arms may be made publicly available before the recruitment is discontinued. Any publication, abstract or presentation based on patients included in this study must be approved by the coordinating investigator(s). This is applicable to any individual patient registered/randomized in the trial, or any subgroup of the trial patients. Such a publication cannot include any comparisons between randomized treatment arms nor an analysis of any of the study end-points unless the final results of the trial have already been published. The first publication will be a joint publication of the complete study population.

25 ADMINISTRATIVE AND LEGAL OBLIGATIONS

25.1 TRIAL DOCUMENTATION AND STORAGE

The investigator and trial staff are responsible for maintaining a comprehensive and centralized filing system of all trial-related (essential) documentation as defined in ICH GCP section 8.

Elements should include:

- Patient files containing completed case report forms, informed consents, and supporting copies of source documentation
- Trial files containing the protocol with all amendments, copies of trial documentation, and all correspondence to and from the IEC (if applicable).
- In addition, all original source documents supporting entries in the case report forms must be maintained and be readily available.

Once the trial has ended the investigator is obliged to keep the trial-related documents for 15 years. It is important that suitable archiving space is identified at the site. The investigator/institution should take measures to prevent accidental or premature destruction of these documents.

Literature

1 Greenberg PL. The myelodysplastic syndromes. In: Hoffman R, Benz E, Shattil S, et al. (eds): Hematology: Basic Principles and Practice. 3rd edn. Churchill Livingstone: New York. 2000; 1106-1129

2 Bennett J, Catovsky D, Daniel M, et al.: Proposals for the classification of the myelodysplastic syndromes. Br J Haematol 1982; 51: 189-199

3 Third MIC Cooperative Study Group. Recommendations for a morphologic, immunologic and cytogenetic (MIC) working classification of the primary and therapy related myelodysplastic disorders. Cancer Genet Cytogenet 1988; 32: 1-10

4 Greenberg P, Cox C, LeBeau M, et al.: International scoring system for evaluating prognosis in myelodysplastic syndrome. Blood 1997; 89: 2079-2083

5 De Witte T, Suciu S, Verhoef G, et al.: Intensive chemotherapy followed by allogeneic or autologous stem cell transplantation for patients with myelodysplastic syndromes (MDSs) and acute myeloid leukemia following MDS. Blood 2001; 98; 2326-2331

6 de Witte T, Hermans J, Vossen J, et al.: Haematopoietic stem cell transplantation for patients with myelo-dysplastic syndromes and secondary acute myeloid leukaemias: A report on behalf of the Chronic Leukaemia Working Party of the European Group for Blood and Marrow Transplantation (EBMT). Br J Haematol 2000; 110: 620-630

7 Appelbaum FR, Anderson J: Allogeneic bone marrow transplantation for myelodysplastic syndrome: Outcomes analysis according to IPSS score. Leukemia 1998, 12 (Suppl. 1): 25-29

8 Deeg HJ, Shulman HM, Anderson JE, et al.: Allogeneic and syngeneic marrow transplantation for myelodysplastic syndrome in patients 55 to 66 years of age. Blood 2000; 95: 1188-1194

9 Parker JE, Shafi T, Pagliuca A, et al.: Allogeneic stem cell transplantation in the myelodysplastic syndromes: Interim results of outcome following reduced-intensity conditioning compared with standard preparative regimen. Br J Haematol 2002; 119: 144-154

10 Martino R, Caballero MD, Simon JA, et al, AML and alloPBSCT Subcommittees of the Spanish Group for Hematopoietic Transplantation: Evidence for a graft-versus-leukemia effect after allogeneic peripheral blood stem cell transplantation with reduced-intensity conditioning in acute myelogenous leukemia and myelodysplastic syndromes. Blood 2002; 100 (6): 2243-2245

11 Kröger N, Bornhäuser M, Ehninger G, et al.: Allogeneic stem cell transplantation after a fludarabine/busulfan-based reduced-intensity conditioning in patients with myelodysplastic syndrome or secondary acute myeloid leukemia. Ann Hematol 2003; 82 (6): 336-342

12 Bearman et al, J Clin Oncol 1988; 6: 1562-1568

13 Glucksberg H, Storb R, Fefer A et al.: Clinical manifestations of graft-versus-host disease in human recipients of marrow from HLA-matched sibling donors. Transplant 1974; 18: 295-304

14 Shulman HM, Sullivan KM, Weiden PL, McDonald GB, Striker G, Sale GE, Hackmann R, Tsoi MS, Storb R, Thomas ED: Chronic graft-versus-host syndrome in Man. A long-term clinicopathologic study of 20 Seattle patients. Am J Med 1980; 69 (2): 204-217

15 McDonald GB, Hinds MS, Fisher LD, Schoch HG, Wolford JL, Banaji M, Hardin BJ, Shulman HM, and Clift RA: Veno-occlusive disease of the liver and multiorgan failure after bone marrow transplantation: A cohort study of 355 patients. Annals of Internal Medicine 1993; 118: 255-267

26 APPENDICES

26.1 SERIAL MEASUREMENTS AND STUDY CALENDAR

	Transplantation	Day 100 after SCT	6 months after SCT	1 year after SCT	2 years after SCT
Complete history	yes	---	---	yes	yes
Physical examination	yes	yes	yes	yes	yes
Vital signs[1]	yes	yes	yes	yes	yes
ECOG performance status	yes	yes	yes	yes	yes
Thoracic X-ray	yes	---	---	---	---
Cardiac evaluation[2]	yes	---	---	---	---
Full blood count	yes	yes	yes	yes	yes
Biochemistry[3]	yes	yes	yes	yes	yes
Bone marrow aspirate	yes	yes	optional	yes	yes
Bone marrow cytogenetics	yes	yes[4]	optional	optional	optional
IPSS score-value	yes	---	---	---	---
Virology[5]	yes	---	---	---	---
AB0 blood group	yes	---	---	---	---
HLA typing	yes	---	---	---	---
Chimerism[6]	---	yes	yes	yes	yes
β_2-Microglobulin	yes	yes	yes	yes	yes
Acute GvHD evaluation	---	yes	yes[7]	yes[7]	yes[7]
Chronic GvHD evaluation	---	---	yes	yes	yes

1) Vital signs: heart rate, blood pressure, temperature
2) Using either echocardiography or isotopic evaluation of the left ventricular ejection fraction
3) Biochemistry: Alkaline phosphatase, alanine and aspartate aminotransferases, lactate dehydrogenases, gamma-GT, total bilirubin, creatinine clearance, uric acid, glucose
4) If previously abnormal
5) Virology: Hepatitis B, Hepatitis C, CMV, EBV, HIV, HTLV1 serologies, HSV, toxoplasmosis
6) 10 – 20 ml peripheral blood in EDTA or according to local practise
7) If donor lymphocyte infusion(s) was/were given

26.2 RISK-SCORE ACC. TO THE INTERNATIONAL MDS WORKSHOP (IPSS)

Single factors

Prognostic factors	*Score*				
	0	*0,5*	*1,0*	*1,5*	*2,0*
Count of blasts in the bone marrow	< 5 %	5 – 10 %	5 – 10 %	11 – 20 %	21 – 30 %
Karyotype*	low risk	intermediate risk	high risk		
Involved cell lines**	0 – 1	2 – 3			

* *Low risk:* Normal karyotype, -Y, 5q-, 20q-.
High risk: Complex Karyotype, anomalies of Chromosome 7.
Intermediate risk: All other aberrations.

** Count of involved cell lines (granulocytopoiesis, erythrocytopoiesis, thrombocytopoiesis).

Risk groups

Risk group	*Score Sum*	*Risk of malignant transformation**	*Median time of survival*
Low risk	0	> 18	65 months
Intermediate 1 (Int 1)	0,5 – 1,0	8 years	40 months
Intermediate 2 (Int 2)	1,5 – 2,0	3 years	14 months
High risk	> 2,5	0,5 years	5 months

* Time until development of AML (median).

26.3 FAB Classification of myelodysplastic syndromes (acc. to Bennett)

The five types of MDS are

(1) refractory anaemia (RA)
(2) RA with ring sideroblasts
(3) RA with excess of blasts (RAEB)
(4) chronic myelomonocytic leukaemia (CMMoL)
(5) RAEB in transformation.

The features that characterised these forms of MDS are defined as follows:

26.3.1 Refractory anaemia (RA)

Anaemia is the main presenting symptom. The peripheral blood shows reticulocytopenia, variable dyserythropoiesis and infrequently dysgranulopoiesis. Blast cells are not seen in the peripheral blood; when present they do not exceed 1 ‰. The bone marrow is normo-, hypo- or hypercellular with erythroid hyperplasia and/or dyserythropoiesis. There are always fewer than 5 % of blast cells. Patients with neutropenia and/or thrombocytopenia but no anaemia can be included in this category.

26.3.2 Refractory anaemia (RA) with ring sideroblasts (RARS)

The main difference from the above is the presence of ringed sideroblasts accounting for more than 15 % of all nucleated erythropoietic cells in the bone marrow. Deficient haemoglobinisation in some of the red cell precursors leads to a dimorphic picture in peripheral blood films.

26.3.3 Refractory anaemia (RA)with excess of blasts (RAEB)

The age incidence is similar to that of RA. There is always some degree of cytopenia affecting two or more of the bone marrow series. The peripheral blood shows conspicuous abnormalities in all three cell lines. Dysgranulopoiesis, in contrast to RA, is a common feature. There may be a small proportion of circulating blasts (< 5 %). The bone marrow is hypercellular and shows varying degrees of either granulocytic or erythroid hyperplasia. There is always evidence of dysgranulopoiesis, dyserythropoiesis and/or dysmegakaryopoiesis; ringed sideroblasts may be seen. The percentage of blasts (type I and II) in the bone marrow, by definition, is equal to or greater than 5 % up to 20 %. There is almost always evidence of maturation in the granulocytic series to promyelocytes and beyond.

26.3.4 RAEB IN TRANSFORMATION (RAEB-T)

This type includes cases of cytopenia in patients of any age, often with symptoms of brief duration, which do not strictly fit into either of the above categories or in any of the AML types (M1 – M6). The haematological features are similar to those of RAEB but include any of the following:

- 5 % or more blasts in the peripheral blood,
- more than 20 % and up to 30 % of blasts (type I and II) in the bone marrow,
- presence of unequivocal Auer rods in granulocytic precursors.

26.3.5 CHRONIC MYELOMONOCYTIC LEUKAEMIA (CMMOL)

The defining feature is the presence of an absolute monocytosis (> 1 x 10^9/1). Often this is associated with an increase in mature granulocytes with or without evidence of dysgranulo-poiesis (e. g. hypogranular and/or Pelger forms). The percentage of blasts in the peripheral blood is less than 5 %. The bone marrow resembles that of RAEB but may show a significant increase in monocytic precursors (promonocytes) often with fewer than 5 % of blasts. In some patients with moderate monocytosis and bone marrow features identical of those of RAEB, the percentage of blasts may be higher than 5 %, up to 20 %.

26.4 MYELODYSPLASTIC SYNDROME CLASSIFICATION 2000 (WHO)

World Health Organization Classification and Criteria for the Myelodysplastic Syndromes (MDS)

MDS Subtype	Blood Findings	Bone Marrow Findings
Refractory anemia (RA)	Anemia<br>No or rare blasts	Erythroid dysplasia *only*<br><5% blasts<br><15% ringed sideroblasts
Refractory anemia with ringed sideroblasts (RARS)	Anemia<br>No blasts	Erythroid dysplasia *only*<br>≥15% ringed sideroblasts<br><5% blasts
Refractory cytopenia with multilineage dysplasia (RCMD)	Cytopenias (bi- or pancytopenia)<br>No or rare blasts<br>No Auer rods<br><1 x 10^9/L monocytes	Dysplasia in ≥10% of cells in 2 or more myeloid cell lines<br><5% blasts in marrow<br>No Auer rods<br><15% ringed sideroblasts
Refractory cytopenia with multilineage dysplasia and ringed sideroblasts (RCMD-RS)	Cytopenias (bi- or pancytopenia)<br>No or rare blasts<br>No Auer rods<br><1 x 10^9/L monocytes	Dysplasia in ≥10% of cells in 2 or more myeloid cell lines<br>≥15% ringed sideroblasts<br><5% blasts<br>No Auer rods
Refractory anemia with excess blasts-1 (RAEB-1)	Cytopenias<br><5% blasts<br>No Auer rods<br><1 x 10^9/L monocytes	Unilineage or multilineage dysplasia<br>5% to 9% blasts<br>No Auer rods
Refractory anemia with excess blasts-2 (RAEB-2)	Cytopenias<br>5% to 19% blasts<br>Auer rods ±<br><1 x 10^9/L monocytes	Unilineage or multilineage dysplasia<br>10%-19% blasts<br>Auer rods ±
MDS associated with isolated del(5q)	Anemia<br><5% blasts<br>Platelets normal or increased	Normal to increased megakarocytes with hypolobated nuclei<br><5% blasts<br>No Auer rods<br>Isolated del(5q)
Myelodysplastic syndrome, unclassified (MDS-U)	Cytopenias<br>No or rare blasts<br>No Auer rods	Unilineage dysplasia in granulocytes or megakaryocytes<br><5% blasts<br>No Auer rods

Type of Disease	Blood Findings	Bone Marrow Findings
Chronic myelomonocytic leukaemia (CMML)	< 5% blasts	< 20% blasts<br>> 1000/µl monocytes
Myelodysplastic syndrome, secondary Acute Myeloid Leukemia (sAML)	> 20% blasts	

26.5 ORGAN-RELATED TOXICITY OF CONDITIONING ACC. TO BEARMAN ET AL.

ORGAN	GRADE	SYMPTOMS
CARDIAC<br>☐ none	☐ I	• mild ECG abnormality, not requiring medical intervention<br>• or noted heart enlargement on chest x-ray, with no clinical symptoms
	☐ II	• moderate ECG abnormalities requiring and responding to medical intervention<br>• or requiring continuous monitoring without treatment<br>• or congestive heart failure responsive to digitalis or diuretics
	☐ III	• severe ECG abnormalities with no or only partial response to medical intervention<br>• or heart failure with no or only minor response to medical intervention<br>• or decrease in voltage by more than 50 %
BLADDER<br>☐ none	☐ I	• macroscopic haematuria after 2 days from last chemotherapy dose with no subjective symptoms of cystitis and not caused by infections
	☐ II	• macroscopic haematuria after 7 days from last chemotherapy not caused by infection<br>• or haematuria after 2 days with subjective symptoms of cystitis not caused by infection
	☐ III	• macroscopic haematuria with frank blood, necessitating invasive local intervention with installation of sclerosing agent, nephrostomy or other surgical procedure
RENAL<br>☐ none	☐ I	• increase in creatinine up to twice the baseline value (usually the last recorded before start of conditioning)
	☐ II	• increase in creatinine above twice the baseline value but not requiring dialysis
	☐ III	• requirement of dialysis
PULMONARY<br>☐ none	☐ I	• dyspnoea without chest x-ray changes not caused by infection or congestive heart failure<br>• or chest x-ray showing isolated infiltrate or mild interstitial changes without symptoms not caused by infection or congestive heart failure
	☐ II	• chest x-ray with extensive localised infiltrate or moderate interstitial changes combined with dyspnoea and not caused by infection or congestive heart failure<br>• or decease of pO_2 (> 10 % from baseline) but not requiring mechanical ventilation and not caused by infection or congestive heart failure<br>• or > 50 % O_2 on mask and not caused by infection or congestive heart failure
	☐ III	• interstitial changes requiring mechanical ventilatory support or > 50 % oxygen on mask and not caused by infection or and not caused by infection or congestive heart failure

Continuation: Organ-related toxicity of conditioning acc. to Bearman et al.

ORGAN	GRADE	SYMPTOMS
HEPATIC<br>☐ none	☐ I	• mild hepatic dysfunction with bilirubine ≥ 2 mg/dl and ≤ 6 mg/dl<br>• or weight gain > 2,5 % and < 5 % from baseline, of non-cardiac origin<br>• or SGOT increase by > 2-fold and < 5-fold from lowest preconditioning
	☐ II	• moderate hepatic dysfunction with bilirubine ≥ 6 mg/dl and ≤ 20 mg/dl<br>• or SGOT increase > 5-fold from lowest preconditioning<br>• or clinical ascites or image documented ascites > 100 ml<br>• or weight gain > 5 % from baseline, of non-cardiac origin
	☐ III	• severe hepatic dysfunction with bilirubine ≥ 20 mg/dl<br>• or hepatic encephalopathy<br>• or ascites compromising respiratory function
CNS<br>☐ none	☐ I	• somnolence but the patient is easily arousable and oriented after arousal
	☐ II	• somnolence with confusion after arousal<br>• or other new objective CNS symptoms with no loss of consciousness not more easily explained by other medication, bleeding or CNS infection
	☐ III	• seizures of coma not explained (documented) by other medication, CNS infection or bleeding
STOMATITIS<br>☐ none	☐ I	• pain and/or ulceration not requiring a continuous i. v. narcotic drug
	☐ II	• pain and/or ulceration requiring a continuous i. v. narcotic drug (morphine drip)
	☐ III	• severe ulceration requiring preventive intubation<br>• or resulting in documented aspiration pneumonia with or without intubation
GI-SYSTEM<br>☐ none	☐ I	• water stools > 500 ml and < 2000 ml every day, not related to infection
	☐ II	• water stools > 2000 ml every day, not related to infection<br>• or macroscopic stool with no effect on cardiovascular status, not caused by infection<br>• or subileus not related to infection
	☐ III	• ileus requiring nasogastric suction and / or surgery and not related to infection<br>• or haemorrhagic enterocolitis affecting cardiovascular status and requiring transfusion, not related to infection
**NOTE: GRADE IV- REGIMEN-RELATED TOXICITY IS DEFINED AS FATAL TOXICITY**		

26.6 ACUTE GVHD-STAGING (GLUCKSBERG ET AL., 1974)

STAGE	SKIN	LIVER	INTESTINAL TRACT
☐ 0	no rash	Bilirubin < 2 mg/dl	< 500 ml diarrhoea / d
☐ +	maculopapular rash < 25 % of body surface	Bilirubin 2-3 mg/dl	> 500 ml diarrhoea / d
☐ ++	maculopapular rash < 25 – 50 % of body surface	Bilirubin 3-6 mg/dl	> 1000 ml diarrhoea / d
☐ +++	generalised erythroderma	Bilirubin 6 – 15 mg/dl	> 1500 ml diarrhoea / d
☐ ++++	generalised erythroderma with bullous formation and desquamation	Bilirubin > 15 mg/dl	severe abdominal pain ± ileus

26.7 ACUTE GVHD-GRADING (GLUCKSBERG ET AL., 1974)

GRADE	DEGREE OF ORGAN INVOLVEMENT
☐ I	• skin rash: + to ++<br>• no gut involvement<br>• no liver involvement<br>• no decrease in clinical performance
☐ II	• skin rash: + to +++<br>• gut involvement: + and / or liver involvement<br>• mild decrease in clinical performance
☐ III	• skin rash: ++ to ++++<br>• gut involvement: ++ to +++ and /or liver involvement ++ to ++++<br>• marked decrease in clinical performance
☐ IV	• Similar to grade III with<br>– organ involvement ++ to ++++ and<br>– extreme decrease in clinical performance

26.8 Chronic GvHD-Grading (acc. to Shulman)

**Chronic GvHD may be defined as *limited* or *extensive* using the following criteria defined by Shulman**	
**Limited Chronic GvHD**	*Either or both criteria must be present:*<br>• Localised skin involvement<br>• Hepatic dysfunction
**Extensive Chronic GvHD**	*Either:*<br>• Generalised skin involvement<br>***or***<br>• Localised skin involvement and / or hepatic dys-function<br>• plus<br>Liver histology showing chronic aggressive hepatitis, bridging necrosis or cirrhosis<br>***or***<br>Involvement of eye: Schirmer's test with < 5 mm wetting,<br>***or***<br>Involvement of minor salivary glands or oral mucosa demonstrated on labial biopsy specimen,<br>***or***<br>Involvement of any other target organ (e. g., oeso-phageal abnormalities, polymyositis)

26.9 VOD-Criteria and -Grading (McDonald et al.)

**Criteria for clinical diagnosis of VOD**
• Hyperbilirubinaemia > 2 mg/dl<br>• Hepatomegaly or liver-dependent pain in the right top quadrant<br>• Gain of weight > 2 % of baseline because of fluid retention
For confirmation of clinical diagnosis of VOD two of the above three criteria must be ful-filled.<br>No other reasons for the above symptoms may exist.<br>Appearance of the above symptoms within 20 days after transplantation.

**Grading of VOD**
Mild VOD<br>• No need of diuretics<br>• No hepatic dysfunctions<br>• Complete normalisation of pathological parameters
Moderate VOD<br>• Need of diuretics<br>• Hepatic dysfunctions<br>• Perhaps complete normalisation of pathological parameters
Severe VOD<br>• Need of diuretics<br>• Hepatic dysfunctions<br>• No normalisation of pathological parameters until day +100 or until death

26.10 ECOG-Performance Status Scale

Grade	ECOG
0	Fully active, able to carry on all pre-disease performance without restriction.
1	Restricted in physically strenuous activity but ambulatory and able to carry out work of a light or sedentary nature (e. g. light house work, office work).
2	Ambulatory and capable of all self-care but unable to carry out any work activities. Up and about more than 50 % of waking hours.
3	Capable of only limited self-care, confined to bed or chair more than 50 % of waking hours.
4	Completely disabled. Cannot carry on any self-care. Totally confined to bed or chair.
5	Dead.

26.11 DRUG INFORMATION

26.11.1 BUSULFAN

Nomenclature
Generic name: Busulfan
Commercial name: Myleran
Chemical name: 1,4-butanediol dimethanesulfonate
Drug class, mechanism of action
Busulfan is a bifunctional alkylating agent in which two labile methanesulfonate groups are attached to opposite ends of a four carbon alkyl chain. In aqueous media, busulfan hydrolyzes to release the methanesulfonate groups. This produces reactive carbonium ions that can alkylate DNA. DNA damage is thought to be responsible for much of the cytotoxicity of busulfan. The molecular weight of busulfan is 246.31 kd.
Form
2-mg scored tablets. Busulfan is a white crystalline powder. It is only slightly soluble in water or ethanol.
Storage
Store tablets at room temperature and discard after expiration date.
Mixing instructions
None.
Drug interactions
Itraconazole decreases busulfan clearance by up to 25%, and may produce AUCs > 1500 µM·min in some patients. Phenytoin increases the clearance of busulfan by 15% or more, possibly due to the induction of glutathione-S-transferase. Use of other anticonvulsants may result in higher busulfan plasma AUCs, and an increased risk of VOD or seizures. Because busulfan is eliminated from the body via conjugation with glutathione, use of acetaminophen prior to (<72 hours) or concurrent with Busulfan may result in reduced busulfan clearance based upon the known property of acetaminophen to decrease glutathione levels in the blood and tissues. Fluconazole, and the 5-HT3 antiemetic ondansetron (Zofran) and granisetron (Kytril) have all been used with Busulfan.
Metabolism, pharmacokinetics
Busulfan is well absorbed orally, although variability in absorption, with bioavailability ranging from 47% to 120%, has been reported. Although food can delay absorption, it does not appear to change the overall AUC after an oral dose. Peak levels in serum occurring at approximately 1 hour. The clearance rate (Cl/F) for busulfan is 175 mL per minute in adults; it is two to four times higher in infants, 450 to 700 mL per minute, even when adjusted for body surface area. The elimination half-life is approximately 2.5 hours, but there is wide interpatient variability. Busulfan metabolism may vary with circadian rhythm, resulting in higher clearance rates and lower concentrations in the evening in some patients; this is more prominent in younger patients than in adults. Busulfan is lipophilic and crosses into CSF; concentrations at steady state in the cerebrospinal fluid of patients who receive BMT doses are 0.95 to 1.3 times plasma concentrations. Reversible protein binding is negligible for busulfan. Irreversible binding to plasma elements, primarily albumin, has been estimated to be 32.4 ± 2.2% which is consistent with the reactive electrophilic properties of busulfan. Busulfan is predominantly metabolised by conjugation with glutathione, both spontaneously and by glutathione S-transferase (GST) catalysis. This conjugate undergoes further extensive oxidative metabolism in the liver. Its major metabolic products (at least a dozen have been identified) are inactive. Busulfan clearance has been demonstrated to be higher in children than in adults. In infants, therapeutic monitoring identified relative underdosing when providing doses on a per-kg rather than a per-m2 regimen. This has necessitated the development of alternative dosing regimens for oral busulfan in this population.
Excretion
Following administration of 14 C- labelled busulfan to humans, approximately 30% of the radioactivity was excreted into the urine over 48 hours; negligible amounts were recovered in feces. The incomplete recovery of radioactivity may be due to the formation of long-lived metabolites or due to non-specific alkylation of macromolecules

Toxicity

Treatment with Busulfan at the recommended dose and schedule will result in profound myelosuppression in 100% of patients, including granulocytopenia, thrombocytopenia, anaemia, or a combined loss of formed elements of the blood. Profound myelosuppression, partly chronic and cumulative, is dose limiting. Gastrointestinal toxicities are frequent and generally considered to be related to the drug, including nausea, vomiting, anorexia, esophagitis, constipation, diarrhoea, stomatitis, abdominal pain, and dyspepsia. Other common toxicities are alopecia - sometimes irreversible - hyperpigmentation, mild or moderate oedema (hypervolemia or weight increase), and elevated liver function findings (veno-occlusive disease of the liver at transplant doses). Current literature suggests that high busulfan area under the plasma concentration verses time curve (AUC) values (>1,500 mMCmin) may be associated with an increased risk of developing hepatic veno-occlusive disease (VOD). Patients who have received prior radiation therapy, greater than or equal to three cycles of chemotherapy or a prior progenitor cell transplant may be at an increased risk of developing hepatic VOD with the recommended Busulfan dose and regimen. The incidence of VOD reported in the literature from the randomised, controlled trials was 7.7%-12%. Fever and infection - potentially life-threatening - are also usual complications. Neurological toxicity, including blurred vision, dizziness, insomnia, anxiety, and confusion, and interstitial lung disease with pulmonary fibrosis, and cardiac tamponade are less common. Patient can experienced a seizure while receiving cyclophosphamide, despite prophylactic treatment with phenytoin. Haematemesis and pancreatitis are rare events.

Indications

Regular dose therapy in CML (FDA-approved) and polycythaemia vera. High-dose therapy in bone marrow transplant.

Contraindications

Busulfan is contraindicated in patients with a history of hypersensitivity to any of its components.

Dosing - Administration

Busulfan is administered orally. Busulfan clearance is best predicted when the Busulfan dose is administered based on adjusted ideal body weight. Dosing Busulfan based on actual body weight, ideal body weight or other factors can produce significant differences in Busulfan clearance among lean, normal and obese patients. The usual adult dose of BUSULFAN as a component of a conditioning regimen prior to bone marrow or peripheral blood progenitor cell replacement support is 1 mg/kg of ideal body weight or actual body weight, whichever is lower, administered every 6 hours for 4 days (a total of 16 doses). For obese or severely obese patients, Busulfan should be administered based on adjusted ideal body weight. Ideal body weight (IBW) should be calculated as follows (height in cm, and weight in kg): IBW (kg; men)= 50 + 0.91x(height in cm -152); IBW(kg, women)= 45 + 0.91x(height in cm -152). Adjusted ideal body weight (AIBW) should be calculated as follows: AIBW = IBW +0.25x(actual weight - IBW). Prophylactic anticonvulsant therapy is routinely used with BMT preparative regimens. Antiemetics should be administered prior to the first dose of Busulfan and continued on a fixed schedule through administration of Busulfan.

Warnings – Recommendations - Precautions

High-dose Busulfan should be administered under the supervision of a qualified physician experienced in haematopoietic stem cell transplantation. Appropriate management of complications arising from its administration is possible only when adequate diagnostic and treatment facilities are readily available. The following warnings pertain to different physiologic effects of Busulfan in the setting of allogeneic transplantation. Frequent complete blood counts, including white blood cell differentials, and quantitative platelet counts should be monitored during treatment and until recovery is achieved. Antibiotic therapy and platelet and red blood cell support should be used when medically indicated. Seizures have been reported in patients receiving high-dose oral busulfan at doses producing plasma drug levels. Despite prophylactic therapy with phenytoin, seizure can be observed. Anti-convulsant prophylactic therapy should be initiated prior to Busulfan treatment. Caution should be exercised when administering the recommended dose of Busulfan to patients with a history of a seizure disorder or head trauma or who are receiving other potentially epileptogenic drugs. To detect hepatotoxicity, which may herald the onset of hepatic VOD, serum transaminases, alkaline phosphatase, and bilirubin should be evaluated daily through transplant day 28. The increased risk of a second malignancy should be explained to the patient. There is no known antidote to Busulfan other than haematopoietic progenitor cell transplantation. In the absence of haematopoietic progenitor cell transplantation, the recommended dosage for Busulfan would constitute an overdose of busulfan. The principal toxic effect is profound bone marrow hypoplasia / aplasia and pancytopenia but the central nervous system, liver, lungs, and gastrointestinal tract may be affected. The haematological status should be closely monitored and vigorous supportive measures instituted as medically indicated. Inadvertent administration of a greater than normal dose of oral busulfan (2.1 mg/kg; total dose of 23.3 mg/kg) occurred in a 2-year old child prior to a scheduled bone marrow transplant without sequelae. An acute dose of 2.4 g was fatal in a 10-year old boy. There is one report that busulfan is dialyzable, thus dialysis should be considered in the case of overdose. Busulfan is metabolised by conjugation with glutathione, thus administration of glutathione may be considered.

26.11.2 BUSILVEX

1. NAME OF THE MEDICINAL PRODUCT
Busilvex 6 mg/ml concentrate for solution for infusion

2. QUALITATIVE AND QUANTITATIVE COMPOSITION
1 ml of concentrate contains 6 mg of busulfan (60 mg in 10 ml). After dilution: 1 ml of solution contains 0.5 mg of busulfan For excipients see 6.1.

3. PHARMACEUTICAL FORM
Concentrate for solution for infusion. Clear, colourless solution.

4. CLINICAL PARTICULARS
4.1 Therapeutic indications
Busilvex followed by cyclophosphamide (BuCy2) is indicated as conditioning treatment prior to conventional haematopoietic progenitor cell transplantation (HPCT) in adult patients when the combination is considered the best available option.

4.2 Posology and method of administration
Busilvex administration should be supervised by a physician experienced in conditioning treatment prior to haematopoietic progenitor cell transplantation.

Dosage in adults
When followed by 2 cycles of 60 mg/kg body weight (BW) cyclophosphamide the recommended dosage and schedule of administration is 0.8 mg/kg BW of busulfan as a two-hour infusion every 6 hours over 4 consecutive days for a total of 16 doses prior to cyclophosphamide and conventional haematopoietic progenitor cell transplantation (HPCT)
It is recommended that cyclophosphamide dosing should not be initiated for at least 24 hours following the 16th dose of Busilvex (see 4.5).

Children and adolescents
The safety and efficacy of Busilvex in children and adolescents have not been established.

Administration
Busulfex must be diluted prior to administration (see 6.6). A final concentration of approximately 0.5 mg/ml busulfan should be achieved. Busilvex should be administered by intravenous infusion via central venous catheter.
Busilvex should not be given by rapid intravenous, *bolus* or peripheral injection.
All patients should be pre-medicated with anticonvulsant medicinal products to prevent seizures reported with the use of high dose busulfan. In the Busilvex studies, all patients received phenytoin for this purpose. There is no experience with other anticonvulsant agents such as benzodiazepines (see 4.4 and 4.5).
Antiemetics should be administered prior to the first dose of Busilvex and continued on a fixed schedule according to local practice through its administration.

Obese patients
For obese patients, dosing based on adjusted ideal body weight (AIBW) should be considered.
Ideal body weight (IBW) is calculated as follows: IBW men (kg)=50 + 0.91x (height in cm-152); IBW women (kg)= 45 + 0.91x (height in cm-152).
Adjusted ideal body weight (AIBW) is calculated as follows: AIBW= IBW+0.25x (actual body weight - IBW).

Renally impaired patient:
Studies in renally impaired patients have not been conducted, however, as busulfan is moderately excreted in the urine, dose modification is not recommended in these patients.
However, caution is recommended (see 4.8 and 5.2).

Hepatically impaired patient:
Busilvex as well as busulfan has not been studied in patients with hepatic impairment.
Caution is recommended, particularly in those patients with severe hepatic impairement (see 4.4).

Elderly patient:
Patients older than 50 years of age (n=23) have been successfully treated with Busilvex without dose-adjustment. However, for the safe use of Busilvex in patients older than 60 years only limited information is available. Same dose (see 5.2) for elderly as for adults (<50 years old) should be used.

4.3 Contraindications
Hypersensitivity to the active substance or to any of the excipients Pregnancy and lactation (see 4.6)

4.4 Special warnings and special precautions for use
The consequence of treatment with Busilvex at the recommended dose and schedule is profound myelosuppression, occurring in all patients. Severe granulocytopenia, thrombocytopenia, anaemia, or any combination thereof may develop. Frequent complete blood counts, including differential white blood cell counts, and platelet counts should be monitored during the treatment and until recovery is achieved. Absolute neutrophil counts < $0.5x10^9/l$ at a median of 4 days post transplant occurred in 100% of patients and recovered at median day 10 and 13 days following autologous and allogeneic transplant respectively (median neutropenic period of 6 and 9 days respectively). Prophylactic or empiric use of anti-infectives (bacterial, fungal, viral) should be considered for the prevention and management of infections during the neutropenic period. Thrombocytopenia (< $25,000/mm^3$ or requiring platelet transfusion) occurred at a median of 5-6 days in 98% of patients. Anaemia (haemoglobin<8.0 g/dl) occurred in 69% of patients. Platelet and red blood cell support, as well as the use of growth factors such as G-CSF, should be employed as medically indicated.
Busilvex as well as busulfan has not been studied in patients with hepatic impairment. Since busulfan is mainly metabolized through the liver, caution should be observed when Busilvex is used in patients with pre-existing impairment of liver function, especially in those with severe hepatic impairment. It is recommended when treating these patients that serum transaminase, alkaline phosphatase, and bilirubin should be monitored regularly 28 days following transplant for early detection of hepatotoxicity.
Hepatic veno-occlusive disease is a major complication that can occur during treatment with Busilvex. Patients who have received prior radiation therapy, greater than or equal to three cycles of chemotherapy, or prior progenitor cell transplant may be at an increased risk (see 4.8).
Caution should be exercised when using paracetamol prior to (less than 72 hours) or concurrently with Busilvex due to a possible decrease in the metabolism of busulfan (See 4.5).
As documented in clinical studies, no treated patients experienced cardiac tamponade or other specific cardiac toxicities related to Busilvex. However cardiac function should be monitored regularly in patients receiving Busilvex (see 4.8).
Occurrence of acute respiratory distress syndrome with subsequent respiratory failure associated with interstitial pulmonary fibrosis was reported in Busilvex studies in one patient who died, although, no clear etiology was identified. In addition, busulfan might induce pulmonary toxicity that may be additive to the effects produced by other cytotoxic agents. Therefore, attention should be paid to this pulmonary issue in patients with prior history of mediastinal or pulmonary radiation (see 4.8).
Periodic monitoring of renal function should be considered during therapy with Busilvex (see 4.8).
Seizures have been reported with high dose busulfan treatment. Special caution should be exercised when administering the recommended dose of Busilvex to patients with a history of seizures. Patients should receive adequate anticonvulsant prophylaxis. All data with Busilvex were obtained using phenytoin. There are no data available on the use of other anticonvulsant agents such as benzodiazepines. Thus, the effect of anticonvulsant agents (other than phenytoin) on busulfan pharmacokinetics is not known, (see 4.2 and 4.5).
The increased risk of a second malignancy should be explained to the patient. On the basis of human data, busulfan has been classified by the International Agency for Research on Cancer (IARC) as a human carcinogen. The World Health Association has concluded that there is a causal relationship between busulfan exposure and cancer. Leukaemia patients treated with busulfan developed many different cytological abnormalities, and some developed carcinomas. Busulfan is thought to be leukemogenic.
Fertility: busulfan can impair fertility. Therefore, men treated with Busilvex are advised not to father a child during and up to 6 months after treatment and to seek advice on cryo-conservation of sperm prior to treatment because of the possibility of irreversible infertility due to therapy with Busilvex. Ovarian suppression and amenorrhoea with menopausal symptoms commonly occur in pre-menopausal patients. Busulfan treatment in a pre-adolescent girl prevented the onset of puberty due to ovarian failure. Impotence, sterility, azoospermia, and testicular atrophy have been reported in male patients. The solvent dimethylacetamide (DMA) may also impair fertility. DMA decreases fertility in male and female rodents (see 4.6 and 5.3)

4.5 Interaction with other medicinal products and other forms of interaction
No specific clinical trial was carried out to assess drug-drug interaction between i.v. busulfan and itraconazole. From published studies, administration of itraconazole to patients receiving high-dose busulfan may result in reduced busulfan clearance. Patients should be monitored for signs of busulfan toxicity when itraconazole is used as an antifungal prophylaxis with i.v. busulfan.
Published studies described that ketobemidone (analgesic) might be associated with high levels of plasma busulfan. Therefore special care is recommended when combining these two drugs.

For the BuCy2 regimen it has been reported that the time interval between the last oral busulfan administration and the first cyclophosphamide administration may influence the development of toxicities. A reduced incidence of Hepatic Veino Occlusive Disease (HVOD) and other regimen-related toxicity have been observed in patients when the lag time between the last dose of oral busulfan and the first dose of cyclophosphamide is > 24hours.
Paracetamol is described to decrease glutathione levels in blood and tissues, and may therefore decrease busulfan clearance when used in combination (see 4.4).
Phenytoin was administered for seizure prophylaxis in all patients in the clinical trials conducted with i.v. busulfan. The concomitant systemic administration of phenytoin to patients receiving high-dose busulfan has been reported to increase busulfan clearance, due to induction of glutathion-S-transferase. However no evidence of this effect has been seen in i.v. data.(see 4.4)
No interaction has been reported when benzodiazepines such as diazepam, clonazepam or lorazepam have been used to prevent seizures with high-dose busulfan (see 4.2 and 4.4).
No interaction was observed when busulfan was combined with fluconazole (antifungal agent) or 5 -HT_3 anti-emetics such as ondansetron or granisetron.

4.6 Pregnancy and lactation

Pregnancy

HPCT is contraindicated in pregnant women ; therefore, Busilvex is contraindicated during pregnancy.
Busulfan has caused embryofoetal lethality and malformations in pre-clinical studies.(see 5.3)
There are no adequate and well-controlled studies of either busulfan or DMA in pregnant woman. A few cases of congenital abnormalities have been reported with low-dose oral busulfan, not necessarily attributable to the drug, and third trimester exposure may be associated with impaired intrauterine growth.
Women of childbearing potential have to use effective contraception during and up to 6 months after treatment.

Lactation

Patient who are taking Busilvex would not breast-feed. It is not known whether busulfan and DMA are excreted in human milk. Because of the potential for tumorigenicity shown for busulfan in human and animal studies, breast-feeding should be discontinued at the start of therapy.

4.7 Effects on ability to drive and use machines

Not relevant

4.8 Undesirable effects

Adverse events informations are derived from two clinical trials (n=103) of Busilvex. Serious toxicities involving the hematologic, hepatic and respiratory systems were considered as expected consequences of the conditioning regimen and transplant process. These include infection and Graft-versus host disease (GVHD) which although not directly related, were the major causes of morbidity and mortality, especially in allogeneic HPCT.

Blood and the lymphatic system disorders:

Myelo-suppression and immuno-suppression were the desired therapeutic effects of the conditioning regimen. Therefore all patients experienced profound cytopenia: leukopenia 96%, thrombocytopenia 94%, and anemia 88%. The median time to neutropenia was 4 days for both autologous and allogeneic patients. The median duration of neutropenia was 6 days and 9 days for autologous and allogeneic patients.

Immune system disorders:

The incidence of acute graft versus host disease (a-GVHD) data was collected in OMC-BUS-4 study (allogeneic)(n=61). A total of 11 patients (18%) experienced a-GVHD. The incidence of a-GVHD grades I-II was 13% (8/61), while the incidence of grade III-IV was 5% (3/61). Acute GVHD was rated as serious in 3 patients. Chronic GVHD (c-GVHD) was reported if serious or the cause of death, and was reported as the cause of death in 3 patients.

Infections and infestations:

39% of patients (40/103) experienced one or more episodes of infection, of which 83% (33/40) were rated as mild or moderate. Pneumonia was fatal in 1% (1/103) and life-threatening in 3% of patients. Other infections were considered severe in 3% of patients. Fever was reported in 87% of patients and graded as mild/moderate in 84% and severe in 3%. 47% of patients experienced chills which were mild/moderate in 46% and severe in 1%.

Hepato-biliary disorders:

15% of SAEs involved liver toxicity. HVOD is a recognized potential complication of conditioning therapy post-transplant. Six of 103 patients (6%) experienced HVOD. HVOD occurred in: 8.2% (5/61) allogeneic patients (fatal in 2 patients) and 2.5% (1/42) of autologous patients. Elevated bilirubine (n=3) and elevated AST (n=1) were also observed. Two of the above four patients with serious serum hepatotoxicity were among patients with diagnosed HVOD.

Respiratory, thoracic and mediastinal disorders :

One patient experienced a fatal case of acute respiratory distress syndrome with subsequent respiratory failure associated with interstitial pulmonary fibrosis in the Busilvex studies.

In addition the literature review reports alterations of cornea and lens of the eye with oral busulfan.

Adverse reactions reported as more than an isolated case are listed below, by system organ class and by frequency. Frequencies are defined as: very common (> 1/10), common (> l/100,< 1/10), uncommon (> 1/1,000, < 1/100).

System organ class	Very common	Common	Uncommon
Blood and lymphatic system disorders	Neutropenia Thrombocytopenia Anaemia Pancytopenia Febrile neutropenia		
Nervous system disorders	Insomnia Anxiety Dizziness Depression	Confusion	Delirium Nervousness Hallucination Agitation Encephalopathy Cerebral haemorrhage Seizure
Metabolism And nutrition disorders	Hyperglycaemia Hypomagnesaemia Hypokalemia Hypocalcaemia Hypophosphatemia Oedema	Hyponatremia	
Cardio vascular disorders	Tachycardia Hypertension Hypotension Vasodilatation Thrombosis	Arrhythmia Atrial fibrillation Cardiomegaly Pericardial effusion Pericarditis Decrease ejection fraction	Femoral artery thrombosis Ventricular extrasystoles Bradycardia Capillary leak syndrome
Respiratory thoracic and mediastinal disorders	Dyspnoea Rhinitis Pharyngitis Cough Hiccup Epistaxis Abnormal breath sounds	Hyperventilation Respiratory failure Alveolar haemorrhages Asthma Atelectasis Pleural effusion	Hypoxia
Gastrointestinal disorders	Nausea Stomatitis Vomiting Anorexia Diarrhoea Constipation Dyspepsia Anus discomfort	Oesophagitis Ileus Haematemesis	Gastrointestinal haemorrhage
Hepato-biliary disorders	Hyperbilirubinaemia Jaundice, increased hepatic enzymes, blood alkaline phosphatase increased	Hepatomegaly	
Skin and subcutaneous tissue disorders	Rash Pruritis Alopecia		
Musculoskeletal connective tissue and bone disorders	Back pain Myalgia Arthralgia		
Renal and urinary disorders	Creatinine elevated Dysuria Oligurea	Bun increase Haematuria Moderate renal insufficiency	

System organ class	Very common	Common	Uncommon
General disorders and administration site conditions	Weight increase Fever Headache Abdominal pain Asthenia Chills Pain Allergic reaction Oedema general Pain or inflammation at injection site Chest pain		

4.9 Overdose

The principal toxic effect is profound myeloablation and pancytopenia but the central nervous system, liver, lungs, and gastrointestinal tract may also be affected.
There is no known antidote to Busilvex other than haematopoietic progenitor cell transplantation. In the absence of haematopoietic progenitor cell transplantation, the recommended dosage of Busilvex would constitute an overdose of busulfan. The haematologic status should be closely monitored and vigorous supportive measures instituted as medically indicated.
There has been one report that busulfan is dialyzable, thus dialysis should be considered in the case of an overdose. Since, busulfan is metabolized through conjugation with glutathione, administration of glutathione might be considered.
It must be considered that overdose of Busilvex will also increase exposure to DMA. In human the principal toxic effects were hepatotoxicity and central nervous system effects. CNS changes precede any of the more severe side effects. No specific antidote for DMA overdose is known. In case of overdose, management would include general supportive care.

5. PHARMACOLOGICAL PROPERTIES

5.1 Pharmacodynamic properties

Pharmacotherapeutic group: Cytotoxic agents (alkylating agents). ATC code: L01AB01
Busulfan is a potent cytotoxic agent and a bifunctional alkylating agent. In aqueous media, release of the methanesulphonate groups produces carbonium ions which can alkylate DNA, thought to be an important biological mechanism for its cytotoxic effect.
Documentation of the safety and efficacy of Busilvex in combination with cyclophosphamide in the BuCy2 regimen prior to conventional allogeneic and/or autologous HPCT derive from two clinical trials (OMC-BUS-4 and OMC-BUS-3).
Two prospective, single arm, open-label, uncontrolled phase II studies were conducted in patients with haematological disease, the majority of whom had advanced disease.
Diseases included were acute leukemia past first remission, in first or subsequent relapse, in first remission (high risk), or induction failures; chronic melogenous leukemia in chronic or advanced phase; primary refractory or resistant relapsed Hodgkin's disease or non-Hodgkin's lymphoma, and myelodysplastic syndrome.
Patients received doses of 0.8 mg/kg busulfan every 6 hours infusion for a total 16 doses followed by cyclophosphamide at 60 mg/kg once per day for two days (BuCy2 regimen).
The primary efficacy parameters in these studies were myeloablation, engraftment, relapse, and survival.
In both studies, all patients received a 16/16 dose regimen of Busilvex. No patients were discontinued from treatment due to adverse reactions related to Busilvex.
All patients experienced a profound myelosuppression. The time to Absolute Neutrophil Count (ANC) greater than 0.5 x 10^6 /I was 13 days (range 9-29 days) in allogenic patients (OMC-BUS 4), and 10 days (range 8-19 days) in autologous patients (OMC-BUS 3). Overall mortality and non- relapse mortality at more than 100 days post-transplant was (8/61) 13% and (6/61) 10% in allotransplanted patients, respectively. During the same period there was no death in autologous recipients.

5.2 Pharmacokinetic properties

The pharmacokinetics of Busilvex has been investigated. The information presented on metabolism and elimination is based on oral busulfan.

Absorption

The pharmacokinetics of i.v. busulfan was studied in 124 evaluable patients following a 2-hour intravenous infusion for a total of 16 doses over four days. Immediate and complete availability of the dose is obtained after intravenous infusion of busulfan. Similar blood exposure was observed when comparing plasma concentrations in patients receiving oral and i.v. busulfan at 1 mg/kg and 0.8 mg/kg respectively. Low inter (CV=21%) and intra (CV=12%) patient variability on drug exposure was demonstrated through a population pharmacokinetic analysis, performed on 102 patients.

Distribution
Terminal volume of distribution V_z ranged between 0.62 and 0.85 I/kg.
Busulfan concentrations in the cerebrospinal fluid are comparable to those in plasma although these concentrations are probably insufficient for anti-neoplastic activity.
Reversible binding to plasma proteins was around 7% while irreversible binding, primarily to albumin, was about 32%.

Metabolism
Busulfan is metabolised mainly through conjugation with glutathione (spontaneous and glutathione-S-transferase mediated). The glutathione conjugate is then further metabolised in the liver by oxidation. None of the metabolites is thought to contribute significantly to either efficacy or toxicity.

Elimination
Total clearance in plasma ranged 2.25 - 2.74 ml/minute/kg. The terminal half-life ranged from 2.8 to 3.9 hours.
Approximately 30% of the administered dose is excreted into the urine over 48 hours with 1% as unchanged drug. Elimination in faeces is negligible. Irreversible protein binding may explain the incomplete recovery. Contribution of long-lasting metabolites is not excluded.

Pharmacokinetic linearity
The dose proportional increase of drug exposure was demonstrated following intravenous busulfan up to 1 mg/kg.

Pharmacokinetic/pharmacodynamic relationships
The literature on busulfan suggests a therapeutic window between 900 and 1500 µMol.minute for AUC. During clinical trials with i.v. busulfan, 90% of patients AUCs were below the upper AUC limit (1500 µMol.minute) and at least 80 % were within the targeted therapeutic window (900-1500 µMol.minute).

Special populations
The effects of renal dysfunction on i.v.. busulfan disposition have not been assessed.
The effects of hepatic dysfunction on i.v. busulfan disposition have not been assessed. Nevertheless the risk of liver toxicity may be increased in this population.
No age effect on busulfan clearance was evidenced from available i.v. busulfan data in patients over 60 years.

5.3 Preclinical safety data
Busulfan is mutagenic and clastogenic. Busulfan was mutagenic in *Salmonella typhimurium, Drosophila melanogaster* and barley. Busulfan induced chromosomal aberrations *in vitro* (rodent and human cell) and *in vivo* (rodents and humans). Various chromosome aberrations have been observed in cells from patients receiving oral busulfan.
Busulfan belongs to a class of substances which are potentially carcinogenic based on their mechanism of action. On the basis of human data, busulfan has been classified by the IARC as a human carcinogen. WHOhas concluded that there is a causal relationship between busulfan exposure and cancer. The available data in animals support the carcinogenic potential of busulfan. Intravenous administration of busulfan to mice significantly increased the incidences of thymic and ovarian tumours.
Busulfan is teratogen in rats, mice and rabbits. Malformations and anomalies included significant alterations in the musculoskeletal system, body weight gain, and size. In pregnant rats, busulfan produced sterility in both male and female offspring due to the absence of germinal cells in testes and ovaries. Busulfan was shown to cause sterility in rodents. Busulfan depleted oocytes of female rats, and induced sterility in male rats and hamster.
Repeated doses of DMA produced signs of liver toxicity, the first being increases in serum clinical enzymes followed by histopatological changes in the hepatocytes. Higher doses can produce hepatic necrosis and liver damage can be seen following single high exposures.
DMA is teratogenic in rats. Doses of 400 mg/kg/day DMA administered during organogenesis caused significant developmental anomalies. The malformations included serious heart and/or major vessels anomalies: a common truncus arteriosis and no ductus arteriosis, coarctation of the pulmonary trunk and the pulmonary arteries, intraventricular defects of the heart. Other frequent anomalies included cleft palate, anasarca and skeletal anomalies of the vertebrae and ribs. DMA decreases fertility in male and female rodents. A single s.c. dose of 2.2 g/kg administered on gestation day 4 terminated pregnancy in 100% of tested hamster. In rats, a DMA daily dose of 450 mg/kg given to rats for nine days caused inactive spermatogenesis.

6. PHARMACEUTICAL PARTICULARS
6.1 List of excipients
Dimethylacetamide, macrogol 400.

6.2 Incompatibilities

In the absence of compatibility studies, this medicinal product must not be mixed with other medicinal products except those mentioned in 6.6.
Do not use polycarbonate syringes with Busilvex.

6.3 Shelf life

Ampoules:
2 years

Diluted solution
Chemical and physical in-use stability after dilution has been demonstrated for:

- 8 hours (including infusion time) after dilution in glucose 5% or sodium chloride 9 mg/ml (0.9%) solution for injection, stored at 20 °C ± 5 °C
- 12 hours after dilution in sodium chloride 9 mg/ml (0.9%) solution for injection stored at 2 °C-8 °C followed by 3 hours stored at 20 °C ± 5 °C (including infusion time).

From a microbiological point of view, the product should be used immediately after dilution. If not used immediately, in-use storage times and conditions prior to use are the responsibility of the user and would normally not be longer than the above mentioned conditions when dilution has taken place in controlled and validated aseptic conditions.

6.4 Special precautions for storage

Stored at 2 °C-8 °C (in a refrigerator). Do not freeze.

6.5 Nature and contents of container

10 ml of concentrate for solution for infusion in clear glass ampoules (type I).
Pack size: 8 ampoules per box

6.6 Instructions for use and handling and disposal

Preparation of Busilvex
Procedures for proper handling and disposal of anticancer drugs should be considered.
All transfer procedures require strict adherence to aseptic techniques, preferably employing a vertical laminar flow safety hood.
As with other cytotoxic compounds, caution should be exercised in handling and preparing the Busilvex solution:

- The use of gloves and protective clothing is recommended.
- If Busilvex or diluted Busilvex solution contacts the skin or mucosa, wash them thoroughly with water immediately.

Calculation of the quantity of Busilvex to be diluted and of the diluent

Busilvex must be diluted prior to use with either sodium chloride 9 mg/ml (0.9%) solution for injection or glucose solution for injection 5% .

The quantity of the diluent must be 10 times the volume of Busilvex ensuring the final concentration of busulfan remains at approximately 0.5 mg/ml. By example:

The amount of Busilvex and diluent to be administered would be calculated as follows: for a patient with a Y kg body weight:

- **Quantity of Busilvex:**

$$\frac{Y(kg) x 0.8(mg/kg)}{6\ (mg/ml)} = A \text{ ml of Busilvex to be diluted}$$

Y: body weight of the patient in kg

- **Quantity of diluent:**

(A ml Busilvex) x (10) = B ml of diluent

To prepare the final solution for infusion, add (A) ml of Busilvex to (B) ml of diluent (sodium chloride 9 mg/ml (0.9%) solution for injection or glucose solution for injection 5%)

Preparation of the solution for infusion

- Using sterile transfer techniques, break off the top of the ampoule.
- Using a non polycarbonate syringe fitted with a needle:
  - remove the calculated volume of Busilvex from the ampoule.
  - dispense the contents of the syringe into an intravenous bag (or syringe) which already contains the calculated amount of the selected diluent. Always add Busilvex to the diluent, not the diluent to Busilvex. Do not put Busilvex into an intravenous bag that does not contain sodium chloride 9 mg/ml (0.9%) solution for injection or glucose solution for injection 5%.
- Mix thoroughly by inverting several times

After dilution, 1 ml of solution for infusion contains 0.5 mg of busulfan Diluted Busilvex is a clear colourless solution

Instructions for use

Prior to and following each infusion, flush the indwelling catheter line with approximately 5 ml of sodium chloride 9 mg/ml (0.9%) solution for injection or glucose (5%) solution for injection.

Do not flush residual drug in the administration tubing as rapid infusion of Busilvex has not been tested and is not recommended.

The entire prescribed Busilvex dose should be delivered over two hours.

Do not infuse concomitantly with another intravenous solution.

Do not use polycarbonate syringes with Busilvex.

For single use only. Only a clear solution without any particles should be used.

Any unused product or waste should be disposed of in accordance with local requirements for cytotoxic drugs.

7. MARKETING AUTHORISATION HOLDER

Pierre Fabre Medicament
45, Place Abel Gance
F-92654 Boulogne Billancourt Cedex
France

8. MARKETING AUTHORISATION NUMBER(S)

EU/1/03/254/001

9. DATE OF FIRST AUTHORISATION/RENEWAL OF THE AUTHORISATION

09.07.2003

10. DATE OF REVISION OF THE TEXT

26.11.3 CYCLOPHOSPHAMIDE

Nomenclature

Generic name: Cyclophosphamide
Commercial name: Cytoxan, Endoxan, Neosar
Chemical name: 2-[bis(2-chloroethyl)amino] tetrahydro-2H-16,2-oxazaphosphorine 2-oxide monohydrate

Drug class, mechanism of action

Cyclophosphamide is a synthetic antineoplastic drug chemically related to the nitrogen mustards. Cyclophosphamide is an inactive prodrug. Although originally designed to be specifically activated in tumours, it is actually metabolised by specific cytochrome P-450s in the liver by microsomal oxidation to produce 4-hydroxycyclophosphamide (4-HC). 4-HC and its tautomer, aldophosphamide, 4-HC is further metabolised to the non-toxic 4-ketocyclophosphamide. Aldophosphamide decomposes to phosphoramide mustard (PM) and acrolein, the principal active and toxic products, respectively. Cyclophosphamide is biotransformed principally in the liver to active alkylating metabolites by a mined function microsomal oxidase system. These metabolites interfere with the growth of susceptible rapidly proliferating malignant cells. Cytotoxic effect of Cyclophosphamide is cell cycle independent. The mechanism of action is thought to involve cross-linking of tumour cell DNA. Cyclophosphamide is a prototypical alkylator drug.

Form

Cyclophosphamide is a white crystalline powder with the molecular formula $C_7H_{15}C_{12}N_2O_2P \bullet H_2O$ and a molecular weight of 279.1. Cyclophosphamide is soluble in water, saline, or ethanol.
25-mg and 50-mg tablets for oral use; vials of powder of 100, 200, 500, 1,000, and 2,000 mg for intravenous administration.

Storage

Tablets and vials are stored at room temperature and should be discarded after the expiration date. Reconstituted drug is stable for 7 days at room temperature, for at least 14 days at 4^oC, and for more than 15 weeks if frozen. Lyophilised CYTOXAN® contains 75 mg of mannitol per 100 mg of cyclophosphamide (anhydrous) and is supplied in vials for single dose use. Lyophilised CYTOXAN(cyclophosphamide for injection, USP). U.S. Patent No. 4,537,883

NDC 0015-0539-41	100 mg vials, carton of 12, case of 1 carton
NDC 0015-0546-41	200 mg vials, carton of 12, case of 1 carton
NDC 0015-0547-41	500 mg vials, carton of 12, case of 1 carton
NDC 0015-0548-41	1.0 g vials, carton of 6
NDC 0015-0549-41	2.0 g vials, carton of 6

Storage at or below 77°F(25°C) is recommended; this product will withstand brief exposure to temperatures up to 86°F(30°C) but should be protected from temperatures above 86°F(30°C). Procedures for proper handling and disposal of anticancer drugs should be considered. Several guidelines on this subject have been published. There is no general agreement that all of the procedures recommended in the guidelines are necessary or appropriate.

Mixing instructions

Vials of powder, i.e., i.v. preparation, should be dissolved in sterile water for injection, normal saline or bacteriostatic water for injection (paraben preserved only) to provide a concentration of 20 mg per mL. Use the quantity of diluent shown below to reconstitute the product:

**Lyophilised CYTOXAN**	
Dosage Strength	Quantity of Diluent
100 mg	5 mL
200 mg	10 mL
500 mg	20—25 mL
1g	50 mL
2g	80—100 mL

The drug can then be further diluted in 5% dextrose in water (D5W), normal saline, or D5 Ringer's solution. For high doses, normal saline is recommended to minimise the hyponatremia that is associated with SIADH. Once diluted, the i.v. preparation is chemically and physically stable for 24 hours at room temperature or up to 6 days if refrigerated. Reconstituted Lyophilised CYTOXAN does not contain any antimicrobial preservative and thus care must be taken to assure the sterility of prepared solutions. Parenteral drug products should be inspected visually for particulate matter and discoloration prior to administration, whenever solution and container permit.

Solutions of Lyophilised CYTOXAN may be infused intravenously in the following:

- Dextrose Injection, USP (5% dextrose)
- Dextrose and Sodium Chloride Injection, USP (5% dextrose and 0.9% sodium chloride)
- 5% Dextrose and Ringer's Injection
- Lactated Ringer's Injection, USP
- Sodium Chloride Injection, USP (0.45% sodium chloride)
- Sodium Lactate Injection, USP (1/6 molar sodium lactate)

Drug interactions

Concurrent administration of cimetidine may reduce the clearance of active metabolites. Prior treatment with phenobarbital may induce the activity of the specific cyclophosphamide P-450 isoenzymes that are identified as metabolizing cyclophosphamide (CYP2B family and CYP2C9) and enhance the metabolism of cyclophos-phamide, resulting in a shorter half-life for the parent compound. The physician should be alert for possible combined drug actions, desirable or undesirable, involving cyclophosphamide even though cyclophosphamide has been used successfully concurrently with other drugs, including other cytotoxic drugs. Cyclophosphamide treatment, which causes a marked and persistent inhibition of cholinesterase activity, potentiates the effect of succinylcholine chloride. If a patient has been treated with cyclophosphamide within 10 days of general anaes-thesia, the anaesthesiologist should be alerted. Cyclophosphamide has been reported to potentiate doxorubicin-induced cardiotoxicity.

Metabolism, pharmacokinetics

Cyclophosphamide is well absorbed with an oral bioavailability greater than 75%. Peak serum levels occur ap-proximately 1 hour after administration. Activated by hepatic enzymes and metabolised to inactive forms in the liver as well. Plasma concentrations of cyclophosphamide increase roughly linearly with parenteral dose, ranging from 4 nmol per L after a dose of 1 mg per kg to 500 nmol per L after 60 mg per kg. However, at the high doses used in BMT (2,400 mg per m2 or 60 mg per kg), many patients exhibit Michaelis-Menten kinetics. The un-changed drug has an elimination half-life of 3 to 12 hours. The mean total body clearance rate for conventional doses of cyclophosphamide is 80 mL per minute. The renal clearance of cyclophosphamide is approximately 15 mL per minute. The terminal elimination half-life of cyclophosphamide ranges from 108 to 960 minutes, with a mean of 7 hours in adults. In children, shorter half-lives are reported. Cyclophosphamide appears to induce its own clearance temporarily, with more rapid clearances reported on subsequent days of multiday regimens. After high doses of cyclophosphamide (40 to 60 mg per kg), the overall alkylating activity has been reported as 10 to 80 µmol per L. Several cytotoxic and non-cytotoxic metabolites have been identified in urine and in plasma. Concentrations of metabolites reach a maximum in plasma 1 to 3 hours after an intravenous dose. The apparent terminal elimination half-life may be somewhat longer than for cyclophosphamide itself, with most reporting a mean half-life of approximately 8 hours, although the half-life for the initial tautomer pair of 4-HC/aldophosphamide is 1 to 5 hours. Plasma protein binding of unchanged drug is low but some metabolites are bound to an extent greater than 60%. It has not been demonstrated that any single metabolite is responsible for either the therapeutic or toxic effects of cyclophosphamide. Although elevated levels of metabolites of cyclo-phosphamide have been observed in patients with renal failure, increased clinical toxicity in such patients has not been demonstrated. Full doses of cyclophosphamide can be administered to patients in either renal or hepatic failure. Renal clearance contributes only modestly to the elimination of cyclophosphamide, and hepatic injury may result in decreased, rather than increased, exposure to active metabolites. Patients who are obese (>20% over ideal body weight) may have a decreased clearance of cyclophosphamide.

Excretion

Parent drug and metabolites are excreted in the urine. It is eliminated primarily in the form of metabolites, but from 5 to 25% of the dose is excreted in urine as unchanged drug

Toxicity

Myelosuppression is dose limiting, with leukopenia being most significant. Cyclophosphamide is also a very potent immunosuppressive drug, depleting B-lymphocytes and suppressing T-lymphocyte function. Serious, sometimes fatal, infections may develop in severely immunosuppressed patients. Nausea and vomiting com-monly occur with cyclophosphamide therapy, they are generally delayed for 4 to 8 hours after the administration of cyclophosphamide. Anorexia and, less frequently, abdominal discomfort or pain and diarrhoea may occur. There are isolated reports of haemorrhagic colitis, oral mucosal ulceration and jaundice occurring during ther-apy. Alopecia occurs commonly in patients treated with cyclophosphamide. The hair can be expected to grow back after treatment with the drug or even during continued drug treatment, though it may be different in texture or colour. Pigmentation of the skin and changes in nails can occur occasionally in patients receiving the drug. Haemorrhagic cystitis is common with doses over 2 g/m2 (in up to 40% of those who receive cyclophosphamide during bone marrow transplantation), and appears to be related to metabolites of the parent compound. This complication may be manifest by a range of abnormalities, from microscopic haematuria to grossly bloody urine. Rarely, this condition can be severe and even fatal. Fibrosis of the urinary bladder, sometimes extensive, also may develop with or without accompanying cystitis. Atypical urinary bladder epithelial cells may appear in the urine. These adverse effects appear to depend on the dose of cyclophosphamide and the duration of therapy. A review of 100 patients who developed haemorrhagic cystitis due to cyclophosphamide revealed several points.

Intravenous therapy and treatment in children produced cystitis at lower doses and at lower cumulative doses. These patients had symptoms of gross haematuria (78%), dysuria (45%), and microscopic haematuria (93%). Bladder cancer developed in 5 of the 100 affected patients. Prevention consists of frequent voiding and vigorous hydration. Mesna has been evaluated in prevention of cystitis from cyclophosphamide. A prospective randomised study of forced diuresis versus mesna in bone marrow transplant patients receiving high doses of cyclophosphamide demonstrated significantly less macroscopic haematuria with mesna. No human data exist regarding the influence of mesna on cyclophosphamide activity, but one animal study of intraperitoneal mesna and mafosfamide (an active metabolite of cyclophosphamide) demonstrated decreased systemic toxicity and decreased anti-tumour efficacy. Given biologic and pharmacokinetic variables, this observation may or may not extend to humans. Other experimental measures to protect against cyclophosphamide cystitis have included the administration of reduced glutathione, misoprostol (a synthetic prostaglandin), N-acetylcysteine, prostaglandins, sucralfate, and oral sodium pentosanpolysulfate, a heparin analogue. Treatment of cystitis due to cyclophosphamide involves hydration and withdrawal of the drug. If this is unsuccessful, a large-bore bladder catheter (to prevent clot obstruction) is inserted and saline irrigation is done. Cystoscopy and fulguration may be required next. If haemorrhage persists, continuous silver nitrate irrigation may be attempted. Other agents that may be instilled include □-aminocaproic acid, prostaglandins E2 and F2, and vitamin E. Haemorrhagic ureteritis and renal tubular necrosis have also been reported to occur in patients treated with cyclophosphamide. Other toxicities of high-dose therapy include syndrome of inappropriate antidiuretic hormone secretion or antidiuretic hormone–like excess syndrome (SIADH). This generally occurs in patients who receive very high doses of cyclophosphamide. It consists of decreased urinary output, hyponatremia, and inappropriate urinary osmolality in the face of decreased serum osmolality. This syndrome generally resolves within 24 hours after discontinuation of the drug. If water restriction is required, cystitis may be a significant complication. Altered mental status and seizures due to hyponatremia can occur in patients who receive moderate to high doses of cyclophosphamide and free water (e.g., less than 0.9% saline) because of an antidiuretic effect similar to SIADH. One case of transient nephrogenic diabetes insipidus has been reported after high-dose cyclophosphamide therapy for bone marrow transplantation. There are occasional reports of cardiotoxicity when the drug is given at very high doses, such as those used in preparation for a bone marrow transplant. Toxicity has ranged from minor, transient electrocardiographic changes and asymptomatic elevation of cardiac enzymes at a total dose of 100 mg per kg (2.5–4.0 g per m2) to fatality at high doses. An unusual form of acute cardiac toxicity is associated with high-dose cyclophosphamide administration; when severe, the damage takes the form of a haemorrhagic myocarditis. The process is related to the dose/course (usually 4.5 to 6.0 g per m2) rather than the cumulative dose, and is associated with decreased ejection fractions and mean QRS voltage. Although severe haemorrhagic myocarditis can be fatal, milder presentations may be asymptomatic and reversible. Haemopericardium can occur, secondary to haemorrhagic myocarditis and myocardial necrosis. Pericarditis has been reported independent of any haemopericardium. Treatment is supportive. No residual cardiac abnormalities as evidenced by electrocardiogram or echocardiogram appear to be present in patients surviving episodes of apparent cardiac toxicity associated with high doses of cyclophosphamide. Pulmonary fibrosis is a rare complication, and secondary malignancies are well documented but rare as well.

Indications

FDA-approved for many malignancies and used for even more. Most commonly used for breast carcinoma, non-Hodgkin's lymphoma, ovarian carcinoma, and testicular cancer.

Contraindications

Continued use of cyclophosphamide is contraindicated in patients with severely depressed bone marrow function. Cyclophosphamide is contraindicated in patients who have demonstrated a previous hypersensitivity to it.

Dosing - Administration

Doses range from 50 mg/m2 for 14 days every 28 days to standard intravenous doses of 600 to 2,000 mg/ m2 one to six doses every 21 to 28 days to transplant doses of 40 to 60 mg/kg intravenously for 2 to 4 days. Parenteral cyclophosphamide is normally infused over 15 to 60 minutes. Rapid administration (<5–10 minutes) can cause acute light-headedness, tearing, nausea, and perioral numbness. Because cyclophosphamide is a prodrug, no special precautions to minimise the risk of extravasation are needed for i.v. administration. When the drug is used in high doses, hydration with normal saline is provided concurrently for 24 hours after a dose to minimise bladder toxicity and hyponatremia. Furosemide promotes clearance of free water and can be used to correct hyponatremia if it occurs despite restriction of free water administration. Patients with compromised renal function may show some measurable changes in pharmacokinetic parameters of cyclophosphamide metabolism, but there is no consistent evidence indicating a need for cyclophosphamide dosage modification in patients with renal function impairment. Since cyclophosphamide has been reported to be more toxic in adrenalectomised drugs, adjustment of the doses of both replacement steroids and cyclophosphamide may be necessary for the adrenalectomised patient. Antiemetics should be administered prior to the first dose of Cytoxan and continued on a fixed schedule through administration of cyclophosphamide. The risk of haemorrhagic cystitis can be reduced by maintaining adequate bladder irrigation by parenteral hydration, via an indwelling catheter, or by the use of the thiol precursor mesna. Mesna is administered parenterally.

Warnings – Recommendations - Precautions

The patient's haematological profile (particularly neutrophils and platelets) should be monitored regularly to determine the degree of haematopoietic suppression. Urine should also be examined regularly for red cells, which may precede haemorrhagic cystitis. Second malignancies have developed in some patients treated with cyclophosphamide used alone or in association with other antineoplastic drugs and/or modalities. Most frequently, they have been urinary bladder, myeloproliferative, or lymphoproliferative malignancies. Second malignancies most frequently were detected in patients treated for primary myeloproliferative or lymphoproliferative malignancies or non-malignant disease in which immune processes are believed to be involved pathologically. In some cases, the second malignancy developed several years after cyclophosphamide treatment had been discontinued. Urinary bladder malignancies generally have occurred in patients who previously had haemorrhagic cystitis. Cyclophosphamide interferes with oogenesis and spermatogenesis. It may cause sterility in both sexes. Development of sterility appears to depend on the dose of cyclophosphamide, duration of therapy, and the state of gonadal function at the time of treatment. Cyclophosphamide-induced sterility may be irreversible in some patients. Amenorrhea associated with decreased oestrogen and increased gonadotropin secretion develops in a significant proportion of women treated with cyclophosphamide. Affected patients generally resume regular menses within a few months after cessation of therapy. Girls treated with cyclophosphamide during prepubescence generally develop secondary sexual characteristics normally and have regular menses. Girls treated with cyclophosphamide during prepubescence subsequently have conceived. Men treated with cyclophosphamide may develop oligospermia or azoospermia associated with increased gonadotropin but normal testosterone secretion. Sexual potency and libido are unimpaired in these patients. Boys treated with cyclophosphamide during prepubescence develop secondary sexual characteristics normally, but may have oligospermia or azoospermia and increased gonadotropin secretion. Some degree of testicular atrophy may occur. Cyclophosphamide-induced azoospermia is reversible in some patients, though the reversibility may not occur for several years after cessation of therapy. Men temporarily rendered sterile by cyclophosphamide have subsequently fathered normal children. No specific antidote for cyclophosphamide is known. Overdosage should be managed with supportive measures, including appropriate treatment for any concurrent infection, myelosuppression, or cardiac toxicity should it occur.

26.11.4 CYCLOSPORINE

Nomenclature
Generic name: Cyclosporine A, cyclosporine, ciclosporine
Commercial name:
Sandimmun(e)® Soft Gelatine Capsules (cyclosporine capsules, USP)
Sandimmun(e)® Oral Solution (cyclosporine oral solution, USP)
Sandimmun(e)® Injection (cyclosporine concentrate for injection, USP)
Neoral® Capsules (cyclosporine capsules, USP)
Neoral® Oral Solution (cyclosporine oral solution, USP)
Chemical name: Cyclo{-[4-(E)-but-2-enyl-N,4-dimethyl-L-threonyl]-L-homoalanyl-(N-methylglycyl)-(N-methyl-L-leucyl)-L-valyl-(N-methyl-L-leucyl)-L-alanyl-D-alanyl-(N-methyl-L-leucyl)-(N-methyl-L-leucyl)-(N-methyl-L-valyl)-}
Formula: C62H111N11O12. Molecular weight: 1202.6.

Drug class, mechanism of action
Cyclosporine, the active principle in Sandimmune® (cyclosporine) is a cyclic polypeptide immunosuppressant agent consisting of 11 amino acids. It is produced as a metabolite by the fungus species Beauveria nlyea. Sandimmune® (cyclosporine) is a potent immunosuppressive agent which in animals prolongs survival of allogeneic transplants involving skin, heart, kidney, pancreas, bone marrow, small intestine, and lung. Sandimmune® (cyclosporine) has been demonstrated to suppress some humoral immunity and to a greater extent, cell-mediated reactions such as allograft rejection, delayed hypersensitivity, experimental allergic encephalomyelitis, Freunds adjuvant arthritis, and graft vs. host disease in many animal species for a variety of organs. The exact mechanism of action of Sandimmune® (cyclosporine) is not known. Experimental evidence suggests that the effectiveness of cyclosporine is due to specific and reversible inhibition of immunocompetent lymphocytes in the G0-or G1-phase of the cell cycle. T-lymphocytes are preferentially inhibited. The 1-helper cell is the main target, although the 1-suppressor cell may also be suppressed. Sandimmune® (cyclosporine) also inhibits lymphokine production and release including interleukin-2 or 1-cell growth factor (TCGF). No functional effects on phagocytic (changes in enzyme secretions not altered, chemotactic migration of granulocytes, macrophage migration, carbon clearance in vivo) or tumour cells (growth rate, metastasis) can be detected in animals. Sandimmune® (cyclosporine) does not cause bone marrow suppression in animal models or man.

Form
- **Neoral®** (Cyclosporine) Oral Solution contains 100 mg of cyclosporine per mL.
dl-α-tocopherol, absolute ethanol, propylene glycol, corn oil-mono-di-triglycerides, polyoxyl 40 hydrogenated castor oil. 50-mL amber glass bottles with an aluminium cap and rubber stopper. A dispenser set is also provided.
- **Neoral®** (Cyclosporine) Soft Gelatine Capsules contains 25 mg, 50 mg, and 100 mg cyclosporine.
Capsule content: dl-α-tocopherol, absolute ethanol, propylene glycol, corn oil-mono-di-triglycerides, polyoxyl 40 hydrogenated castor oil. Capsule shell: Iron oxide black (25- and 100-mg capsules), titanium dioxide, glycerol 85%, propylene glycol, gelatin. Blister packs of double-sided aluminium.
- **Sandimmune® soft gelatin capsules** (cyclosporine capsules, USP) are available in 25 mg (NDC 0078-0240-15), 50 mg (NDC 0078-0242-15), and 100 mg (NDC 0078-0241-15) strengths. SandoPak® unit-dose packages of 30 capsules, 3 blister cards of 10 capsules.

Each* pink, branded " 78/240", *25 mg capsule contains:

cyclosporine, USP	25 mg
alcohol, USP dehydrated	max 12.7% by volume

Each* corn-yellow, branded " 78/242", *50 mg capsule contains:

cyclosporine, USP	50 mg
alcohol, USP dehydrated	max 12.7% by volume

Each* dusty rose, branded " 78/241", *100 mg capsule contains:

cyclosporine, USP	100 mg
alcohol, USP dehydrated	max 12.7% by volume

Inactive Ingredients: corn oil, gelatin, glycerol, Labrafil M 2125 CS (polyoxyethylated glycolysed glycerides), red iron oxide (25 mg and 100 mg capsule only), sorbitol, titanium dioxide, yellow iron oxide (50 mg capsule only), and other ingredients.

- **Sandimmune® oral solution** (cyclosporine oral solution, USP, NDC 0078-0110-22) is available in 50 mL bottles.

Each mL contains:

cyclosporine, USP	100 mg
alcohol, Ph Helv	12.5% by volume

dissolved in an olive oil, Ph. Helv./Labrafil M 1944 CS (polyoxyethylated oleic glycerides) vehicle which must be further diluted with milk, chocolate milk, or orange juice before oral administration. A dosage syringe is provided for dispensing.

- **Sandimmune® injection** (cyclosporine concentrate for injection, USP, NDC 0078-0109-01) is available in a 5 mL sterile ampul for I.V. administration (boxes of 10 ampuls).

Each mL contains:

cyclosporine, USP	50 mg
*Cremophor® EL (polyoxyethylated castor oil)	650 mg
alcohol, Ph Helv	32.9% by volume
nitrogen	qs

which must be diluted further with 0.9% Sodium Chloride Injection or 5% Dextrose Injection before use.
It has molecular formula: $C_{62}H_{111}N_{11}O_{12}$ with a molecular weight of: 1202.63.

Storage

For Sandimmune capsule

In the original unit-dose container at temperatures below 86°F (30°C) and protected from light. An odour may be detected upon opening the unitdose container, which will dissipate shortly thereafter. This odour does not affect the quality of the product.

For Sandimmune solution or injection

In the original container at temperatures below 86°F (30°C) and protected from light. Do not store in the refrigerator. Protect from freezing. Once opened, the contents must be used within 2 months.

For Neoral capsules

Store at room temperature not exceeding 25ºC. Occasional increases in temperatures up to 30ºC do not affect the quality of the product. Neoral capsules should be left in the blister pack until required for use. When a blister is opened, a characteristic smell is noticeable. This is normal and does not mean that there is anything wrong with the capsule.

For Neoral oral solution

The solution should be used within 2 months of opening the bottle and be stored between 15 and 30ºC, preferably not below 20ºC for prolonged periods, as it contains oily components of natural origin which tend to solidify at low temperatures. A jelly-like formation may occur below 20ºC, which is however reversible at temperatures up to 30ºC. Minor flakes or a slight sediment may still be observed. These phenomena do not affect the efficacy and safety of the product, and the dosing by means of the pipette remains accurate.

Mixing instructions

Immediately before use, the I.V. concentrate should be diluted 1 mL Sandimmune® injection (cyclosporine concentrate for injection, USP) in 20 mL-100 mL 0.9% Sodium Chloride Injection or 5% Dextrose Injection and given in a slow intravenous infusion over approximately 2-6 hours. Diluted infusion solutions should be discarded after 24 hours. Cyclosporine is stable over 72 hours following dilution in glucose 5% and storage at room temperature in the dark. Dilutions in sodium chloride 0.9% are considered to be stable only for 8 hours. In all cases, miscibility in the diluent is poor and vigorous shaking is required after addition to produce even distribution of cyclosporine. The Cremophor® EL (polyoxyethylated castor oil) contained in the concentrate for intravenous infusion can cause phthalate stripping from PVC. Parenteral drug products should be inspected visually for particulate matter and discoloration prior to administration, whenever solution and container permit.

Incompatibilities

PVC, plasticiser diethylhexyl phthalate – a potential carcinogen – was leached from PVC containers by cyclosporine preparations. Such preparations should not be given through PVC tubing nor stored in PVC containers.

Drug interactions

All of the individual drugs cited below are well substantiated to interact with Sandimmune® (cyclosporine).

Drugs that exhibit nephrotoxic synergy

gentamicin<br>tobramycin	amphotericin B	cimetidine	trimethoprim
enalapril	Ketoconazole	ranitidine	sulfamethoxazole
vancomycin	Melphalan	diclofenac	azapropazon

Careful monitoring of renal function should be practiced when Sandimmune® (cyclosporine) is used with nephrotoxic drugs. In general, because of the known potential of NSAIDSs to adversely affect renal function care is advised if these drugs are added to cyclosporine therapy.

Drugs that alter cyclosporine levels

The liver extensively metabolises cyclosporine. Therefore, drugs that affect hepatic microsomal enzymes, particularly the cytochrome P-450 system, may influence circulating cyclosporine levels. Substances known to inhibit these enzymes will decrease hepatic metabolism and increase cyclosporine levels. Substances that are inducers of cytochrome P-450 activity will increase hepatic metabolism and decrease cyclosporine levels. Monitoring of circulating cyclosporine levels and appropriate Sandimmune® (cyclosporine) dosage adjustment are essential when these drugs are used concomitantly. (See Blood Level Monitoring)

Drugs that increase cyclosporine levels

fluoxetine	ketoconazole fluconazole itraconazole	danazol	Erythromycin Clarithromycin Roxithromycin Pristinamycin
diltiazem nicardipine verapamil	Grape fruit (P.O.)	bromocriptine	methylprednisolone
amiodarone clonidine		metoclopramide	Cimetidine ?

Drugs that decrease cyclosporine levels

rifampicin trimethoprim IV	phenytoin	Phenobarbital carbamazepine	Octreotide

Other drug interactions

Reduced clearance of prednisolone, digoxin, and lovastatin has been observed when these drugs are administered with Sandimmune® (cyclosporine). In addition, a decrease in the apparent volume of distribution of digoxin has been reported after Sandimmune® (cyclosporine) administration. Severe digitalis toxicity has been seen within days of starting cyclosporine in several patients taking digoxin. Sandimmune® (cyclosporine) should not be used with potassium-sparing diuretics because hyperkalcemia can occur. Warafrin and cyclosporine interfere both with each other. During treatment with Sandimmune® (cyclosporine), vaccination may be less effective; and the use of live vaccines should be avoided. Myositis has occurred with concomitant lovastatin, frequent gingival hyperplasia with nifedipine, and convulsions with high dose methylprednisolone. Further information on drugs that have been reported to interact with Sandimmune® (cyclosporine) is available from Sandoz Pharmaceuticals Corporation.

Metabolism, pharmacokinetics

The absorption of cyclosporine from the gastrointestinal tract is incomplete and variable. Peak concentrations (Cmax) in blood and plasma are achieved at about 3.5 hours. Cmax and area under the plasma or blood concentration/time curve (AUC) increase with the administered dose; for blood the relationship is curvilinear (parabolic) between 0 and 1400 mg. As determined by a specific assay, Cmax is approximately 1.0 ng/mL/mg of dose for plasma and 2.7-1.4 ng/mL/mg of dose for blood (for low to high doses). Compared to an intravenous infusion, the absolute bioavailability of the oral solution is approximately 30% based upon the results in 2 patients. The bioavailability of Sandimmune® soft gelatin capsules (cyclosporine capsules, USP) is equivalent to Sandimmune® oral solution, (cyclosporine oral solution, USP). Cyclosporine is distributed largely outside the blood volume. In blood the distribution is concentration dependent. Approximately 33%-47% is in plasma, 4%-9% in lymphocytes, 5%-12% in granulocytes, and 41%-58% in erythrocytes. At high concentrations, the uptake by leukocytes and erythrocytes becomes saturated. In plasma, approximately 90% is bound to proteins, primarily lipoproteins. The disposition of cyclosporine from blood is biphasic with a terminal half-life of approximately 19 hours (range: 10-27 hours). Cyclosporine is extensively metabolised but there is no major metabolic pathway. Of 15 metabolites characterised in human urine, 9 have been assigned structures. The major pathways consist of hydroxylation of the C-[illegible]-carbon of 2 of the leucine residues, C1-carbon hydroxylation, and cyclic ether formation (with oxidation of the double bond) in the side chain of the amino acid 3-hydroxyl-N,4-dimethyl-L-2-amino-6-octenoic acid and N-demethylation of N-methyl leucine residues. Hydrolysis of the cyclic peptide chain or conjugation of the aforementioned metabolites does not appear to be important biotransformation pathways. Neoral is a new pharmaceutical form of the active ingredient cyclosporine based on the microemulsion preconcentrate principle. The formation of the microemulsion itself takes place in the presence of water, either in the form of a beverage or in the form of the gastric fluid. When Neoral is given, it provides improved dose linearity in cyclosporine exposure (AUCB), a more consistent absorption profile, and less influence from concomitant food intake and from diurnal rhythm than does Sandimmun. These properties combined yield a lower within-patient variability in pharmacokinetics of cyclosporine, and a stronger correlation between trough concentration and total exposure (AUCB). As a consequence of these additional advantages, the time schedule of Neoral administration

need no longer take that of meals into account. In addition, Neoral produces a more uniform exposure to cyclosporine throughout the day, and from day to day on a maintenance regimen. Neoral soft gelatin capsules and Neoral oral solution are bioequivalent. Compared to Sandimmun (with which peak blood concentrations are achieved within 1-6 hours), Neoral is more quickly absorbed (resulting in a 1 hour earlier mean tmax and a 59% higher mean Cmax), and exhibits, on average, a 29% higher bioavailability.

Excretion

Elimination is primarily biliary with only 6% of the dose excreted in the urine. Only 0.1% of the dose is excreted in the urine as unchanged drug.

Toxicity

The principal adverse reactions of Sandimmune® (cyclosporine) therapy are renal dysfunction, tremor, hirsutism, hypertension, and gum hyperplasia.

Hypertension, which is usually mild to moderate, may occur in approximately 50% of patients following renal transplantation and in most cardiac transplant patients. Hypomagnesaemia has been reported in some, but not all, patients exhibiting convulsions while on cyclosporine therapy. Although magnesium-depletion studies in normal subjects suggest that hypomagnesaemia is associated with neurological disorders, multiple factors, including hypertension, high dose methylprednisolone, hypocholesterolemia, and nephrotoxicity associated with high plasma concentrations of cyclosporine appear to be related to the neurological manifestations of cyclosporine toxicity. The following reactions occurred in 2% or less of patients: allergic reactions, anaemia, anorexia, confusion, conjunctivitis, oedema, fever, brittle fingernails, gastritis, hearing loss, hiccups, hyperglycaemia, muscle pain, peptic ulcer, thrombocytopenia, and tinnitus. The following reactions occurred rarely: impaired glucose tolerance, hyperuricaemia, anxiety, chest pain, constipation, depression, hair breaking, haematuria, joint pain, lethargy, mouth sores, myocardial infarction, night sweats, pancreatitis, pruritus, swallowing difficulty, tingling, upper GI bleeding, visual disturbance, weakness, weight loss, alopecia (sometimes irreversible: alopecia universalis). Gingival hyperplasia occurred in about 30% of recipients. The hypothesis is that reactive metabolites whose formation is catalyzed by cytochrome p450 in gingival cells, induces an inflammatory response and fibrotic proliferation by causing cellular injury. The concomitant administration of nifedipine may produce very much greater enlargement. Enlargement is usually reversible following drug reduction. Improvement has been observed after a course of metronidazole. It is not unusual for serum creatinine and BUN levels to be elevated during Sandimmune® (cyclosporine) therapy. Nephrotoxicity has been noted in 25% of cases of renal transplantation, 38% of cases of cardiac transplantation, and 37% of cases of liver transplantation. Mild nephrotoxicity was generally noted 2-3 months after transplant and consisted of an arrest in the fall of the preoperative elevations of BUN and creatinine at a range of 35-45 mg/dl and 2.0-2.5 mg/dl respectively. This form of nephrotoxicity is usually responsive to Sandimmune® (cyclosporine) dosage reduction. A number of parameters have been significantly associated to nephrotoxicity: donor > 50 years, hypotensive donor, concomitant nephrotoxic drugs, CyA serum through level > 200 ng/mL, gradual rise in Cr (<0.15 mg/dL/day), Cr plateau < 25% above baseline, BUN / Cr 20. Serial deterioration in renal function and morphologic changes in the kidneys characterise a form of chronic progressive cyclosporine-associated nephrotoxicity. From 5%-15% of transplant recipients will fail to show a reduction in a rising serum creatinine despite a decrease or discontinuation of cyclosporine therapy. Renal biopsies from these patients will demonstrate an interstitial fibrosis with tubular atrophy. In addition, toxic tubulopathy, peritubular capillary congestion, arteriolopathy, and a striped form of interstitial fibrosis with tubular atrophy may be present. Though none of these morphologic changes is entirely specific, a histological diagnosis of chronic progressive cyclosporine-associated nephrotoxicity requires evidence of these. When considering the development of chronic nephrotoxicity it is noteworthy that several authors have reported an association between the appearance of interstitial fibrosis and higher cumulative doses or persistently high circulating trough levels of cyclosporine. This is particularly true during the first 6 post transplant months when the dosage tends to be highest. The reversibility of interstitial fibrosis and its correlation to renal function have not yet been determined. Occasionally patients have developed a syndrome of thrombocytopenia and microangiopathic haemolytic anaemia. The vasculopathy is accompanied by avid platelet consumption within the graft as demonstrated by Indium 111 labelled platelet studies. Neither the pathogenesis nor the management of this syndrome is clear. Though resolution has occurred after reduction or discontinuation of Sandimmune® (cyclosporine) and 1) administration of streptokinase and heparin or 2) plasmapheresis, this appears to depend upon early detection with Indium 111 labelled platelet scans. Significant hyperkalcemia (sometimes associated with hyperchloremic metabolic acidosis) and hyperuricaemia have been seen occasionally in individual patients. Hepatotoxicity has been noted in 4% of cases of renal transplantation, 7% of cases of cardiac transplantation, and 4% of cases of liver transplantation. Elevations of hepatic enzymes and bilirubin can be noted usually during the first month of therapy when high doses of Sandimmune® (cyclosporine) are used. The chemistry elevations usually decreased with a reduction in dosage. The clinical spectrum of cyclosporine neurotoxicity includes blurred vision and retinopathy. A causal relationship between the use of cyclosporine and the development of bilateral optic disc oedema has been suggested by some studies. Cortical blindness, a rare complication, has been reported in several patients. Magnetic resonance imaging of affected patients demonstrated either diffuse white matter high signals or focal cortical and white matter lesions, reversible in all cases. Electroencephalograms showed non-specific slowing and dysrhythmia, but no epileptiform activity, and the spinal fluid analysis was

normal. The mechanisms of cyclosporine toxicity manifested as cortical blindness, and the reason for the selective vulnerability of the occipital cortex is unknown. There have been reports of convulsions in adult and paediatric patients receiving cyclosporine, particularly in combination with high dose methylprednisolone. Cremophor® EL (polyoxyethylated castor oil) is known to cause hyperlipemia and electrophoretic abnormalities of lipo-proteins. These effects are reversible upon discontinuation of treatment but are usually not a reason to stop treatment. Rarely (approximately 1 in 1000), patients receiving Sandimmune® injection (cyclosporine concentrate for injection, USP) have experienced anaphylactic reactions. Although the exact cause of these reactions is unknown, it is believed to be due to the Cremophor® EL (polyoxyethylated castor oil) used as the vehicle for the I.V. formulation. These reactions have consisted of flushing of the face and upper thorax, acute respiratory distress with dyspnoea and wheezing, blood pressure changes, and tachycardia. One patient died after respiratory arrest and aspiration pneumonia. In some cases, the reaction subsided after the infusion was stopped. An increased incidence of malignancy is a recognised complication of immunosuppression in recipients of organ transplants. The most common forms of neoplasms are non-Hodgkin's lymphoma and carcinomas of the skin. The risk of malignancies in cyclosporine recipients is higher than in the normal, healthy population but similar to that in patients receiving other immunosuppressive therapies. It has been reported that reduction or discontinuance of immunosuppression may cause the lesions to regress.

Indications

Sandimmune® (cyclosporine) is indicated for the prophylaxis of organ rejection in kidney, liver, and heart allogeneic transplants. The drug may also be used in the treatment of chronic rejection in patients previously treated with other immunosuppressive agents. Cyclosporine is also used in the prevention of graft rejection and in the prevention or treatment of graft-versus-host disease (GvHD) following bone marrow transplantation.

Contraindications

Sandimmune® injection (cyclosporine concentrate for injection, USP) is contraindicated in patients with a hypersensitivity to Sandimmune® (cyclosporine) and/or Cremophor® EL (polyoxyethylated castor oil). Hypersensitivity to cyclosporine.

Dosing - Administration

Sandimmune® injection (cyclosporine concentrate for injection, USP) is usually started 72-24 hours prior to transplantation at an initial dose of 1 to 3 mg / kg slow intravenous infusion over 2 to 6 hours b.i.d. This daily dose (2 to 6 mg / kg /day) is continued until the patient can tolerate the soft gelatin capsules or oral solution. Patients unable to take Sandimmune® soft gelatin capsules or oral solution post-transplantation may be treated with the I.V. concentrate. Sandimmune® injection (cyclosporine concentrate for injection, USP) is administered at 1/3 the oral dose. Patients should be switched to Neoral® or Sandimmune® soft gelatin capsules or oral solution as soon as possible after transplantation. In paediatric usage, the same dose and dosing regimen may be used, although children have required and tolerated higher doses than those used in adults. Sandimmune® soft gelatin capsules (cyclosporine capsules, USP) and Sandimmune® oral solution (cyclosporine oral solution, USP) have decreased bioavailability in comparison to Neoral® soft gelatin capsules (cyclosporine capsules for microemulsion) and Neoral® oral solution (cyclosporine oral solution for microemulsion). Sandimmune® and Neoral® are not bioequivalent and cannot be used interchangeably without physician supervision. The daily doses of Neoral should always be given in 2 divided doses. The available data indicate that after a 1:1 conversion from Sandimmun to Neoral, the trough concentrations of cyclosporine in whole blood are comparable. In many patients, however, higher peak concentrations (Cmax) and an increased exposure to the drug (AUC) may occur. In a small percentage of patients these changes are more marked and may be of clinical significance. Their magnitude depends largely on the individual variance in the absorption of cyclosporine from the originally used Sandimmun, which is known to be highly variable in its bioavailability. Patients with variable trough levels or very high doses of Sandimmun may be poor or inconsistent absorbers of cyclosporine (e.g. patients with cystic fibrosis, liver transplant patients with cholestasis or poor bile secretion, children or some kidney transplant recipients) who may, on conversion to Neoral, become good absorbers. Therefore, in this population, the increase in bioavailability of cyclosporine following a 1:1 conversion from Sandimmun to Neoral might be greater than usually observed. The dose of Neoral should therefore be down titrated individually according to their target trough level range. It needs to be emphasised that the absorption of cyclosporine from Neoral is less variable and the correlation between cyclosporine trough concentrations and exposure (in terms of AUC) is much stronger than with Sandimmun. This makes cyclosporine blood trough concentrations a more robust and reliable parameter for therapeutic drug monitoring. The recommended daily doses is about 12.5 mg/kg/day but should be adapted to renal tolerance and blood level monitoring. To make Sandimmune® oral solution (cyclosporine oral solution, USP) more palatable, the oral solution may be diluted with milk, chocolate milk, or orange juice preferably (avoid grape fruit juice) at room temperature. Patients should avoid switching diluents frequently. Sandimmune® soft gelatin capsules and oral solution should be administered on a consistent schedule with regard to time of day and relation to meals. Take the prescribed amount of Sandimmune® (cyclosporine) from the container using the dosage syringe supplied after removal of the protective cover, and transfer the solution to a glass of milk, chocolate milk, or orange juice. Stir well and drink at once. Do not allow to stand before drinking. It is best to use a glass container and rinse it with more diluent to ensure that the total dose is taken. After use, replace the dosage syringe in the protective cover. Do not rinse the dosage syringe with water or other

cleaning agents either before or after use. If the dosage syringe requires cleaning, it must be completely dry before resuming use. Introduction of water into the product by any means will cause variation in dose. Several study centers have found blood level monitoring of cyclosporine useful in patient management. Dosage is usually adjusted to achieve specific whole blood 24-hour trough levels of 100-300 ng / mL. The above levels are specific to the parent cyclosporine molecule and correlate directly to the new monoclonal specific radioimmunoassays (mRIA-sp). If plasma specimens are employed, levels will vary with the temperature at the time of separation from whole blood. Plasma levels may range from 1/2-1/5 of whole blood levels. Blood level monitoring is not a replacement for renal function monitoring or tissue biopsies.

Warnings

Regular monitoring of renal and liver functions, blood pressure, and serum electrolytes is required in patients receiving cyclosporine. Monitoring of drug concentrations is mandatory in transplanted patients. Impaired renal function at any time requires close monitoring, and frequent dosage adjustment may be indicated. In patients with persistent high elevations of BUN and creatinine who are unresponsive to dosage adjustments, consideration should be given to switching to other immunosuppressive therapy. As in patients receiving other immunosuppressants, those patients receiving Sandimmune® (cyclosporine) are at increased risk for development of lymphomas and other malignancies, particularly those of the skin. The increased risk appears related to the intensity and duration of immunosuppression rather than to the use of specific agents. Because patients receiving Sandimmune® injection (cyclosporine concentrate for injection, USP) can experience anaphylactic reactions, they should be under continuous observation for at least the first 30 minutes following the start of the infusion and at frequent intervals thereafter. If anaphylaxis occurs, the infusion should be stopped. An aqueous solution of epinephrine 1:1000 should be available at the bedside as well as a source of oxygen. Anaphylactic reactions have not been reported with the soft gelatin capsules or oral solution, which lack Cremophor® EL (polyoxyethylated castor oil). In fact, patients experiencing anaphylactic reactions have been treated subsequently with the soft gelatin capsules or oral solution without incident. Care should be taken in using Sandimmune® (cyclosporine) with nephrotoxic drugs. Because Sandimmune® is not bioequivalent to Neoral®, conversion from Neoral® to Sandimmune® using a 1:1 ratio (mg/kg/day) may result in a lower cyclosporine blood concentration. Conversion from Neoral® to Sandimmune® should be made with increased blood concentration monitoring to avoid the potential of underdosing. Patients with malabsorption may have difficulty in achieving therapeutic levels with Sandimmune® soft gelatin capsules or oral solution. Hypertension is a common side effect of Sandimmune® (cyclosporine) therapy. Mild or moderate hypertension is more frequently encountered than severe hypertension and the incidence decreases over time. Antihypertensive therapy may be required. Control of blood pressure can be accomplished with any of the common anti-hypertensive agents. However, since cyclosporine may cause hyperkalcemia, potassium-sparing diuretics should not be used. While calcium antagonists can be effective agents in treating cyclosporine-associated hypertension, care should be taken since interference with cyclosporine metabolism may require a dosage adjustment. During treatment with Sandimmune® (cyclosporine), vaccination may be less effective; and the use of live attenuated vaccines should be avoided. There is a minimal experience with overdosage. Anxiety, burning sensations in the mouth and the extremities, dysgueusia, facial flushing, gastro-intestinal disturbances - diarrhoea and vomiting - , perspiration, increased blood pressure and weak and irregular pulse (atrial fibrillation) as well as mild to moderate renal insufficiency have been reported Because of the slow absorption of Sandimmune® soft gelatin capsules or oral solution, forced emesis would be of value up to 2 hours after administration. Transient hepatotoxicity and nephrotoxicity may occur which should resolve following drug withdrawal. General supportive measures and symptomatic treatment should be followed in all cases of overdosage. Sandimmune® (cyclosporine) is not dialyzable to any great extent, nor is it cleared well by charcoal haemoperfusion.

26.11.5 FLUDARABINE

Nomenclature
Generic name: Fludarabine mono-phosphate
Commercial name: Fludara
Chemical name: 9H-Purin-6-amine, 2-fluoro-9-(5-O-phosphono-beta-D-arabinofuranosyl)
The molecular formula of fludarabine phosphate is C10H16FN5O7P (MW 365.2).

Drug class, mechanism of action
Fludarabine phosphate is a fluorinated purine nucleotide analog of the antiviral agent vidarabine, 9-beta-D-arabinofuranosyladenine (ara-A) that is relatively resistant to deamination by adenosine deaminase. Fludarabine phosphate is rapidly dephosphorylated to 2-fluoro-ara-A and then phosphorylated intracellularly by deoxy-cytidine kinase to the active triphosphate, 2-fluoro-ara-ATP. This metabolite appears to act by inhibiting DNA polymerase alpha, ribonucleotide reductase and DNA primase, thus inhibiting DNA synthesis (only partially cell cycle dependent). The mechanism of action of this antimetabolite is not completely characterised and may be multi-faceted.

Form
Each vial of sterile white lyophilised solid cake contains 50 mg of the active ingredient fludarabine phosphate, 50 mg of mannitol, and sodium hydroxide to adjust pH to 7.7. The pH range for the final product is 7.2-8.2.

Storage
Vials should be refrigerated between 2° -8° C (36° -46° F). Reconstituted drug at concentrations as low as 1 mg/mL are stable for 16 days at room temperature and at 0.04 mg/mL, for 48 hours at room temperature.

Mixing instructions
Fludarabine should be prepared for parenteral use by aseptically adding Sterile Water for Injection USP. When reconstituted with 2 mL of Sterile Water for Injection, USP, the solid cake should fully dissolve in 15 seconds or less; each mL of the resulting solution will contain 25 mg of fludarabine phosphate, 25 mg of mannitol, and sodium hydroxide to adjust the pH to 7.7. The pH range for the final product is 7.2-8.2. In clinical studies, the product has been diluted in 100 cc or 125 cc of 5% Dextrose Injection USP or 0.9% Sodium Chloride USP in order to obtain a final concentration of 0.04 to 1 mg/mL depending on the infusion schedule. Reconstituted Fludarabine contains no antimicrobial preservative and thus should be used within 8 hours of reconstitution. Care must be taken to assure the sterility of prepared solutions. Parenteral drug products should be inspected visually for particulate matter and discoloration prior to administration. Procedures for proper handling and disposal should be considered, according to guidelines issued for cytotoxic drugs. The use of latex gloves and safety glasses is recommended to avoid exposure in case of breakage of the vial or other accidental spillage. If the solution contacts the skin or mucous membranes, wash thoroughly with soap and water; rinse eyes thoroughly with plain water. Avoid exposure by inhalation or by direct contact of the skin or mucous membranes.

Drug interactions
The use of Fludarabine in combination with pentostatin is not recommended due to the risk of severe pulmonary toxicity. Administration with cytarabine may reduce the metabolic activation of Fludarabine but results in increased intracellular concentrations of cytarabine. Concomitant administration of gentamicin might lead to severe ototoxicity (one case).

Metabolism, pharmacokinetics
Fludarabine is available only by the parenteral route. After intravenous administration, the drug is metabolised to 2-fluoro–ara-A and widely distributed in tissues. It has an elimination half-life of 9 to 10 hours. In humans, fludarabine phosphate is rapidly converted to the active metabolite, 2-fluoro-ara-A, within minutes after intravenous infusion. Consequently, clinical pharmacology studies have focused on 2-fluoro-ara-A pharmacokinetics. In a study with 4 patients treated with 25 mg/m2/day for 5 days, the half-life of 2-fluoro-ara-A was approximately 10 hours. The mean total plasma clearance was 8.9 L/hr/m2 and the mean volume of distribution was 98 L/m2. Approximately 23% of the dose was excreted in the urine as unchanged 2-fluoro-ara-A. The mean Cmax after the Day 1 dose was 0.57 mcg / mL and after the Day 5 dose was 0.54 mcg / mL. No information is available on pharmacokinetics parameters, other than Cmax, following the Day 5 dose of 25 mg/m2. Total body clearance of 2-fluoro-ara-A has been shown to be inversely correlated with serum creatinine, suggesting renal elimination of the compound. A correlation was noted between the degree of absolute granulocyte count nadir and increased area under the concentration X time curve (AUC).

Excretion
The drug and metabolite are excreted primarily by the kidneys.

Toxicity
Neurotoxicity, including cortical blindness, confusion, somnolence, coma, and demyelinating lesions, is dose limiting, but the lower doses conventionally used rarely produce these side effects. Objective weakness, agitation, confusion, visual disturbances, and coma have occurred at the recommended dose in CLL patients. Peripheral neuropathy has also been observed. There are clear dose dependent toxic effects seen with FLUDARA FOR INJECTION. Dose levels approximately 4 times greater (96 mg/m2/day for 5 to 7 days) than that recommended for CLL (25 mg/m2/day for 5 days) were associated with a syndrome characterised by delayed blindness, coma and death. Severe neurotoxicity appeared from 21 to 60 days following the last dose in 16

of 36 patients who received Fludarabine at high doses (96 mg/m2/day for 5 to 7 days). This syndrome has been reported rarely in patients treated with doses in the range of the recommended CLL dose of 25 mg/m2/day for 5 days every 28 days. The possible adverse effects on fertility in humans have not been adequately evaluated. At these doses, mild myelosuppression is the most common toxicity, with cumulative lymphopenia being the most clinically important. Myelosuppression may be severe and cumulative. Life threatening and sometimes fatal autoimmune haemolytic anaemias have been reported to occur. Nausea, vomiting, and other GI toxicities are rare. Alopecia and rash are also rare. Other commonly reported events include malaise, fatigue, anorexia, and weakness. Serious opportunistic infections have occurred. Oedema has been frequently reported. Pneumonia, a frequent manifestation of infection in CLL patients, occurred in 16% and 22% of those treated with Fludarabine in the M.A. and SWOG studies, respectively. Rare cases of haemorrhagic cystitis have been reported. Pulmonary hypersensitivity reactions to Fludarabine characterised by dyspnoea, cough and interstitial pulmonary infiltrate have been observed. Tumour lysis syndrome - hyperuricaemia, hyperphosphataemia, hypocalcaemia, metabolic acidosis, hypercalcaemia, haematuria, urate crystalluria, and renal failure - has been reported in CLL patients. Flank pain and haematuria may herald the onset of this syndrome. A number of clinical settings may predispose to increased toxicity, including advanced age, renal insufficiency, and bone marrow impairment. Such patients should be monitored closely for excessive toxicity and the dose modified accordingly.

Indications

FDA-approved for the treatment of chronic lymphocytic leukaemia. Also used for low-grade lymphomas and for acute myeloid leukaemia.

Contraindications

Fludarabine is contraindicated in those patients who are hypersensitive to this drug or its components.

Dosing - Administration

The recommended dose is 25 mg/m2/day administered intravenously over a period of approximately 30 minutes daily for five consecutive days. Prolonged infusions have also been used. Dosage may be decreased or delayed based on evidence of haematological or non-haematologic toxicity. Physicians should consider delaying or discontinuing the drug if neurotoxicity occurs.

Warnings – Recommendations - Precautions

While chemotherapy-induced myelosuppression is often reversible, administration of Fludarabine requires careful haematological monitoring. Instances of life-threatening and sometimes fatal autoimmune haemolytic anaemia have been reported to occur after one or more cycles of treatment with Fludarabine in patients with or without a previous history of autoimmune haemolytic anaemia or a positive Coombs' test and who may or may not be in remission for their disease. Steroids may or may not be effective in controlling these haemolytic episodes. The majority of patients rechallenged with Fludarabine developed a recurrence in the haemolytic process. The mechanism(s), which predispose patients to the development of this complication, has not been identified. Patients undergoing treatment with Fludarabine should be evaluated and closely monitored for haemolysis. Transfusion-associated graft-versus-host disease has been observed rarely after transfusion of non-irradiated blood in Fludarabine treated patients. Consideration should, therefore, be given to the use of irradiated blood products in those patients requiring transfusions while undergoing treatment with Fludarabine. Patients undergoing therapy should be closely observed for signs of haematological and non-haematologic toxicity. Periodic assessment of peripheral blood counts is recommended to detect the development of anaemia, neutropenia and thrombocytopenia. Tumour lysis syndrome associated with Fludarabine treatment has been reported in CLL patients with large tumour burdens. There are inadequate data on dosing of patients with renal insufficiency. Fludarabine must be administered cautiously in patients with renal insufficiency. The total body clearance of 2-fluoro-ara-A is inversely correlated with serum creatinine, suggesting renal elimination of the compound. The safety and effectiveness of Fludarabine in children have not been established. There is no known specific antidote for Fludarabine overdosage. Treatment consists of drug discontinuation and supportive therapy. High doses are associated with severe thrombocytopenia and neutropenia due to bone marrow suppression, delayed blindness, coma, and death.

26.11.6 MESNA

Nomenclature
Generic name: mercapyoethanesulfonate sodium
Commercial name: Mesnex, Uromitexan
Chemical name: sodium-2-mercaptoethane sulfate

Drug class, mechanism of action
Mesna currently is used clinically as the specific chemoprotective agent against bladder toxicity resulting from oxazophosphorine-based alkylating agents, including cyclophosphamide. Mesna inactivates the protein-reactive aldehyde, acrolein metabolite of cyclophosphamide, which accumulates in the urinary bladder and results in dose-limiting urotoxicity. Plasma conversion of mesna to its inactive disulfide metabolite, dimesna, allows for the pre-treatment and simultaneous administration of mesna as a urinary protector for high dose cyclophosphamide. Following renal filtration and secretion, dimesna is converted back to the active parent compound by glutathione reductase, which is subsequently delivered to the bladder. The mesna free sulfydryl groups in the urinary bladder can directly complex to and thus neutralise acrolein, in addition to potentially blocking acrolein formation in the urinary tract. The metabolic characteristic of mesna should preclude any potential protection to tumours. Indeed, there is no clinical evidence that mesna coadministration with ifosfamide results in decreased antitumor activity. However, mesna has been shown to prevent the cytotoxicity of platinum agents when given simultaneously with them in in vitro models.

Form
Aqueous solution at 100 mg/mL.

Storage
Ampules should be stored at room temperature and discarded after listed expiration date. Diluted mesna solutions are stable for at least 24 hours at 4oC. Diluted solutions are stable at room temperature for up to 72 hours depending on the concentration and fluid composition used. The most stable solution is 1 mg/mL in D5/0.45% saline.

Mixing instructions
The 100-mg/mL solution can be used undiluted; more typically it is diluted in any of a number of fluids to concentrations of 1 to 20 mg/mL for rapid or prolonged infusions.

Incompatibilities
Mesna is not compatible in solution with cisplatin.

Drug interactions
Mesna does not decrease the effectiveness of cytotoxic drugs or radiation. However, mesna has been shown to prevent the cytotoxicity of platinum agents when given simultaneously with them in in vitro models.

Metabolism, pharmacokinetics
Mesna has an oral bioavailability of approximately 50% and is usually given by vein. After an intravenous dose, mesna is converted in the plasma to dimesna, is filtered by the kidneys, and is reconverted into mesna in the urine. It has an elimination half-life of 1 hour.

Excretion
As in the preceding paragraph.

Toxicity
Mesna is usually very well tolerated. It has occasionally caused nausea, vomiting, diarrhoea, rash, fatigue, headache, hypotension, and arthralgias.

Indications
FDA-approved for use as a uroprotectant when administering ifosfamide. Also effective for high-dose cyclophosphamide.

Dosing - Administration
Proper scheduling of mesna has been based on pharmacokinetic analysis, which showed that mesna and dimesna have relatively short half-lives of approximately 1 hour and that peak urinary thiol accumulation following IV or oral mesna occurs at 1 and 3 hours, respectively. Because the half-life of mesna is much shorter than that of acrolein, it must be administered beyond the completion of cyclophosphamide. The usual dose of mesna is 60% of the daily milligram amount of the ifosfamide, given three times daily by intravenous bolus before, 4 hours after, and 8 hours after the chemotherapy or as a continuous infusion with a loading dose before the chemotherapy. Mesna may be continued for up to 24 hours after the chemotherapy has been completed. The approved schedule for IV administration of mesna is as a bolus dose (20% of the ifosfamide dose) prior to ifosfamide and two additional doses, 4 and 8 hours after ifosfamide treatment.

26.12 PATIENT'S INFORMATION / WRITTEN CONSENT (GERMAN VERSION)

PATIENTENINFORMATION UND –EINVERSTÄNDNISERKLÄRUNG

Universitätsklinikum Hamburg-Eppendorf

Knochenmarktransplantation

Prof. Dr. med. Dr. h.c. A. R. Zander
Direktor der Einrichtung für Knochenmarktransplantation

Transplantationszentrum
Martinistraße 52
20246 Hamburg
Telefon: (040) 42803-4850/51
Telefax: (040) 42803-3795
bmt@uke.uni-hamburg.de
www.uke.uni-hamburg.de

DOSISREDUZIERTE VERSUS STANDARDKONDITIONIERUNG, GEFOLGT VON ALLOGENER STAMMZELLTRANSPLANTATION, BEI PATIENTEN MIT MYELODYSPLASTISCHEM SYNDROM (MDS) ODER SEKUNDÄRER AKUTER MYELOISCHER LEUKÄMIE (SAML): EINE RANDOMISIERTE PHASE III-STUDIE

Vorname, Name und Geburtsdatum des Patienten bzw. Patientenaufkleber

26.12.1 PATIENTENINFORMATION

Sehr geehrte Patientin, sehr geehrter Patient,

bei Ihnen wurde eine bösartige Erkrankung des blutbildenden Systems festgestellt, und zwar:

☐ Ein so genanntes myelodysplastisches Syndrom (MDS).

☐ Eine sekundäre myeloische Leukämie (sAML).

Durch Medikamente, mit denen die bösartigen Zellen getötet werden (Chemotherapie), kann man den Krankheitsverlauf zwar günstig beeinflussen, die Heilungschancen bei Ihrer Erkrankung *ohne* allogene Stammzelltransplantation sind jedoch als gering (< 10 %) zu betrachten. Mit einer Knochenmark- oder Stammzelltransplantation von einem passenden Spender ist mit einer Heilung, je nach Krankheitsstadium, bei zwischen 30 % und 60 % der Fälle zu rechnen. Auf Grund der mit dieser Therapieform verbundenen Risiken kommt es, je nach Alter und Krankheitsstadium, in circa 30 - 40 % der Fälle zu therapiebedingten Komplikationen, die tödlich sind. Aus diesem Grund wird diese Therapieform nur bei Patienten, die in einem gutem Allgemeinzustand und jünger als 60 Jahre sind, durchgeführt.

In den letzten Jahren ist es gelungen, die allogene Knochenmark- oder Stammzelltransplantation etwas risikoärmer durchzuführen, indem die vorgeschaltete Hochdosistherapie (auch "Konditionierung" genannt) in der Dosis reduziert wurde, ohne dass das Anwachsen des neuen Knochenmarks bzw. der Stammzellen dadurch gefährdet wurde. Durch diese geringe Intensität der vorgeschalteten Chemotherapie (auch "dosisreduzierte Konditionierung" genannt) ist das Mortalitätsrisiko (d. h. das Risiko, an therapiebedingten Folgen zu sterben) deutlich gesunken und liegt jetzt bei circa 20 %. Diese vorgeschaltete Chemotherapie ist zwar deutlich weniger intensiv als die herkömmliche Hochdosistherapie (nachfolgend "Standardkonditionierung" genannt), sie ist jedoch stark genug, um sämtliche bösartigen und gutartigen Zellen im Knochenmark zu zerstören. Bisher wurde diese so genannte dosisreduzierte Konditionierung nur bei denjenigen Patienten durchgeführt, die auf Grund des Alters und einer schwerwiegenden Begleiterkrankung nicht für eine Standardkonditionierung in Frage gekommen sind. Nachdem man jedoch gute Erfolge bei dieser Patientengruppe (hohes Alter und Begleiterkrankungen) durch die dosisreduzierte Konditionierung erreicht hatte, soll die vorliegende Studie die Frage beantworten, ob auch bei Patienten in gutem Allgemeinzustand,

denen eine Standardkonditionierung zugemutet werden kann, durch die dosisreduzierte Konditionierung der gleiche Erfolg, aber mit geringerer therapiebedingter Sterblichkeit erreicht werden kann. Aus diesem Grunde bitten wir Sie, an der vorliegenden Therapiestudie teilzunehmen. Das Ziel dieser Studie ist es, nachzuweisen, dass mit der dosisreduzierten Konditionierung im Vergleich zur Standardkonditionierung deutlich weniger tödliche therapiebedingte Komplikationen auftreten, verbunden mit der gleichzeitigen Hoffnung, dass die Rate an Heilungen genauso hoch ist wie nach Standardtherapie. Aus diesem Grunde wird in der vorliegenden Studie, wenn alle Einschlusskriterien erfüllt sind, der Patient nach einem Zufallsprinzip entweder der dosisreduzierten Konditionierung oder der Standardkonditionierung zugeordnet. Damit in beiden Gruppen gleich viele und vergleichbare Patienten auswertbar sind, haben Sie als Patient oder Ihr behandelnder Arzt keinen Einfluß darauf, ob Sie der dosisreduzierten oder der Standardkonditionierung zugeordnet werden.

Gleichgültig, welchem Therapiearm Sie zugeordnet werden, müssen zunächst die Blutstammzellen von Ihrem passenden, gewebeverträglichen Spender gesammelt werden. Anschließend wird bei Ihnen eine Chemotherapie entweder mit Busulfan (12,8 mg/kg Körpergewicht intravenös) und Cyclophosphamid (120 mg/kg Körpergewicht) durchgeführt oder, falls Sie der dosisreduzierten Konditionierung zugeordnet werden, Busulfan (6,4 mg/kg Körpergewicht intravenös) und Fludarabin (150 mg/m²). Falls Ihr Spender ein Fremdspender ist, kann entweder das Anti-Thymozyten-Globulin oder der Antikörper Campath hinzugefügt werden. Diese zusätzliche Medikation hat den Effekt, Ihr Immunsystem so weit zu unterdrücken, damit das transplantierte Knochenmark bzw. die Stammzellen gut anwachsen können. Des Weiteren bewirken beide Substanzen auf Grund ihrer langen Verweildauer im Blut eine Hemmung auf die nach der Transplantation auftretende Spender-gegen-Wirt-Reaktion (siehe auch Seite 88).

Die Therapie wird im Einzelnen wie folgt durchgeführt:

Nachdem die Vorsorgeuntersuchungen, insbesondere von Lungen- und Herzfunktion, keine Einwände gegen die geplante Therapie zeigten, wird Ihnen zunächst stationär in Narkose ein subkutaner Katheter implantiert, über den Sie nun die Chemotherapie und die Immuntherapie erhalten. Beides sind Medikamente, die zum einen Ihre Krankheit bekämpfen und zum anderen Ihr Immunsystem unterdrücken, damit die übertragenen Stammzellen Ihres Spenders besser angenommen und nicht abgestoßen werden; ferner verhindern sie, dass eine schwerwiegende Spender-gegen-Wirt-Reaktion auftreten kann. Die Chemotherapie wird Ihnen in beiden Therapiearmen (d. h. in beiden Therapiegruppen) intravenös verabreicht. Alternativ kann jedoch die Chemotherapie teilweise auch in Tablettenform verabreicht werden.

In **Arm A** erhalten Sie Busulfan (12,8 mg/kg Körpergewicht) und Cyclophosphamid (120 mg/kg Körpergewicht). Die Nebenwirkungen von Busulfan und Cyclophosphamid sind insbesondere Haarverlust (fast 100 %, in seltenen Fällen (5 %) dauerhaft), Übelkeit (circa 90 %), Entzündung der Mundschleimhäute (circa 80 %), Infektanfälligkeit und Fieber durch Abfall der weißen Blutkörperchen und Blutungsneigung durch Abfall der Blutplättchen (80 - 90 %); Hautveränderungen im Sinne von Pigmentationen können in circa 80 % der Fälle unter Busulfan auftreten. Eine hämorrhagische Zystitis (blutige Harnwegsinfektion) tritt unter Cyclophosphamid in weniger als 10 % der Fälle auf. Als Organschäden können (sehr selten) Störungen des Herzens (< 1 %) und (je nach Vorbehandlung) durch Busulfan Schäden der Leber in bis zu 50 % der Fälle auftreten. In der Regel sind Übertragungen von Blutkonserven notwendig, und wenn die Werte der Blutplättchen unter einen bestimmten Schwellenwert fallen, ist die Gabe von Thrombozytenkonzentraten notwendig. Auch erhalten Sie prophylaktisch Medikamente gegen Bakterien, Pilze und Viren, damit es nicht zu einer infektiösen Erkrankung kommt.

Im **Arm B** (dosisreduzierte Konditionierung) sind die Nebenwirkungen von Busulfan ähnlich wie im Standardarm, d. h. Haarverlust (fast 100 %, in seltenen Fällen (5 %) dauerhaft), Übelkeit (circa 60 - 80 %), Entzündung der Mundschleimhäute (circa 80 %), Infektanfälligkeit und Fieber durch Abfall der weißen Blutkörperchen und Blutungsneigung durch Abfall der Blutplättchen (60 - 90 %); Hautveränderungen im Sinne von Pigmentationen können auch nach der reduzierten Busulfan-Dosis in 60 - 80 % der Fälle auftreten. Auch hier sind in der Regel Übertragungen von Blutkonserven notwendig; wenn die Thrombozyten niedriger sind, ist auch die Gabe von Thrombozytenkonzentraten notwendig. Ebenfalls können Schädigungen der Leber auftreten (40 - 50 % der Fälle). Auch in diesem Therapiearm erhalten Sie prophylaktisch Medikamente gegen Bakterien, Pilze und Viren, damit es nicht zu einer infektiösen Erkrankung kommt. Das Medikament Fludarabin bewirkt in seltenen Fällen Übelkeit (< 10 %).

Falls bei Ihnen eine Fremdspendertransplantation durchgeführt werden soll, kann im Rahmen des Protokolls nach Absprache mit Ihren behandelnden Ärzten entweder ein Anti-Thymozyten-Globulin (ATG) oder ein monoklonaler Antikörper namens Campath eingesetzt werden.

Das ATG ist ein vom Kaninchen hergestelltes Serum, welches zu einem Abfall der so genannten T-Zellen führt. Da ATG ein fremdes Eiweiß ist, kann es hier zu Nebenwirkungen wie Fieber (circa 90 %), Schüttelfrost (circa 80 %), Pulsanstieg (circa 90 %), Blutdruckabfall (circa 10 %), sehr selten auch im Sinne einer allergischen Reaktion zu Atemnot oder Hautausschlag (< 5 %) kommen. Dieser Reaktion wird in der Regel durch ein entsprechendes Medikament, nämlich Kortison, vorgebeugt.

Alternativ kann an Stelle des ATG bei Ihnen auch der monoklonale Antikörper Campath-1H eingesetzt werden, der von der Wirkung her ähnlich dem ATG ist; er führt nämlich zu einem Abfall bestimmter Abwehrzellen, so genannter T-Zellen. Ähnlich wie beim ATG kann es hier zu Fieber (circa 85 %), Schüttelfrost (85 %) oder Pulsanstieg (90 %) kommen; in seltenen Fällen kommt es auch zu Blutdruckabfall (20 %) und sehr selten auch zu Atemnot oder allergischen Reaktionen (10 %).

Sowohl unter ATG als auch unter Campath kommt es auf Grund der sehr langen Verweildauer im Blut auch zu einer verzögerten Erholung des Immunsystems nach der allogenen Transplantation, so dass bei beiden Medikamenten die Infektionsgefahr, insbesondere durch Viren, erhöht ist.

Nach Abschluss der Vorbehandlungen werden Ihnen die Stammzellen Ihres Spenders injiziert. In den darauf folgenden 2 – 3 Wochen kann es in beiden Therapiearmen zu den oben beschriebenen Nebenwirkungen kommen. Nach circa zwei Wochen kommt es in beiden Therapiearmen zu einem Anwachsen der Stammzellen.

Auch kann es in beiden Therapiearmen, vermittelt durch die fremden Lymphozyten, zu einer so genannten Transplantat-gegen-Wirt-Reaktion (Spender-gegen-Wirt-Reaktion, auch Graft-versus-Host disease (GvHD) genannt) kommen. Dabei greifen die Abwehrzellen Ihres Spenders unterschiedliche Organe Ihres Körpers – insbesondere Haut, Darm und Leber – an, so dass es zu Hautrötungen, Durchfall und auch zur Entwicklung einer nicht ansteckenden Gelbsucht kommen kann. Diese Reaktion ist jedoch teilweise erwünscht, da diese Abwehrreaktion sich gleichzeitig auch gegen die verbliebenen Tumorzellen richtet. Trotzdem muss diese Abwehrreaktion mit Medikamenten, die das Immunsystem kontrollieren, unterdrückt werden. Hier wird insbesondere Ciclosporin A routinemäßig nach beiden Formen der Transplantation eingesetzt. Dieses Medikament müssen Sie noch nach der Transplantation bis circa zum Tag 180 einnehmen. Da das Medikament potentiell die Nieren schädigen kann, ist für ausreichende Flüssigkeitszufuhr zu sorgen.

Die Produktion Ihres Knochenmarks von weißen Blutkörperchen (Leukozyten), roten Blutkörperchen (Erythrozyten) und Blutplättchen (Thrombozyten) wird noch mehrere Wochen vermindert sein, so dass ein Bedarf für Transfusionen von Erythrozyten und Thrombozyten besteht. Zum schnelleren Anwachsen der Leukozyten erhalten Sie einen Wachstumsfaktor (G-CSF = Granulozyten-stimulierender Faktor). Dieses Medikament wird gentechnologisch hergestellt und ist für diesen Zweck zugelassen. Nachdem die Leukozyten und speziell die Untergruppe der Granulozyten eine sichere Konzentration erreicht haben, werden Sie von der Transplantationseinheit entlassen. Zunächst häufigere, später weniger häufige Besuche in der Ambulanz werden notwendig sein, um die weitere Entwicklung in der Nachtransplantationsphase zu begutachten und die notwendigen Medikamente einzustellen.

Die T-Zellen aus dem Knochenmark bzw. aus dem Stammzellpräparat Ihres Spenders haben nicht nur einen maßgeblichen Einfluß auf die Entwicklung einer Spender-gegen-Wirt-Reaktion (GvHD), sondern sie haben auch einen positiven Einfluß auf die Abtötung der Tumorzellen, so dass ein Teil dieser Zellen Ihnen auch noch später in Form einer Spritze zur Verstärkung dieses Effektes auf die Tumorzellen gegeben werden kann. Auch hier kann es, ähnlich wie zu Beginn der Behandlung, zu Komplikationen im Sinne einer Spender-gegen-Wirt-Reaktion kommen. Diese Lymphozyten bekommen Sie jedoch nur, wenn circa sechs Monate nach Transplantation immer noch eine Resterkrankung bei Ihnen nachweisbar ist oder es bereits vorher zu einem Fortschreiten der Erkrankung bzw. einem nicht-vollständigen Anwachsen der Blutzellen Ihres Spenders gekommen ist.

Zusammenfassend können folgende Nebenwirkungen durch die Behandlung auftreten, wobei die Nebenwirkungshäufigkeit prinzipiell in beiden Therapiearmen gleich ist (in Klammern: Häufigkeit des Auftretens):

- Akute Spender-gegen-Wirt-Reaktion (aGvHD) mit Hautexanthemen, schweren Durchfällen und starker Gelbsucht (circa 30 % bei Familienspender, circa 50 % bei Fremdspendern);
- chronische Spender-gegen-Wirt-Reaktion (cGvHD) mit Hautexanthemen, Muskel- und Gelenkbeteiligung. Schleimhautentzündungen des Mundes und Leberfunktionsstörungen (mildere Fälle bei Familienspendertransplantation: circa 20 %, bei Fremdspendertransplantation circa 30 %; schwerwiegende Verlaufsformen bei Familienspendertransplantation circa 5 %; bei Fremdspendertransplantation circa 10 %);
- Infektionen während der Transplantationsphase (circa 80 %);
- Entzündungen an den Schleimhäuten (Mucositis) des Mundes, des Rachen, der Speiseröhre, des Magens und des Darmes (circa 75 %);
- Blutungen während der akuten Transplantationsphase (Nasenbluten und selten Darmbluten) (< 10 %);
- interstitielle Pneumonien (Lungenentzündungen), die in den ersten drei Monaten nach Transplantation auftreten können (< 10 %);
- Nebenwirkungen des Ciclosporin A, wie z. B. Störungen der Nierenfunktion (< 30 %), Depressionen (< 5 %), Bluthochdruck (< 20 %), Krämpfe (< 10 %), außergewöhnlicher Haarwuchs (circa 40 %) und Übelkeit (circa 10 %);

- Nebenwirkungen des ATG (Antithymozyten-Globulin): Fieber (90 – 100 %), Schüttelfrost (80 – 90 %), allergische Reaktionen (10 %), selten Veränderungen des Blutbildes und der Leberenzyme (< 5 %);
- falls es zu einer Spender-gegen-Wirt-Reaktion kommt, muss das Kortisonpräparat eingesetzt werden; hierbei können insbesondere Muskelschwäche, Verbreiterung des Gesichts, Schwäche der Knochenstruktur mit möglichen Knochenbrüchen oder Diabetes (Zuckerkrankheit) auftreten;
- Sterilität und (bei Frauen) Ausbleiben der Monatsblutung (circa 100 %);
- Veränderung des Hautkolorits unter Busulfan (circa 80 %);
- Verlust des Haupthaares, meist vorübergehend, (90 – 100 %, selten dauerhaft (circa 5 %);
- selten Schädigung des Herzmuskels (insbesondere unter Cyclophosphamid, d. h. = Standardtherapie) (< 1 %);
- sehr selten dauerhafte Schädigung der Nierenfunktion (< 1 %);
- Abstoßungsreaktion des Knochenmark- bzw. Blutstammzelltransplantates; in diesem Falle würde ein zweites Transplantat vom Spender folgen (< 5 %);
- erhöhte Wahrscheinlichkeit, dass in den nächsten 20 Jahren andere Tumoren auftreten können (< 5 %).

Prinzipiell sind die Nebenwirkungen in beiden Therapiearmen ähnlich, wobei in der Standardtherapie auf Grund der Intensität der Chemotherapie die Ausprägung der Nebenwirkungen etwas stärker ist. Trotz der möglichen, heilenden Wirkung der allogenen Stammzelltransplantation kann es auch zu einem Rückfall der Erkrankung kommen. Das Risiko, einen Krankheitsrückfall zu erleiden, liegt je nach Krankheitsstadium zwischen 15 % und 30%. Grundsätzlich ist es möglich, dass bei der niedrigen Intensität der Konditionierungstherapie das Rückfallrisiko etwas höher liegt als nach einer Standardkonditionierung.

Da die Knochenmarktransplantation eine junge Behandlungsform ist, können theoretisch noch Nebenwirkungen auftreten, die bisher noch nicht beschrieben sind. Die möglichen Vorteile der allogenen Blutstammzelltransplantation liegen gegenüber den herkömmlichen Therapien in der größeren Wahrscheinlichkeit, eine langdauernde Remission und eine Heilung der zugrunde liegenden Erkrankung zu erreichen.

Die in der vorliegenden Studie zu untersuchenden Therapien "dosisreduzierte Konditionierung" oder "Standardtherapie" haben, kurz zusammengefasst, folgende Vor- und Nachteile:

Dosisreduzierte Konditionierung:

Vorteil: Voraussichtlich weniger therapiebedingte Nebenwirkungen und niedrigeres Risiko einer therapiebedingten Sterblichkeit.

Nachteil: Auf Grund der niedrigeren Intensität der Therapie eventuell eine höhere Rückfallgefahr.

Standardtherapie:

Vorteil: Wahrscheinlich ist auf Grund der Intensität der Chemotherapie mit weniger Rückfällen nach allogener Transplantation zu rechnen.

Nachteil: Auf Grund der Intensität der Chemotherapie ist mit mehr therapiebedingten Komplikationen, insbesondere einer höheren therapiebedingten Sterblichkeit zu rechnen.

Die vorliegende Therapiestudie soll also zeigen, dass die dosisreduzierte Konditionierung die therapiebedingte Mortalität verringert und damit die Verträglichkeit der allogenen Stammzelltransplantation verbessert. Die Studie wird hoffentlich auch zeigen, dass es nicht zu vermehrten Rückfällen nach der dosisreduzierten Konditionierung im Vergleich zur Standardtherapie kommt.

Sollten Sie sich gegen eine Teilnahme an dieser Studie aussprechen, wird Ihnen von den behandelnden Ärzten die Standardkonditionierung (als die derzeit gültige Standardtherapie in der allogenen Transplantation) empfohlen.

Andere Behandlungsformen (außer der allogenen Stammzelltransplantationen) wurden mit Ihnen diskutiert:

- Verabreichung einer Chemotherapie zur Tumorreduktion (ohne Heilungschancen).
- Bluttransfusionen zur Substitution des Erythrozytenbedarfs oder Stimulation der niedrigen Thrombozyten und Granulozyten. Durch diese Therapieform wird eine Verbesserung der Blutwerte, ein gesichertes verlängertes Überleben ist jedoch bisher nicht nachgewiesen.

Ihre während Blutstammzelltransplantation erhobenen Behandlungsdaten werden pseudoanonym im europäischen und internationalen Bone Marrow Transplantation Register zur Verbesserung der Knochenmarktransplantation gespeichert.

Im Rahmen dieser Studie wurde für Sie eine Versicherung nach § 40, 41 Arzneimittelgesetz abgeschlossen bei

Gerling Industrie Deutschland GmbH, Postfach 13 04 05, 20104 Hamburg
Police-Nr. 70 – 005361379 – 5
Ansprechpartner: Herr Reiner Witoßek
Tel.: 040 – 441 99 – 2 / Fax: 040 – 441 99 - 6

Ihre Teilnahme an der Studie ist freiwillig und kann jederzeit widerrufen werden; allerdings hat ein Abbruch der klinischen Prüfung nach Erhalt der hochdosierten Chemotherapie und vor der Reinfusion der Blutstammzellen mit sehr großer Wahrscheinlichkeit Ihren Tod zur Folge.

Der Versicherungsschutz besteht für studienbedingte Gesundheitsschäden, die innerhalb von fünf Jahren nach Abschluss während der Nachbeobachtungsphase der beim Versicherten durchgeführten klinischen Prüfung eingetreten sind und nicht später als zehn Jahre nach der Beendigung der klinischen Prüfung dem Versicherer gemeldet werden.

26.12.1.1 Obliegenheiten des Versicherten (Auszug aus den Allgemeinen Versicherungsbedingungen für klinische Prüfungen von Arzneimitteln (AVB))

26.12.1.1.1 § 14 II (1) – (6): Obliegenheiten des Versicherten

1) Während der Dauer der klinischen Prüfung darf sich die versicherte Person einer anderen medizinischen Behandlung nur im Einvernehmen mit dem klinischen Prüfer unterziehen. Dies gilt nicht in einem medizinischen Notfall; der klinische Prüfer ist von einer Notfallbehandlung unverzüglich zu unterrichten.

2) Eine Gesundheitsschädigung, die als Folge der klinischen Prüfung eingetreten sein könnte, ist dem Versicherer unverzüglich anzuzeigen.

3) Der Versicherte hat alle zweckmäßigen Maßnahmen zu treffen, die der Aufklärung der Ursache und des Umfangs des eingetretenen Schadens und der Minderung dieses Schadens dienen.

4) Auf Verlangen des Versicherers ist der behandelnde Arzt – solcher gilt auch ein Konsiliararzt oder ein gutachterlich tätiger Arzt – zu veranlassen, einen Bericht über die Gesundheitsschädigung und, nach Abschluss der ärztlichen Behandlung, einen Schlussbericht zu erstatten; außerdem ist dafür Sorge zu tragen, dass alle etwa weiter noch von dem Versicherer geforderten Berichte des behandelnden Arztes geliefert werden.

5) Die behandelnden Ärzte, auch diejenigen, von denen der Versicherte aus anderen Anlässen behandelt oder untersucht worden ist, und die Sozialversicherungsträger sowie andere Versicherer, wenn dort die Gesundheitsschädigung gemeldet ist, sind zu ermächtigen, dem Versicherer auf Verlangen Auskunft zu erteilen.

6) Beim etwaigen Eintritt des Todes ist dies dem Versicherungsunternehmen durch den Rechtsnachfolger [Erben] der Versuchsperson telegraphisch binnen 48 Stunden anzuzeigen.

7) Die Versuchsperson muss alle zweckmäßigen Maßnahmen treffen, die der Aufklärung der Ursache und des Umfanges eines Schadens sowie dessen Minderung dienen.

8) Verletzt die Versuchsperson vorsätzlich oder grob fahrlässig eine nach dem Eintritt des Schadens von ihr zu erfüllende Obliegenheit, ist der Versicherer von der Verpflichtung zur Leistung frei. Bei grob fahrlässiger Verletzung besteht die Leistungspflicht weiter, sofern die Verletzung ohne Einfluss auf die Feststellung des Versicherungsfalles oder des Leistungsumfangs geblieben ist.

26.12.1.2 DATENSCHUTZBESTIMMUNGEN GEMÄß § 40 (1) 2. AMG

Die vom Patienten im Rahmen der klinischen Prüfung bzw. wissenschaftlichen Studie erhobenen personenbezogenen Daten/Krankheitsdaten werden aufgezeichnet und können pseudonymisiert (d. h. ohne Namensnennung) weitergegeben werden

a) an den genannten Auftraggeber der Studie zu ihrer wissenschaftlichen Auswertung sowie

b) an die zuständige Überwachungsbehörde oder die zuständige Bundesoberbehörde zur Überprüfung der ordnungsgemäßen Durchführung der Studie.

Die Verarbeitung und Nutzung der pseudonymisierten Daten erfolgt auf Fragebögen und elektronischen Datenträgern für die Dauer von fünfzehn Jahren.

Außerdem kann ein autorisierter und zur Verschwiegenheit verpflichteter Beauftragter des Auftraggebers oder der zuständigen Überwachungsbehörde bzw. der zuständigen Bundesoberbehörde in die beim Prüfarzt vorhandenen personenbezogenen Daten Einsicht nehmen, soweit dies für die Überprüfung der Studie notwendig ist.

Für den Fall, dass die klinische Prüfung in mehreren Ländern durchgeführt wird, können zur Überprüfung der Studie die aufgezeichneten Patientendaten pseudonymisiert auch an die zuständige ausländische Überwachungsbehörde weitergeleitet werden. Auch kann ein zur Verschwiegenheit verpflichteter Beauftragter der zuständigen ausländischen Überwachungsbehörde in die personenbezogenen Patientendaten Einsicht nehmen, soweit dies für die Überprüfung der Studie notwendig ist.

Universitätsklinikum Hamburg-Eppendorf

Knochenmarktransplantation

Prof. Dr. med. Dr. h.c. A. R. Zander
Direktor der Einrichtung für
Knochenmarktransplantation

Transplantationszentrum
Martinistraße 52
20246 Hamburg
Telefon: (040) 42803-4850/51
Telefax: (040) 42803-3795
bmt@uke.uni-hamburg.de
www.uke.uni-hamburg.de

DOSISREDUZIERTE VERSUS STANDARDKONDITIONIERUNG, GEFOLGT VON ALLOGENER STAMMZELLTRANSPLANTATION, BEI PATIENTEN MIT MYELODYSPLASTISCHEM SYNDROM (MDS) ODER SEKUNDÄRER AKUTER MYELOISCHER LEUKÄMIE (SAML): EINE RANDOMISIERTE PHASE III-STUDIE

**Vorname, Name und Geburtsdatum des Patienten bzw. Patientenaufkleber**

26.12.2 PATIENTENEINVERSTÄNDNISERKLÄRUNG

- Über den Zweck und den Hergang der Knochenmark- bzw. Blutstammzelltransplantation wurde ich von Frau / Herrn Dr. ____________________ informiert und eingehend über Wirksamkeit, Nebenwirkungen sowie Risiken aufgeklärt. Weitere Informationen kann ich jederzeit erfragen. Die Teilnahme an der Studie ist freiwillig und kann jederzeit widerrufen werden; allerdings hat ein Abbruch der klinischen Prüfung nach Erhalt der hochdosierten Chemotherapie und vor der Reinfusion der Blutstammzellen mit sehr großer Wahrscheinlichkeit meinen Tod zur Folge.
- Ich bin mit der im Rahmen der klinischen Prüfung erfolgenden Aufzeichnung von Krankheitsdaten, ihrer Weitergabe zur Überprüfung an den Auftraggeber, an die zuständige Überwachungsbehörde oder die zuständige Bundesoberbehörde und, soweit es sich um personenbezogene Daten handelt, mit deren Einsichtnahme durch Beauftragte des Auftraggebers oder der Behörden einverstanden.
- **Ich bin bereit, die erhobenen Behandlungsdaten pseudoanonym an europäische und internationale Knochenmarktransplantations-Register zur Verbesserung der Knochenmarktransplantation zur Verfügung zu stellen.**
- **Mit den in Abschnitt 26.12.1.2 erläuterten Datenschutzbestimmungen bin ich einverstanden.**

Ich erkläre mich mit der angegebenen Behandlung einverstanden.

____

Ort / Datum (Eigenhändig unterzeichnet: Name, Datum und Unterschrift) Patientin / Patient

____

Ort / Datum (Eigenhändig unterzeichnet: Name, Datum und Unterschrift) Ärztin / Arzt

26.13 PATIENT'S WRITTEN INFORMED CONSENT (ENGLISH VERSION)

The Chronic Leukaemia Working Party

MDS-Subcommittee

Dose-reduced versus standard conditioning followed by allogeneic stem cell transplantation in patients with MDS or sAML: A randomised phase III study

26.13.1 INFORMATION FOR THE PATIENT

a) Introduction

This study is conducted by the MDS subcommittee of the Chronic Leukaemia Working Party of the European Blood and Marrow Stem Cell Transplantation Group in Europe.

The main purpose of this information is to allow you to read and to discuss in full the information explained to you by your treating physician. You can decide in private whether you wish to participate in this protocol after reading this information.

You were diagnosed to have a malignant disease of the blood production (= haematopoietic) system, a so-called

☐ Myelodysplastic syndrome (MDS)

☐ Secondary acute myeloid leukaemia (sAML)

b) What is MDS or sAML?

MDS or the myelodysplastic syndromes are a group of diseases of the bone marrow. Healthy bone marrow produces immature blood cells that then develop into red blood cells, white blood cells and platelets. Bone marrow cells of patients with MDS fail to respond to normal control signals, resulting in a disproportionate number of these cells remaining in the bone marrow instead of maturing and migrating to the blood. Meanwhile, levels of the circulating mature blood cells fall. The mature blood cells, in addition to being few in number, may not function properly due to dysplasia (changes in cell shape and size). Failure of the bone marrow to produce normal cells causes a shortage of normally functioning red blood cells (erythrocytes), white blood cells (leucocytes) and platelets (thrombocytes). Patients with MDS often require blood transfusions and have an increased susceptibility to serious infections and bleeding. In addition, in around 30% of patients diagnosed with MDS the disease will progress to acute myeloid leukaemia (sAML) a type of bone marrow malignancy, which is characterized by a higher blast count (> 30 %) in the bone marrow.

c) Stem cell transplantation for MDS / sAML

A malignant disease like myelodysplastic syndromes or secondary acute myeloid leukaemia can be treated with stem cell transplantation. This means that your disease blood and/or bone marrow cells will be replaced with new blood and/or bone marrow cells.

In the present stage of your disease, the allogeneic stem cell transplantation is – according to the present stage of knowledge – the only therapy with realistic curative prospects. 'Allogeneic' means that stem cells derive from a donor (family donor or voluntary unrelated donor). 'HLA-identical' means, that your potential donor has the same 'tissue pattern' in his cells as you, which is extraordinarily important for the compatibility of the 'foreign stem' cells. In your case we could find an HLA-identical donor, which, in general, enables this kind of therapy to be considered for you.

Prior to the actual transplantation process, the patient receives very high dosages of chemotherapy with or without total body irradiation (radiotherapy). The patient's bone marrow does not survive this intensive treatment, which is why we require the stem cells from a donor to replace the original bone marrow. If successful, these donor cells start to produce healthy stem cells again in the body of the recipient. The immune system or the body's natural defence system uses white blood cells to protect itself from infections. This means that these

cells destroy everything foreign or non-self for example bacteria. This means that transplanted stem cells from a donor would, under normal circumstances, only survive a very short time in the body of another individual. It is, therefore, important that the tissue characteristics of the patient and the donor are identical to avoid immune reactions between donor and recipient white blood cells. Small differences between donor and recipient may cause rejection of the transplant. Alternatively, donor stem cells may react against the body of the patient; this is called reversed immune reaction or graft-versus-host disease. However, the so-called immune competent cells that cause such graft-versus-host disease may also develop a beneficial activity; they have the potential to recognize the malignant, or abnormal cells, and to destroy them.

Stem cells required for transplantation can be collected from the bone marrow or from the peripheral blood of the donor, in this case after administration of a growth factor (G-CSF). It is still not clear which source of stem cells is the preferred source for your disease.

During the past years it has become possible to treat patients with allogeneic transplantations either with bone marrow or peripheral blood stem by dose-reducing the precedent high-dose chemotherapy (so-called conditioning), thus resulting in a lower risk of treatment-related morbidity or mortality but without at the same time endangering the acceptance of the new bone marrow or stem cells. By this lower intensity of the precedent chemotherapy (so-called dose-reduced conditioning), the mortality risk (i. e. to die of treatment-related complications) could be reduced to approx. 20 %. Though this precedent chemotherapy is considerably less intensive than the usual high-dose chemotherapy (hereafter called standard conditioning), it is still strong enough to destroy all malignant and benign cells in the bone marrow. Formerly, this so-called dose-reduced conditioning was given only to those patients who were not eligible for standard conditioning because of their age or concomitant diseases. But after having had good success in these patients with dose-reduced conditioning, the aim of the present study is to evaluate whether in a patient in good general condition (who theoretically could undergo standard conditioning) the same positive results but with less therapy-related mortality can be induced. Therefore, we would like to ask you to participate in this study.

d) Aim of the study

The aim of this study is to show that dose-reduced conditioning will result in less lethal therapy-related complications than standard conditioning but with the same chance of cure. To prove this theory the patient will be treated either with dose-reduced or with standard conditioning prior allogeneic stem cell transplantation. However, to have well-balanced groups of patients in both arms, the patient will be "randomised".

e) Randomisation procedure

If you have a suitable donor available (and all other inclusion criteria are fulfilled) and if you choose to participate to this study, the computer of the European Group of Blood and Marrow Transplantation (EBMT), situated in Leiden (The Netherlands), will randomly decide which form of treatment you should receive. In other words, the computer randomises between the different treatment options (either dose-reduced conditioning or standard conditioning). This is similar to tossing a coin. In order that in both treatment groups the data of the same number of comparable patients will be evaluable, neither you (as patient) nor your treating physician will have any influence on to which group you will belong.

No matter into which group you will be randomised, first the bone marrow or the peripheral blood stem cells from your HLA-identical donor will have to be collected.

f) Chemotherapy (= conditioning regimen)

After the collection of the peripheral blood stem cells / bone marrow from your donor and prior to the actual transplantation process you will receive a chemotherapy with **either** busulfan (12.8 mg per kilogram of your body weight intravenously and 16 mg per kilogram of your body weight orally, resp.) plus cyclophosphamide (120 mg per kilogram of your body weight) (= standard conditioning) **or** busulfan (6.4 mg per kilogram of your body weight intravenously and 8 mg per kilogram of your body weight orally, resp.) plus fludarabine (150 mg per m^2 of your body surface) (= dose-reduced conditioning). In case the donor is not related to you, either anti-thymocyte-globulin (ATG) or the antibody Campath can be additionally given, both of which are drugs that suppress your immune system, enabling the transplanted stem cells from your donor to be better accepted and not to be rejected. Furthermore, both drugs may prevent the graft-versus-host disease, which may occur after transplantation.

The therapy procedure is as follows:

After completion of the preliminary tests (especially cardiac and lung function), you will have a catheter implanted into a big vein, via which you will receive the chemotherapy and the immunotherapy. Both are drugs that on the one hand fight against your disease and on the other hand suppress your immune system, enabling the transplanted stem cells from your donor to be better accepted and not to be rejected. Furthermore, these drugs may prevent the graft-versus-host disease, which may occur after transplantation. The chemotherapy will be given intravenously in both therapy groups (called here therapy arms). Alternatively, the chemotherapy may be given orally in form of tablets.

Arm A (standard conditioning):

You will receive busulfan (12.8 mg per kilogram of your body weight intravenously and 16 mg per kilogram of your body weight orally, resp.) plus cyclophosphamide (120 mg per kilogram of your body weight). The side effects of busulfan and cyclophosphamide are commonly loss of hair (approx. 100 %, rarely irreversible), nausea (approx. 90 %), stomatitis (approx. 80 %), susceptibility to infections and fever due to decrease of leucocytes (white blood corpuscles), as well as a bleeding tendency due to decrease of thrombocytes (blood platelets) (80– 90 %). Skin reactions regarding pigmentation may occur in approx. 80 % under busulfan-therapy. A bleeding infection of urine may occur under cyclophosphamide-therapy (less than 10 % of the cases). The treatment may affect your organs, for example your heart (less than 1 %) and liver (up to 50 % of the cases treated with Busulfan). Generally, transfusions of blood products are necessary. If thrombocytes are low, substitution of thrombocytes will become necessary. As a precautionary measure, you will also receive prophylactic antibacterial, antimycotic and antiviral medication in order to avoid infections.

Arm B (dose-reduced conditioning):

You will receive busulfan (6.4 mg per kilogram of your body weight and 8 mg per kilogram of your body weight orally, resp.) plus fludarabine (150 mg per m^2 of your body surface). The side effects of busulfan are similar to those in Arm A, i. e. especially loss of hair (approx. 100 %, rarely (5 %) irreversible), nausea (approx. 60 – 80 %), mucositis and stomatitis (approx. 80 %), susceptibility to infections and fever due to decrease of leucocytes (white blood corpuscles), as well as a bleeding tendency due to decrease of thrombocytes (blood platelets) (60 - 90 %). Skin reactions regarding pigmentation may occur in approx. 60 - 80 % under dose-reduced busulfan-therapy. In addition, liver affections may occur (approx.

40 - 50 %). Generally, transfusions of blood products are necessary. If thrombocytes are low, substitution of thrombocytes will become necessary. As a precautionary measure, you will also receive prophylactic antibacterial, antimycotic and antiviral medication in order to avoid infections.

In case you will receive bone marrow / peripheral blood stem cells from an unrelated donor, either anti-thymocyte-globulin or the monoclonal antibody Campath may be administered (see below).

What is ATG / what is Campath?

ATG is a rabbit-derived serum, which induces a decrease of certain defence cells, the so-called T-cells. Since it is a foreign protein, side effects such as fever (approx. 90 %), cold shivers (approx. 80 %), increase of the pulse rate (approx. 90 %), decrease in blood pressure (approx. 10 %), and – very rarely (less than 5 %) – also shortness of breath or skin rashes are possible. These reactions will be avoided by a corresponding medication, mainly corticosteroids.

Alternatively, (instead of ATG) the monoclonal antibody Campath-1H can be administered, the effect of which is rather similar to the one of ATG, i. e. it induces a decrease of the lymphocytes, especially of the T-cells. As under ATG, the following side effects may occur: fever (approx. 85 %), cold shivers (approx. 85 %) or increase of the pulse rate (approx. 90 %); in rare cases decrease in blood pressure (20 %) and very rarely (10 %) also dyspnoea or allergic reactions are possible.

Both ATG and Campath have a very long duration in the blood circulation system, thus inducing a prolonged recovery of the immune system after the allogeneic transplantation, so that under this medication the susceptibility to infections (especially viral infections) is increased.

After the pre-treatment you will have the stem cells from your donor injected (= transplanted). In the next 2 – 3 weeks a decrease of leucocytes and thrombocytes will appear, as in autologous transplantations. Fever, infections, stomatitis and (rarely) bleeding may occur. However, after approx. two weeks the stem cells will grow on causing problems like stomatitis and fever to diminish.

However, in both therapy groups (because of the foreign lymphocytes), a so-called graft-versus-host-reaction may arise. In this case the antibodies of your donor will attack some of your organs, especially skin, intestines and liver, causing skin rashes, diarrhoea and – rarely – also (non-contagious) jaundice to develop. Yet, this graft-versus-host-reaction is in fact desirable, as it also works as a defence mechanism, which fights the residual malignant cells. Nevertheless, this defence reaction has to be controlled carefully by application of medication (cyclosporine A). You will have to take this medication also after the transplantation until approx. day 180. Since cyclosporine A may affect the renal system, you have to carefully watch your drinking quantity.

After the bone marrow / blood stem cell transplantation, you will receive two drugs (cyclosporine A and methotrexate), which are supposed to prevent an immune reaction of the donor lymphocytes, the so-called graft-versus-host-reaction. Here the T-cells of your donor, which were transplanted together with the stem cells, affect your body, especially liver, skin and intestines and thus can induce severe organ damage. While methotrexate will be given to you for a total of three days, the other medication (cyclosporine A) will have to be taken by you in form of tablets or fluid for approx. three months after transplantation. The production of leucocytes, erythrocytes (red blood corpuscles) and thrombocytes will reduce for several weeks after transplantation, making the transfusion of erythrocytes and thrombocytes necessary. To accelerate the growth-on of leucocytes you will receive a growth factor (G-CSF = granulocyte colony stimulating factor). This medication is produced gene-technologically and is permitted for this purpose. After the leucocytes (and especially the subgroup of granulocytes) have reached a safe concentration, you will be discharged from the transplantation unit. After that, it will be necessary for you to continue treatment, but on an outpatient basis; frequently at first and less frequently as the therapy continues. This allows post-transplant development to be monitored and the relevant medications to be accordingly adjusted.

g) Donor lymphocyte infusion

Aside from their effect on rejection, the T-cells from the donor stem cells, which were removed by the ATG-treatment, have a positive influence on the destruction of malignant cells. For this reason, you should have another T-cell injection approx. half a year after the first transplantation, after your condition has stabilized. In addition, here, complications in form of a graft-versus-host-reaction are possible, similar to those at the beginning of the treatment. However, the lymphocytes will be given only if a residual disease should be detectable in your bone marrow or blood six months after transplantation or if no complete haematopoiesis through the donor cells has occurred on day 90; and then only if there is not a graft-versus-host reaction.

h) Summary of side effects

The following therapy-related side effects may arise:

- Acute graft-versus-host reaction (aGvHD) with skin rash, serious diarrhoea and severe jaundice (approx. 30 % in case of related donor, approx. 50 % in case of unrelated donor);
- chronic graft-versus-host-reaction (cGvHD) with skin rash, involvement of muscles and junctions, sour mouth, diarrhoea and abnormal liver functions (mild forms: approx. 20 % in case of related donor, approx. 30 % in case of unrelated donor; severe forms: approx. 5 % in case of related donor, approx. 10 % in case of unrelated donor);
- infections during transplantation stage (approx. 80 %);
- Inflamation of the lining of mouth (mucositis), throat, oesophagus (gut), stomach and intestines (approx. 75 %);
- bleeding during the acute transplantation stage (nose bleeding and – rarely – intestinal bleeding) (less than 10 %);
- pneumonia, which may occur during the first three months after transplantation (less than 10 %);
- side effects of ATG (anti-thymocyte globulin): fever (90 – 100 %), cold shivers (80 - 90%), allergic reactions (10 %), rarely changes in production of blood cells and of liver enzymes (less than 5 %);

- side effects of cyclosporine A, such as abnormal function of the kidneys (less than 30 %), depression (less than 5 %), high blood pressure (less than 20 %), convulsions (less than 10 %), nausea (approx. 10 %) and uncommon growth of hair (approx. 40 %);
- long-term side effects of corticosteroids (necessary only in treatment of acute and chronic graft-versus-host-disease) may be: muscle ache, 'moon face', rareweakening of bone structure with possible bone fractures and diabetes.
- infertility and (in women) period stop (amenorrhoea approx. 100 %);
- changes in coloration of the skin under busulfan-therapy (approx. 80 %);
- loss of head hair (90 – 100 % , mostly temporarily, rarely permanent (approx. 5 %);
- rarely damage of cardiac (heart) muscle, pericardial (heart) sac and of coronary (heart) vessels (less than 1 %) (especially under cyclophosphamide = standard therapy arm);
- very rarely damage of kidneys (less than 1 %);
- rejection of bone marrow / blood stem cell transplant; in this case you will receive a second transplant from your donor (les than 5 %);
- increased probability of occurrence of other tumours in the course of the next 20 years (less than 5 %);

In general, the side effects in both therapy arms are very similar, however in the standard therapy arm the side effects may occur more often because of the higher intensity of the chemotherapy.

Despite the possible curative intention of the allogeneic stem cell transplantation, a relapse of the disease may occur. The relapse risk amounts to (depending on the stage of the disease) approx. 15 – 30 %. Generally speaking, it is possible that in the reduced-intensity conditioning therapy arm (Arm B) the relapse risk might be a little higher than in the standard conditioning therapy arm (Arm A).

Despite all precautionary measures, therapy-related and disease-related complications – that although is rarely – may lead to death. According to existing knowledge, the risk of death resulting from dose-reduced allogeneic stem cell transplantation is approx. 10 – 20 %.

Since bone marrow transplantation is a rather new treatment, side effects may theoretically arise that are not yet described. The potential advantages of an allogeneic blood stem cell transplantation compared to conventional therapies are the higher probability of a long-term remission and a cure of the disease.

i) Advantages / Disadvantages of the proposed treatment

The therapy strategies to be checked in this protocol have – in short – the following advantages and disadvantages:

Standard-Conditioning (Arm A):

- Advantage: Because of the intensity of the chemotherapy, less relapses after allogeneic transplantation are probably to be expected.
- Disadvantage: Due to the intensity of the chemotherapy, more therapy-related complications, especially a higher number of treatment-related mortality, are probable.

Dose-reduced Conditioning (Arm B):

- Advantage: Because of the less intensive chemotherapy, less therapy-related side effects and a lower risk of therapy-related mortality are probable.
- Disadvantage: Maybe a higher relapse rate due to the lower intensity of the chemotherapy.

The present study shall prove that the dose-reduced conditioning decreases therapy-related mortality and it will, hopefully, show that no increased number of relapses will occur under dose-reduced therapy (in comparison to standard therapy).

In case you decide not to participate in this study, your treating physicians will recommend that you receive standard conditioning (which is at present the standard therapy in allogeneic transplantation).

Other treatment strategies were discussed with you:

- **application of a further chemotherapy without the prospect of a long-term cure;**
- **transfusion of erythrocytes and thrombocytes; by this the blood parameters will improve; a longer survival on basis of these measurements could not be shown.**

j) Personal Data Privacy Protection

Your treatment data collected during the bone marrow / stem cell transplantation procedure will be filed pseudoanonymously in the European and International Bone Marrow Transplantation Register for improvement of stem cell transplantation methods.

Only authorized and qualified collaborators of the government bodies and of the

Hospital ……………………………………… (Hospital name to be inserted)

have allowed access to the data of this study. The study data will be protected according to the legal requirements of the Data Protection Act and local regulations on patient privacy of the

Hospital ……………………………………… (Hospital name to be inserted)

A unique code number will protect all medical data, collected during the study; we never use the patient or donor full name. No personal data will be used in any study documentation, in reports or publications. Of course, we will inform your general practitioner about the treatment.

k) Insurance

For this study, the EBMT has taken out insurance coverage on your behalf with the following company:

Gerling Insurance Company.

This insurance covers potential damage that may occur as a result of your participation to this study which becomes evident during participation of this study as well as damage that becomes evident within five years after participation.

The insurance does not cover damage:

- Which occurs in offspring as a consequence of a negative impact of your treatment on your genetic material (DNA damage)
- Which was very likely to occur in view of the pre-treatment tests
- Which would have occurred if you would not have participated to the study

In order to keep the right of damage compensation it is important for you to follow the instructions of the investigators.

In case you believe to have suffered damage by your participation in this study, you should report this as soon as possible to the Insurance Company. You should provide all necessary information to the insurance company. Omission of this obligation may lead to loss of damage reimbursement.

l) Conclusion

You are completely free to decide whether you wish to, or not, to participate in this study. In case you decide to participate in the study, you may always withdraw at any time without explanation. However, a discontinuation of participation after the application of high-dose chemotherapy and before the transplantation of the blood stem cells will, with a very high probability, lead to death.

Whatever you decide, it will never change anything in the care and support of you and your family. Your treatment will always be planned and performed according to the treatment plan. It is, of course, possible that unforeseeable circumstances will require a change in the treatment plan if there are new developments in your disease or general condition. This will be discussed with you in order to allow you to reconsider your participation in the study. Your treating physician may decide, if necessary, to stop the treatment according to the study.

If you agree to participate in the study, you will be asked to sign the consent form of this study. By signing this form you confirm that you are sufficiently informed about the procedures required from you and that you agree to participate in the study.

If you wish, you can ask for independent advice about this study. In that case, you may contact:

Dr. ……………………………… Phone …………………..……….

If you have additional questions, you may always contact your treating physician or one of the study coordinators:

PD Dr. Nicolaus Kröger
Bone Marrow Transplantation
University Hospital Hamburg-Eppendorf
Martinistraße 52
20246 Hamburg / Germany
Phone +49-40-42803-4850/-4851
Fax +49-40-42803-3795
E-mail: nkroeger@uke.uni-hamburg.de

Prof. Dr. Theo de Witte
Department of Haematology
University Medical Center
PO Box 9101
6500 HB Nijmegen / The Netherlands
Phone: +31-24-361-8810
Fax: +31-24-354-2080
E-mail: L.Brinkman@hemat.umcn.nl

The Chronic Leukaemias Working Party

MDS-Subcommittee

Dose-reduced versus standard conditioning followed by allogeneic stem cell transplantation in patients with MDS or sAML: A randomised phase III study

**Patient's Name, Surname and Date of Birth (or patient's label)**

26.13.2 PATIENT'S WRITTEN INFORMED CONSENT

	YES	*NO*
I have read the patient information sheet for this trial and have received a copy to keep.	☐	☐
I have been given the opportunity to ask questions about the trial and have received satisfactory answers to all of my questions.	☐	☐
I am aware that my participation is voluntary and that I am free to withdraw at any time, without giving any reason, without my medical care or legal rights being affected. However, I know that a discontinuation of participation after the application of high-dose chemotherapy and before the transplantation of the blood stem cells with very high probability will lead to death.	☐	☐
I accept that some of the blood and bone marrow samples taken may be used for biomedical research.	☐	☐

I give permission for responsible individuals from the trial research team and regulatory authorities to review my medical records. ☐ ☐

I understand that information that identifies me will be kept confidential to those concerned with my care and the trial research team. ☐ ☐

I give permission for the pseudoanonymised information provided for the trial to be used in future medical research. ☐ ☐

I agree to take part in the above study. ☐ ☐

Patient:

Name: …………………………. Signature …………………… Date: ……….

(Name in Block Letters)

Investigating doctor:

Name: …………………………. Signature ………………….. Date: …………

(Name in Block Letters)

Witness:

Name: …………………………. Signature ………………….. Date: …………

(Name in Block Letters)

26.14 PARTICIPATING CENTERS

CIC	Hospital	City	Investigator	Country
271	UnivKlin f Innere Med	Innsbruck	D. Nachbaur	Austria
227	AKH Universitats Kliniken	Vienna	H. Greinix	Austria
234	Clin Univ Saint Luc	Brussels	A. Ferrant	Belgium
718	Charles Univ Hosp	Pilsen	V. Koza	Czech Republik
515	Helsinki Univ Central Hosp	Helsinki	T. Ruutu	Finland
650	CHRU	Angers	P. Guardiola	France
252	Hosp Henri Mondor	Creteil	C. Cordonnier	France
259	Univ Hosp	Essen	H. Biersack	Germany
614	Univ Hosp Eppendorf	Hamburg	N. Kröger	Germany
295	Univ Hosp	Hannover	B. Hertenstein	Germany
524	Univ Hosp	Heidelberg	P. Dreger	Germany
256	Univ Hosp	Kiel	M. Scheel-Haus	Germany
389	Univ Hosp	Leipzig	K. Al-Ali	Germany
513	Univ Hosp	Munich	C. Schmid	Germany
680	Univ Hosp	Munster	M. Stelljes	Germany
754	Chaim Sheba	Tel-Hashomer	A. Nagler	Israel
658	Ospedali Riuniti	Bergamo	A. Rambaldi	Italy
240	Inst.Hemat&med.oncol	Bologna	S. Orsola-Malpighi	Italy
304	Caressi Hosp.	Firenze	S. Guidi	Italy
616	Univ Hosp	Milano	P. Corradini	Italy
265	IRCCS osp.Maggiore	Milano	G. Lambertenghi	Italy
237	Radboud University hospital	Nijmegen	T. de Witte	NL
725	SPB Pavlov Medical Univ	St. Petersburg	B. Afanassiev	Russia
260	Hosp de la Santa Creu	Barcelona	R. Martino	Spain
289	Sahlgrenska Univ H	Goeteborg	M. Brune & A. Olsson	Sweden
202	Kantonsspital	Basel	A. Gratwohl	Switzerland
763	GKT School of Medicine	London	G. Mufti	UK
780	Christie Hospital	Manchester	E. Liakopoulou	UK

26.15 COUNTRY COORDINATORS

Country	Name	City
Austria	H. Greinix	Vienna
Belgium	A. Ferrant	Brussels
Czech Republic	V. Koza	Pilsen
Finland	T. Ruutu	Helsinki
France	P. Guardiola	Nantes
Germany	N. Kröger	Hamburg
Israel	A. Nagler	Tel-Hashomer
Italy	G. Lambertenghi	Milano
Russia	B. Afanassiev	St. Petersburg
Spain	R. Martino	Barcelona
Sweden	M. Brune	Gothenborg
Switzerland	A. Gratwohl	Basel
The Netherlands	T. de Witte	Nijmegen
UK	G. Mufti	London

26.16 DOCUMENTATION SHEETS

The CRF's are available on the EBMT/CLWP website as separate documents for registration, diagnosis, pre-transplant, transplant, +100 days, follow up and SAE.

http://www.ebmt.org/5WorkingParties/CLWP/clwpct.html
